# Streams as Entanglement of Nature and Culture: European Upper Paleolithic River Systems and Their Role as Features of Spatial Organization

**DOI:** 10.1007/s10816-015-9263-x

**Published:** 2015-10-07

**Authors:** Shumon T. Hussain, Harald Floss

**Affiliations:** 1 Paleolithic Research Unit, University of Cologne, CRC 806 “Our Way to Europe”, Bernhard-Feilchenfeld-Str. 11, 50969 Cologne, Germany; 2 Faculty of Archaeology, Leiden University, Einsteinweg 2, NL-2333CC Leiden, The Netherlands; 3 Department of Early Prehistory and Quaternary Ecology, University of Tübingen, Burgsteige 11, Schloss Hohentübingen, 72070 Tübingen, Germany

**Keywords:** Pleistocene river systems, Upper Paleolithic spatial organization, Ecocultural systems, Nature-culture entanglement, Affordances, Focality

## Abstract

Large river valleys have long been seen as important factors to shape the mobility, communication, and exchange of Pleistocene hunter-gatherers. However, rivers have been debated as either natural entities people adapt and react to or as cultural and meaningful entities people experience and interpret in different ways. Here, we attempt to integrate both perspectives. Building on theoretical work from various disciplines, we discuss the relationship between biophysical river properties and sociocultural river semantics and suggest that understanding a river’s *persona* is central to evaluating its role in spatial organization. By reviewing the literature and analyzing European Upper Paleolithic site distribution and raw material transfer patterns in relation to river catchments, we show that the role of prominent rivers varies considerably over time. Both ecological and cultural factors are crucial to explaining these patterns. Whereas the Earlier Upper Paleolithic record displays a general tendency toward conceiving rivers as mobility guidelines, the spatial consolidation process after the colonization of the European mainland is paralleled by a trend of conceptualizing river regimes as frontiers, separating archaeological entities, regional groups, or local networks. The Late Upper Paleolithic Magdalenian, however, is characterized again by a role of rivers as mobility and communication vectors. Tracing changing patterns in the role of certain river regimes through time thus contributes to our growing knowledge of human spatial behavior and helps to improve our understanding of dynamic and mutually informed human-environment interactions in the Paleolithic.

**Electronic supplementary material:**

The online version of this article (doi:10.1007/s10816-015-9263-x) contains supplementary material, which is available to authorized users.

## Introduction

Humans are spatial beings and the nature of our spatial presence is in many ways crucial for what we are. How we define our relationship to the world and how we understand and access what surrounds us is an important cornerstone of our different lifestyles. Space utilization (*Raumnutzung*), therefore, is a central research area for all the human sciences (*e.g.*, Dünne and Günzel 2006; Warf and Arias 2009; Bachman-Medick 2010). Archaeologists, consequently, have ever since attempted to tackle the “spatiality” of past human social units from a whole range of different angles (*e.g.*, Shott 1986; Kelly 1992; Close 2000; Brantingham 2006; Bernard and Wendrich 2008; Grove 2009, 2010; Turq *et al.* 2013; Cameron 2013; Van Dommelen 2014). Yet, how anchoring key notions such as “space” need to be approached is highly disputed and remains a very sensitive matter.

While many scholars dealing with post-Pleistocene societies tend to emphasize the conceptualized and “built” nature of each “anthropospace” (*e.g.*, Tilley 1994; Ashmore and Knapp 1999; Muir 2000; Meskell and Preucel 2004; Lang 2009; Hanks and Linduff 2009), Paleolithic archaeologists usually focus on the “natural” dimension of spatiality, on the environment *sensu stricto*, tacitly assuming that spatial presence in general is *before anything else* driven and crucially constrained by bioecological regimes (*e.g.*, Van Andel and Davies 2003; Verpoorte 2009; Müller *et al.* 2011; Richter *et al.* 2012; Hertler *et al.* 2013; Harcourt 2012, p. 77; Arrizabalaga *et al.* 2013). It is not our aim here to contest the undeniable contribution of ecology and physiography. We rather believe, however, that it is time now for Paleolithic archaeology to move beyond that one-sided paradigm. After all, there is good reason to believe that spatial behavior is in fact a product of the *dynamic interplay* of both naturally given and lived and experienced properties of the landscape.

From this perspective, this paper attempts to overcome two long-standing assumptions that seem to characterize the way many Paleolithic archaeologists have approached spatial behavior in the deep past: (1) human spatiality is primarily adaptation and therefore largely *dictated* by environmental conditions, and (2) human spatiality is the result of *all* factors that define a given environment and can thus be framed as a “strategy” to deal with the entirety of background conditions. Our aim, however, is not to disqualify these statements *in omnia*, but rather to demonstrate that they can be substantially enriched by a perspective that acknowledges a critical interlacing of ecophysiographic landscape properties and different sociocultural fabrics. Arguably, it is this particular co-constitutive interface which is important to discuss, and ultimately decisive to understand.

We use the example of large Pleistocene river systems and mobile Upper Paleolithic hunter-gatherer societies in different parts of Central and Western Europe to show how specific sociocultural contexts can give rise to the spatio-organizational significance of specific and individual landscape features with their own discrete and significant properties. The environment as a whole remains always important of course, simply because it provides the overall spectrum of potential prey species and collectable plants, for example, but single natural features and/or formations such as river catchments can offer rather unique behavioral possibilities *by their own virtue* and can consequently “entrap” people in meaningful behavioral trajectories that are not simply reducible to the environment totality. It follows that specific dimensions of the archaeological record might actually be the result of the severe impact of single landscape features and therefore, by implication, much more convincingly explainable by the latter.

The first part of the paper carefully develops the methodological and conceptual instruments to appropriately approach space in general and large Pleistocene river systems in particular. It is suggested that humans are “entangled” not only with material culture but also with spatial entities and specific landscape features and that the “ecocultural system” theory might be one fruitful avenue to account for this state of affairs. River systems are then parts of such historically situated ecocultural systems. Moreover, their specific ecological and biophysiographic properties often immediately attract attention and motivate certain behaviors. It is thus suggested that the biased nature of spatial cognition, the regular use of “spatial heuristics,” and the presence of strong “affordances” are ways to explain such an entrapment. Additionally, the spatial prominence of many river systems is an important framework in which these issues can be fruitfully discussed. We then move to the nature of Pleistocene river systems in Europe and discuss the relationship between their unique characters and the kinds of space utilization these might provoke. In the second part, finally, we try to show how these varying impact-vectors of Pleistocene river systems actually affect archaeological patterning in the European Upper Paleolithic. After all, the analysis reveals that large rivers had in fact a tremendous impact on Upper Paleolithic spatial organization on many different scales and in many different ways. Our conclusions also emphasize that this impact can only be fully understood if we relate the characteristics of each single river meaningfully to the sociocultural context in which it is encountered, experienced, and narrated.

## Biases in Human Spatial Behavior

Why are rivers important? Even large river systems are only a fragment of the landscape entirety in which they are embedded. While this statement is, of course, a truism in itself, it is important to note that human behavior is at the same time often significantly biased by a small set of environmental factors or even single spatial features. We tend to assume that climate, geology, vegetation, faunal communities, and their “behavior” in a given setting are *altogether* cognitive, or at least evolutionary, inputs that adjust the human spatial performance for a good fit with all of these variables at the same time. Recent developments in psychology and human ethology, however, suggest that this is only partially true. Single landscape features and/or formations can “entrap” people into specific behavioral trajectories without necessarily referring to their wider bioecological context. Two useful notions that may lay out the conceptual foundation for river systems providing such powerful behavioral clues that attract and detract people in critical ways are “heuristics” and “affordances.” We shortly introduce these concepts and discuss them in relation to large Pleistocene river systems to set the stage for a more thorough analysis of the latter’s role in constructing the sociocultural space of different European Upper Paleolithic units.

### Heuristics

The traditional view of the relationship between environmental background and human spatial behavior is, broadly speaking, one of analytical inference (*e.g.*, Simon 1993; Kunz 2004). In a nutshell, the totality of the available information is compiled, processed, and finally “turned” into a proper behavioral answer, whereas “properness” is defined as some sort of Cartesian rationality (*cf*. Adair 2007; Newell *et al.* 2007). The main problem with this view is its ignorance of differential information availability and the expenditure of information extraction in particular spatial settings (Gigerenzer and Gaissmaier 2011). Humans depend in a variety of situations—especially if they have little landscape knowledge—on fast but reliable decision-making that can easily be rooted in simple environmental clues (Binmore 2009; Sorros 2009, p. 6; Stiglitz 2010, p. 243). Such “rules of thumb” are called heuristics (Michalewicz and Fogel 2000; Gilovich *et al.* 2002; Gigerenzer *et al.* 1999, 2011; Gigerenzer and Selten 2002; Bishop 2006; Gigerenzer and Gaissmaier 2011).

Heuristics are simple and often intuitive behavioral rules (Gigerenzer 2007) that rely only on a small fraction of the available environmental “evidence”—they are frugal. They are also fast-performing, allowing for quick behavioral judgments. Critically, however, at least some of these “fast and frugal” heuristics (FFHs) are very reliable (Dawes and Corrigan 1974; Einhorn and Horgath 1975; Gigerenzer 2008; Bishop 2006; Hogarth 2012; Busemeyer 2015), yielding outputs that are about as accurate in the long run as nonfast, nonfrugal behavioral rules favored by the traditional view. At the heart of FFHs stands the “less is more principle” (Kahneman *et al.* 1982; Czerlinski *et al.* 1999; Gigerenzer and Goldstein 1996; Brighton 2006; Chater *et al.* 2003; Gigerenzer and Brighton 2009) and the insight that certain properties of the environment are already captured by certain properties of individual features that are situated in this environment (Gigerenzer 2008; Gigerenzer and Gaissmaier 2011, 474f.).

The location and availability of key resources, for example, might already be signaled by the *presence* of large river systems because they usually carry and provide stony raw material, and because water is a critical resource for many, if not all, potential prey species. In this sense, such features offer “informational shortcuts” and/or are proxies for specific aspects of the landscape that would otherwise be difficult to infer. The same argument can be made for the issue of landscape accessibility. Because large rivers travel over vast spatial distances and have flattened parts of the landscape in the process, they are generally a good heuristic for landform interconnectivity. This property can be considered a good candidate for a generalized “follow the river” heuristic. All in all, the combination of several reliable clues that indicate, for example, resource availability and travel convenience grants larger rivers a significant ability to bias and, by implication, guide human spatial behavior—be it today or in the past. Alongside the easy recognizability of large river systems in the landscape, they might even be considered a special case of the “recognition heuristic” (*cf*. Goldstein and Gigerenzer 1999), which is known to offer a robust rule of thumb for a whole range of different behavioral problems (Bishop 2006).

In this way, rivers constitute an important cornerstone of a “bounded” or “ecological” perspective on decision-making and judgment in space (Simon 1989; Gigerenzer and Goldstein 1996; Gigerenzer and Selten 2002; Kahneman 2003; Grüne-Yanoff 2007; Todd *et al.* 2012). Viewed in this light, heuristics can even be seen as integral components of the human “adaptive toolbox” (Gigerenzer *et al.* 1999; Boudry *et al.* 2015; Polonioli 2015). They provide behavioral guidelines *by their own virtue*, which is by no means “mystical,” but grounded in the specific nature of each river-environment relationship.

### Affordances

In many ways, affordances take the same line. As an explanatory concept, the term first emerged in perception psychology in the late 1980s and goes back to James J. Gibson’s seminal book *The Ecological Approach to Visual Perception* (1979). The core idea is that the formal properties of the environment are not neutral givens, but “actively” constrain and control behavior. Simultaneously, though, the environment is not seen as a mere “collection of causes that pushes the animal around” (Withagen *et al.* 2012). Each organism that lives in a specific environment encounters a formal “architecture” that is particular to that environment and offers very specific *action possibilities* (Gibson 1979; McGrenere and Ho 2000). Approached from this perspective, behavior is not simply forced upon organisms by external circumstances but negotiated between ecophysiographic configurations of environments and the organism’s way of life, including its biological makeup (Chemero 2003). It is important to note that this view explicitly rejects both mechanistic and entirely arbitrary characterizations of the human-environment relationship that “produces” behavior on fundamental theoretical grounds. The key aspect here, however, is that environment-specific affordance structures that can be perceived and discovered often *suggest* certain behavioral strategies that exploit that “architecture”; that is, people are immediately drawn to act in certain way. In this sense, the configuration of the environment can critically invite and stimulate behavior (Withagen *et al.* 2012).

Like a door usually affords passage or a chair affords sitting, it is reasonable to claim that large river systems in specific environments afford being followed by mobile human groups. Only in this sense is it plausible to speak of rivers as “natural pathways” that entrap people in certain behavioral trajectories—in this case, in certain mobility patterns.

Both heuristics and affordances call attention to the human-environment relationship as the proper unit of analysis to fully understand spatial behavior (Gibson 1979; Chemero 2003; Stoffregen 2003; Withagen *et al.* 2012). The relationship between the two, however, is difficult to discern. It is possible, for example, that both the use of certain heuristics and the exploitation of specific affordance structures have a deep evolutionary history (*e.g.,* Withagen and Van Wermeskerken 2010). Yet, it seems unlikely that both regulative domains of human spatiality became fixed at some point and remained unsusceptible to major reorganizations of their sociocultural context. Both heuristics and affordances only yield valuable behavioral results as long as they “serve” and “support” the socioculturally informed lifestyle that draws on them. In this regard, we favor a mutualistic perspective that considers heuristics and affordances as interrelated and thus potentially amplifying each other under favorable circumstances. These circumstances, as well as the critical coupling of ecophysiographic properties of the environment and the sociocultural fabric of Pleistocene hunter-gatherer communities, will be discussed in the following sections. We start with a short, but important discussion of the notion of space and its irreducible two-sided nature.

## Space as a Nature-Culture Entanglement

Space has lost its innocence. It has also lost its intuitive meaning. We can no longer retain the idea that space is identical with the environment, the landscape or even with everything that has an objectifiable “place” in the world around us. Already Immanuel Kant insisted on the transcendental argument that although space is always given—entities and things are unescapably *located*—it can only be grasped through the lenses of experience rooted in a particular lifeworld. Therefore, it has become difficult to defend a purely naturalistic stance toward space that encompasses only what is intuitively conceived as spatial, namely the physiographic and ecoclimatic dimensions of particular spatial settings. Such a naturalistic view has been traditionally influential in Paleolithic archaeology (*cf*. Butzer 1982; Albarella 2002; Hockett and Haws 2005), however, partly because geomorphological and climatic reconstruction of the deep past are considered to be more reliable than the often ambiguous material remains and partly because it is widely believed that severe Ice Age conditions have strongly constrained what forager groups living in these settings could actually do (*e.g.*, Hoffecker 2002; Anderson *et al.* 2007; Sirocko 2009). As already acknowledged, it is indeed important to recognize the constraining properties of many Pleistocene environments, but it still remains true that many equally important constraints emerge from specific systemic configurations pertaining to the sociocultural world of Pleistocene hunter-gatherer groups. It is thus essential to integrate all the different dimensions of past human spatial reality—nature and culture—under one unified perspective in order to understand spatiality in the past. Hence, it is important to embrace more holistic and co-constitutive stances toward space: a theoretically informed position that substantially overcomes simple naturalistic reductionism and determinism. Below, we give a short outline why such a paradigm shift is desperately needed in Paleolithic archaeology, and why a naturalistic definition of space often fails to account for the richness of the Pleistocene archaeological record.

First of all, a strict naturalistic view of space encompasses only what has been termed “environment,” “ecospace,” “natural landscape,” or “natural space” (*cf*. Albarella 2002; Reitz and Shakley 2012). Recent findings, however, demonstrate that human presence adds considerable dimensions to spatiality, since humans, past or present, always *relate* to their surroundings (*e.g.*, Bollnow 1997; Bonnemaison 2005). They interact, modify, and transform spatial features in various ways (*cf*. Rossignol and Wandsnider 1992; Basso 1996; Nicholson and O’Connor 2000; Oetelaar and Oetelaar 2007), as well as ascribe meaning and significance (*cf*. Tilley 1994; Ingold 1993, 2000, 2011; Rockman 2003, p. 4; Hansen and Meyer 2013). The latter factor seems to be a crucial part of the human condition—of each “anthropospace,” so to speak—for meaning and significance are “devices” of worldly guidance helping to *organize* different lifeways (Ashmore and Knapp 1999; Lang 2009, p. 32; Hansen and Meyer 2013). The modalities of relating to the world are thus never given, but rather influenced by both individual and collective experiences as well as sociocultural conceptualizations often rooted in narratives and complex mythologies (*cf*. Ingold 2000, 40ff., 57f.).

This idea largely dates back to the Continental European tradition of philosophical inquiry, broadly characterized as phenomenological, and most prominently advocated by Martin Heidegger and Maurice Merleau-Ponty (*cf*. Cataldi and Hamrick 2007). Both argue that humans are before anything else “beings-in-the-world” (Heidegger 1927a, b; Merleau-Ponty 1945, 1958, 1964). They are thrown into *predefined spatial settings*, enriched by the respective cultural traditions and underpinned by the physiographic properties of the surrounding landscapes. Perception, experience, and behavior thus emerge from the complex interplay of natural environments and cultural systems. Consequently, it is the phenomenology of a particular setting that becomes central to human spatial dwelling and the resulting archaeological patterning.

Accordingly, “spatial turn” advocates (*cf*. Dünne and Günzel 2006; Döring and Thielmann 2008; Günzel 2009; Warf and Arias 2009; Bachman-Medick 2010) have convincingly argued for the “socially constructed” and “culturally built” dimension of space emphasizing the human factor therein (*e.g.*, Ashmore and Knapp 1999; Muir 2000; Lang 2009). Each spatial feature has therefore to be understood as an *entanglement* of natural properties and sociocultural dimensions (*e.g.*, Gamble 1993; Tilley 1994; Rockman 2003; Meskell and Preucel 2004; Edgeworth 2011; Hodder 2012). As a result, the specific and potentially changing *place* of individual spatial entities within larger “ecocultural systems” (*sensu* Rapport and Maffi 2010, p. 104; see also Pretty and Pilgrim 2010 and references therein) can be extremely informative.

Secondly, the methodological primacy of natural properties within wider landscape archaeological studies underestimates the contingency of the nature-culture divide in human societies (Joseph *et al.* 1990; Descola 1992, 1996, 2011; Porr and Bell 2012). Most hunter-gatherer groups, for example, have developed individual ontologies guiding their relationships with (other) worldly and nonworldly entities, may they be animals, plants, things, or other humans (*cf*. Casimir and Rao 1992; Descola 2011). Projecting our own, Cartesian conceptualization of worldly affairs is thus epistemologically naive at best (Joseph *et al.* 1990; Scarso 2013). While western societies tend to naturalize space, hunter-gatherer groups often animate spatial settings, equating human and nonhuman agents as both being active players (Bird-David 1999; Ingold 1987, 2000, 114ff.; Descola 2011; Hussain 2013, 84ff.). It is in this context, that individual natural features gain special significance because they are regularly conceived as *persons* and are thus experienced as meaningful encounters (Casimir and Rao 1992; Andrews 1994; Strang 1997). Such entities—as for example epitomized by the *Uluru* (Ayers Rock) in the context of Aboriginal spatiality—can become important reference features in space structuring the movement of people and entire groups (*cf*. Morphy 1995; Schama 1995; Tilley 1994; Thomas 1996, 2001).

In sum, nature and culture seem to be spatially inextricably interwoven, and both dimensions of spatiality have to be taken into account when human-environment interactions in the past are being discussed (Flemming 2006; Wilcock *et al.* 2013). Most notably, single natural features that are being conceived as *persona* with particular characteristics can become exceptional enough to significantly bias and shape how humans behave spatially. Clearly, it should be possible to track these entanglements in the *longue durée* by systematically documenting the spatial, temporal, and material relationship between exceptional physiographic features and different archaeological archives (compare Raeder 1993; Kaufmann 2004; Bintliff 2006; Lang 2006; Hodder 2012).

Before turning to large river systems, which have frequently been hypothesized to possess such exceptionality due to, for example, their flow properties clearly separating them from other static features of the environment (*e.g.*, Edgeworth 2011), we want to suggest that “exceptionality” in this sense can be framed as “focality.” Discussing rivers as focal spatial features can then serve as a methodological baseline for evaluating their spatial significance through time and helps to theorize the merits of analyzing their spatiotemporal relationships with material culture archives.

## Focality as a Perspective for Addressing Spatial Significance

What grants an individual spatial feature the status of exceptionality and, therefore, the ability to shape and considerably bias human spatial behavior? We have argued so far that both formal properties with specific affordance structures as well as socioculturally attributed properties have to be taken into account when Paleolithic archaeologists attempt to identify such spatial features. It is time now to suggest that exceptionality in this sense can be conceived as *focality* (*cf*. Schelling 1981; Sugden 1995; Sugden and Zamarrón 2006; Binmore and Samuelson 2006). As we will see, using the concept of focality will help us to better understand the role of larger river systems in spatial organization and in spatial coordination in particular.

The notion of focality is a product of modern economic anthropology and was first introduced to the scientific landscape by Thomas Schelling in his famous book *A Strategy of Conflict* (1981). In contrast to most competing game theoretical approaches, which are quantitative in nature (*e.g.*, Winterhalder 1986; Kaplan and Hill 1992; Bird and O’Connell 2006; Lupo 2007), focality can be described as a deeply *qualitative* solution to human coordination problems in general (Schelling 1981). Hence, it can much better account for the hierarchical, heuristic, and biased nature of human decision-making at the interface of nature and culture. If we further accept that spatial behavior is, at least to a considerable degree, coordination in finite space—that is, people organizing their presence, actions, and movements in a limited area—focality can become a key driver of human spatiality.

In opposition to other approaches in the field, the focality-solution to coordination problems is largely independent of strong and questionable suppositions such as applied homogeneous logic and rationality through time and space. The central idea is that the *structure* of each context is indication enough for successful coordination (Schelling 1981). Each context presents features and properties that are exceptional in that context, meaning that they are outstanding and prominent. Such features and properties can become strong reference points when social players try to coordinate their behavior (Schelling 1958, 1981, p. 57). A simple example is a situation in which several people are unable to communicate with each other but are committed to select the same card from a whole set of cards. Dependent on their knowledge about each other and the structure of the set, solution finding can differ in complexity. If the set of cards contains only one single black specimen whereas all the others are red, for example, the solution is rather obvious. The same principle applies when people have lost each other in a crowded city center and almost miraculously reconvene at the next bus or underground station. “Focal points” are therefore prominent features within a given context, often characterized by uniqueness, analogousness, asymmetry, intuitiveness, immediacy, conventionality, centrality, esthetic appearance, or special visuality (Schelling 1981, 57f.), that allow for “fast and frugal” coordination. From this perspective, focality is essentially a coordination-heuristic that integrates landscape affordances and sociocultural horizons in a meaningful way.

**Fig. 1:**
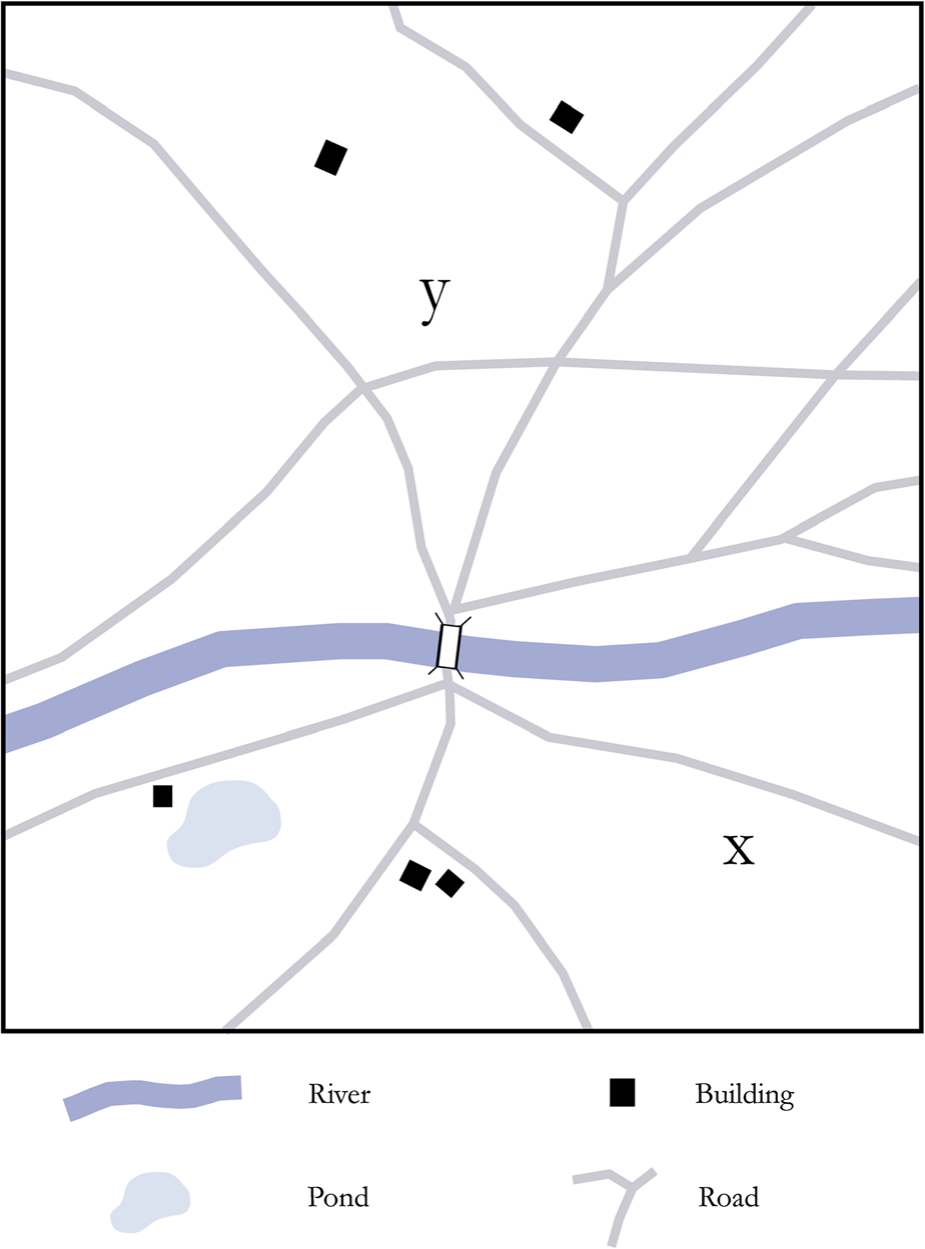
Coordination scenario discussed by Schelling in his famous book *A Strategy of Conflict* (1981): *y* and *x* mark hypothetical landing positions of two conflicting and heavily armed parties. The framework is defined as an “all or nothing” condition, where contact between the two symmetrical parties will lead to the extermination of both. The objective is therefore to coordinate in space by claiming as much ground as possible while simultaneously avoiding contact and by implication potentially deadly conflict. Schelling shows that the river is the only feature in this scenario which has the ability to “enable” successful coordination, even though it does not divide the available landmass into two identical (or symmetric) parts (redrawn from Schelling 1981: Fig. 7)

Schelling (1981, 55f.) himself has argued that in many spatial settings, large rivers are focal features in this sense. In particular, he has demonstrated that when risk is relatively high and human groups have to subdivide a certain area between them, rivers are very likely to become segregating features regardless of both their shape and their relative position within the disputed area (Schelling 1981: Fig. 7; Fig. 1). This finding, again, seems to be tightly connected to both their prominent physical and phenomenological quality within most spatial settings. They are therefore potentially powerful “devices” for *affording* behavior and *channeling* cultural meaning and significance. Because focality has to be understood as a strictly contextual quality of spatial features, however, it is important to first explore the specific environmental and social framework of Late Pleistocene human-river relationships. The next section is dedicated to this task and attempts to offer some general reasons why major Pleistocene river systems deserve special attention in this respect and should be discussed as potentially very prominent landscape elements.

## Ecology, Corridors, and Spatial Meaning in the Pleistocene

When discussing rivers and their role in Pleistocene land-use systems, it is important to acknowledge that most of today’s river regimes deviate significantly from their pristine state due to intense anthropogenic impact (Petts *et al.* 1989; Ward *et al.* 2002). Moreover, Pleistocene rivers display a high degree of geomorphological plasticity and have frequently changed their appearance, for example, in relation to the various climatic regimes (*cf*. Smithson *et al.* 2002, 302ff.; Anderson *et al.* 2007, pp. 210–213). Here, we identify key physiographic and ecological parameters of Pleistocene river systems and discuss them in relation to ethnographically documented modalities for relating to such features. These insights can then serve as a baseline to discuss the relationship between these parameters and spatial prominence, intrinsic affordance structures, heuristic action values as well as sociocultural organization in the Upper Paleolithic of Central and Western Europe.

### Geomorphological and Ecological Gradients

**Fig. 2:**
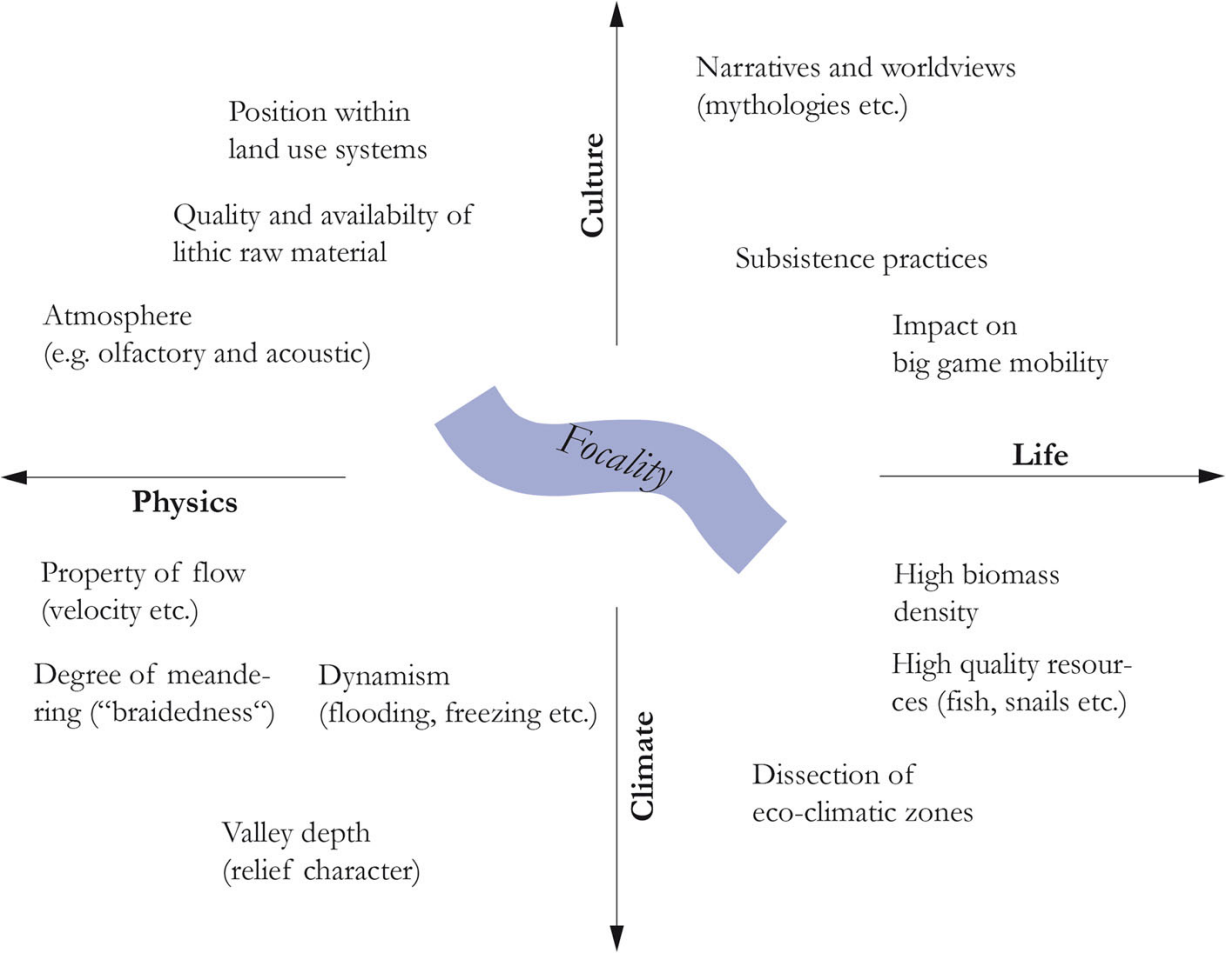
Focality as an emergent property of ecocultural systems rooted in a broad range of factors and their relationships. Natural factors are equally as important as cultural ones, leading to a view which takes into account the spatial quality of a river as nature-culture entanglement. The resulting “personality” is the baseline for a river’s environmental legibility and how people actually read and interpret it. Focality can thus be understood as the disposition of certain river courses to bias human spatial behavior in drawing attention from other landscape features and in anchoring behavior. *Vector arrows* indicate that the four categories overlap substantially

Pleistocene river systems are in most cases massive landforms, including not only the water body itself, but rather the entire valley corpus with lateral terraces and often steep topographic gradients as well as endemic faunal and floral communities (Smithson *et al.* 2002; Ward *et al.* 2002; Hilty *et al.* 2003). When combined, these features generate unique spatial settings that are highly recognizable within the wider landscape. This is particularly true when river systems are embedded into glacial tundra-steppe environments with near-absent or at least significantly reduced vegetation cover (Kelly 2003; Hussain and Floss 2014). Such visual prominence survives even in interglacial settings where most of the coniferous patches flank river courses, setting them apart from other landforms. Furthermore, Pleistocene river systems represent areas of high biomass density compared to their surroundings (*cf*. Wohl 2004; Tockner *et al.* 2006). In combination with both freshwater and flint/chert availability, they are clearly attractive to hunter-gatherer populations living in the surroundings. Many of these large river systems also coincide with the primary mobility and migration vectors of certain animals and big game species (*cf*. Stutz *et al.* 1995; Planty-Tabacchi *et al.* 1996). Paleolithic foraging groups will therefore likely adapt to this pattern (*e.g.*, Mellars 2004) and consequently perceive the respective fluvial regimes as important channeling factors (*cf*. Edgeworth 2011, p. 121). Finally, rivers are regularly coextensive with the margins of different biomes, ecotones, or climatic zones (Malanson 1993, 205f.; Haslam 2008, 260ff.). As a result, they commonly offer extremely favorable conditions (*cf*. Ayres and Clutton-Brock 1992; Goodman and Ganzhorn 2004; Hayes and Sewlal 2004; Anthony *et al.* 2007; Toivonen *et al.* 2007; Arzamendia and Giraudo 2009; Kondo *et al.* 2009; Nicolas *et al.* 2011; Fernandes *et al.* 2012) that both animals and humans can exploit. The formal spatial constellation associated with most of Europe’s large Pleistocene river regimes thus marks a clear rupture with other landscape features and contributes to a focal disposition of such river systems (Fig. 2).

### Fluvial Corridors

Rivers are the most important and most extensively studied natural corridors (Stanford and Ward 1993; Ward *et al.* 2002; Hilty *et al.* 2003; Farina 2010, pp. 150–156). They are often favored passageways for various animal taxa and connect different biome patches or bioecological units (Jongman 1998). Planty-Tabacchi *et al.* (1996), for example, have recently documented a great number of nonnative species moving along different river regimes. From this perspective, fluvial corridors have to be seen as spatial features that invite living organisms to use them as conduits. They, therefore, embody strong *affordances* to channel movement and migratory activity in general. Natural corridors are useful spatial heuristics that help piloting in space, in particular when landscape knowledge is limited and “fast and frugal” reasoning becomes crucial. River corridors are thus exceptional landscape features and are *experienced* as such by humans that encounter them for the first time or live in their immediate vicinity.

### Dynamics of River Regimes

When considering the focality of Pleistocene river systems, fluvial and geomorphological *dynamics* are key variables. Rivers are by far the most dynamic features in Pleistocene environmental settings (*cf*. Fuller *et al.* 1998; Lewis *et al.* 2001; Antoine *et al.* 2003; Benito *et al.* 2003; Westaway and Bridgland 2010) and constantly change both their appearance and their fluvial profile (Lewis *et al.* 2001; Vandenberghe 2001, 2002; Ward *et al.* 2002). River cyclicality, however, is expressed on different spatial and chronological scales and can therefore vary even within the same river system (Brierley *et al.* 2013). It is important to note that while river fluctuations can be documented on daily, seasonal, annual, and millennia rhythms, microscale oscillations can hardly be expected to affect archaeological patterning in a significant way. They may, however, contribute considerably to a highly dynamic perception of the respective river regimes.

Pleistocene river systems, in most cases, differed significantly from their current state (Vandenberghe 2001; Vandenberghe and Woo 2002). Sedimentological and geomorphological studies show that they are generally characterized by intense lateral channel movement, wide and extensive floodplains and terraces, and reduced sediment runoff and water load, or by linear, meandering, and constrained channels incising with small width-depth ratios, creating floodplains of rather limited extent (*e.g.*, Vandenberghe 2008). River behavior in Pleistocene settings, like today, is therefore expected to oscillate between “braided” and “meandering” configurations (Vandenberghe 2001, 2008; Vandenberghe and Woo 2002; Sambrook Smith *et al.* 2006; Wohl 2007; Baker 2007).

The dual classification of Pleistocene rivers raises the question whether both types are a function of different climatic regimes, namely warm (temperate) or cold (periglacial) climatic conditions, within the glacial cycle (*cf*. Vandenberghe 1995, 2002, 2008; Mol *et al.* 2000; Vandenberghe and Maddy 2001; Sambrook Smith *et al.* 2006). Although there is a broad correlation between braided river types and colder (glacial) periods and meandering river types and warmer (interglacial) periods, on shorter timescales, local thresholds seem to play a decisive role, decoupling river characteristics from direct climatic forcing (Vandenberghe 1995, 2002; see also Dibiase 2013; Finnegan *et al.* 2013). River systems are best understood as complex systems with self-stabilizing properties, translating into individual and distinct fluvial profiles that can become partially independent of major climatic events (see, *e.g.*, Knox 1993). Nevertheless, Bridgland and Westaway (2008) have recently shown that the phenotypical disparity between braided and meandering river systems became accentuated after the onset of macroscale Milankovitch cycles in the Middle Pleistocene, leading to steeper valley gradients and thus more confined fluvial features in the wider landscape during temperate phases.

**Fig. 3:**
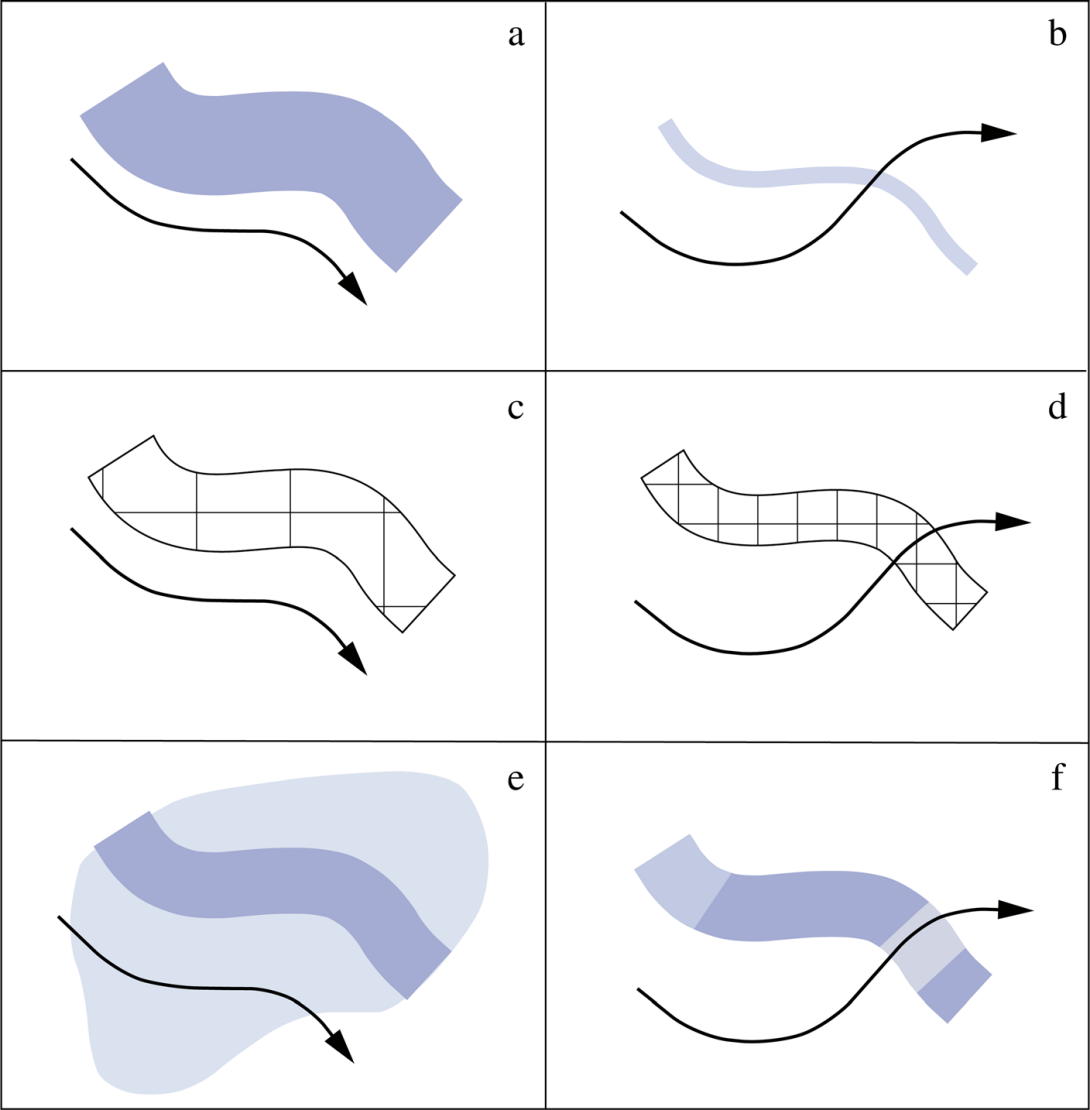
Natural river characteristics influence how people experience those fluvial regimes and therefore how to interact with them. The figure exemplifies possible links between key variables defining a river’s fluvial profile and human movement in space: **a** wide and deep river bed with high flow velocity, **b** narrow and shallow river bed with low flow velocity, **c** wide and deep river bed with nonhuman-carrying ice shield, **d** narrow and shallow river bed with human-carrying ice shield, **e** river expansion by transgression and periodic flooding events, and **f** river segmentation by partial shallowization. *Black arrows* indicate mobility patterns reflecting these profiles

Arguably, river exceptionality is strongly related to perceived and experienced fluvial dynamics, which contribute a great deal to the “personality” of a given river—to its individualized *persona* (*cf*. Fig. 2). Key elements of the underlying bioecological and spatiotemporal fingerprint are, for example, the presence or absence of periodic, large magnitude flooding events that severely impact the surrounding landscape (*cf*. Bonnamour 2000; Bonnamour *et al.* 2005) and seasonal or year-round river ice and snowmelt events influencing water load and flow velocity (*cf*. Vandenberghe 2001). Of considerable importance is also the alteration of fluvial conditions facilitating or impeding river crossing (Fig. 3). All these individual river parameters are linked to varying degrees to different geomorphological river types like sandurs, braided, meandering, anabranching, transitional, and deltaic rivers (*e.g.*, Vandenberghe and Woo 2002), which are characterized by different channel types and erosional energy loads (Smithson *et al.* 2002; Vandenberghe 2008). They have radically different phenomenological qualities and effectively stretch a continuum between permeable entities and natural barriers.

### Cultural Conceptualizations of Fluviality

Using the example of the Saône River in eastern France, Bonnamour *et al.* (2005) have recently shown the potential effects of these highly dynamic fluvial conditions on human presence and the ways people experience and understand them. In fact, rivers and “moving water” in particular seem to be the focal targets of sociocultural conceptualizations (*e.g.*, Strang 2004, 2008; Tóth 2006; Edgeworth 2011; Wilcock *et al.* 2013). Fluidity in general provides a vast imaginative potential to carry cultural meanings and is a powerful “device” to convey metaphors (Douglas 1973; Lakoff and Johnson 1980; Illich 2012; Strang 2004, p. 61). As a consequence, fluvial features are often incorporated into mythological narratives (Fig. 2), mirroring the fact that special places typically possess extraordinary or even unique natural characteristics (Bradley 2000; Kelly 2003, p. 45).

Periodic shifts in flow qualities and thawing are a good example in this respect. These microscale river transformations are ethnographically, in many cases, associated with huge feasting activities and “rites of passage”: the Native Americans of the North-west Coast, for example, coordinate gift-giving ceremonies with riverine fluctuations (*e.g.*, Pritzker 1998; Waldman 2006; Johansen and Pritzker 2008, 1048ff.; see also Strang 2004, 2008). Issues of identity are similarly negotiated in relation to fluvial regimes (Hiatt *et al.* 1968; Smith 1990; Strang 1997; Lee and Daly 1999). The Akulmiut of western Alaska, for example, consider themselves as “people between Yukon and Kuskokwim” (Andrews 1994), while the Khwe of southern Africa determine their location by referring to “this” or “the other side” of the Kavango River (Brenzinger 2008). In the case of the Cholanaickan of southern India, rivers even serve as boundaries that separate different regional groups and thus spatially construct and enforce social identities (Bhanu 1992).

Altogether, sociocultural conceptualization tends to target spatial features which evoke both strong affordances and unique experiences. The attribution of spatial meaning and significance further fosters the witnessed prominence and focality, resulting in an irreversible integration of these features into the sociocultural realm. Both natural and cultural archives, therefore, demonstrate that there are many reasons to pay special attention to large river systems in this respect. The role of Pleistocene rivers is thus largely dependent on both their formal and physiographic properties as well as on their sociocultural bearings. Both domains are truly entangled and influence each other in various ways. From a methodological standpoint, it follows that each river has to be treated as a unique entity with a particular personality. Its individual river profile has first to be determined geoarchaeologically (*e.g.*, Antoine *et al.* 2003), and then to be compared to the archaeologically inferred “sociocultural handling” of the river system. Only the relationship between the two archives can reveal the organizational logic that governs the ecocultural system that both human societies and river systems are part of. This also means that such ecocultural systems are always vulnerable to change and transformation, since already smaller reorganizations in the sociocultural sphere can lead to larger scale reorganizations of the entire ecocultural system. From this perspective, it should be extremely instructive to analyze the relationship between natural river characteristics and sociocultural constellations throughout the Late Pleistocene in order to detect larger ecocultural transformations through time, but also to attest important continuities. In what follows, we take stock of already published data from the Central and Western European Upper Paleolithic and discuss it in the context of changing river profiles, climates, and an accordingly shifting matrix of spatial prominence, affordances, and heuristic values. Special attention will also be paid to the specific role of landscape knowledge emanating from specific sociocultural settings.

## Rivers, Conduits, and Social Boundaries in the Paleolithic

The idea, that large river systems coincide with social boundaries and thus structure the Paleolithic world on larger scales, has been extremely influential in the field’s history. Some of these ideas even had a deep impact on how scholars recognized the entire period. Most famously, Hallam L. Movius claimed in the middle of the last century the existence of a demarcation line dividing the Lower Paleolithic into two hemispheres, one with bifaces and the other lacking them (Movius 1949). He identified the “Movius line” as a robust spatiotemporal feature in Central Europe thought to be largely identical with the course of the River Rhine. For a long time, scholars therefore considered the famous biface of Hochdahl as the only handaxe occurrence east of the river and beyond the Movius line (Andree 1939, 569ff.; Bosinski 2008, p. 113). Another famous historical example is the case of the Nile Valley seemingly forming the eastern margin of the Aterian world within the greater North African Middle Stone Age complex (Caton-Thompson 1946; Marks 1975, p. 440; Debénath 1986, p. 25; Kleindienst 2000, 2001; Hublin and McPherron 2012; Scerri 2013).

For both cases, however, it has never been demonstrated that the observed pattern is directly related to the river regimes in question. Hence, it remains unclear whether the respective distribution patterns are truly a result of the spatial significance of these river systems, or rather a function of research intensity and/or general landscape ecological structuring that is largely contingent on fluvial features. Additionally, influential ideas such as clear-cut river boundaries frequently foster double standards and therefore impact the interpretation of archaeological patterning *a priori*. The following discussion attempts to avoid these pitfalls and to link the observed spatial patterns with ecoclimatic variables that influence each river’s personality. Several case studies from the Central and Western European Upper Paleolithic serve to illustrate the unique character of those eco-fluvio-cultural systems and their stability and transformation through time.

### Early Upper Paleolithic Colonization of Eurasia

**Fig. 4:**
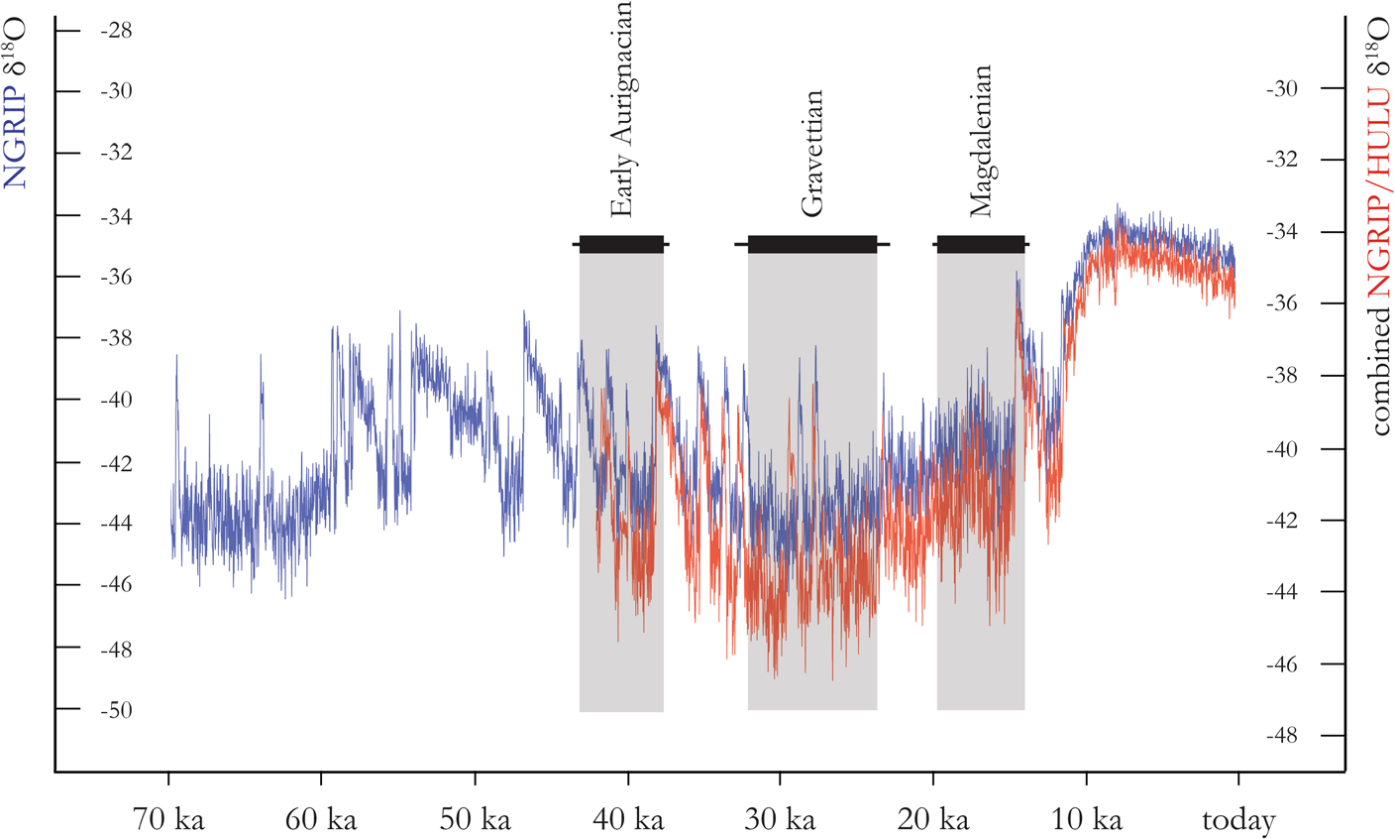
Chronoclimatic position of the discussed case studies. NGRIP (*blue*) and NGRIP/HULU (*red*) curves are used as climate proxy (Weninger and Jöris 2008)

Scholars generally agree that the initial colonization of the European continent by *Homo sapiens* is paralleled by Early Upper Paleolithic signatures (Conard and Bolus 2003; Mellars 2006b; Richter *et al.* 2012; Tsanova *et al.* 2012; Stoneking and Havarti 2013; Hublin 2012, 2014; Benazzi *et al.* 2015). Within this framework, fully Upper Paleolithic blade and bladelet industries of the Proto- and Early Aurignacian are considered to represent the first successful and enduring settlement of Central and Western Europe that affected almost the entire continent (Mellars 2006b; Tsanova *et al.* 2012; Hublin 2014). From this perspective, the dispersal of anatomically modern humans (AMH) into Eurasia provides a good example of groups encountering new and hitherto unknown landscapes (*sensu* Rockman 2003) with relatively cold climates (*e.g.*, Nigst *et al.* 2014; Riehl *et al.* 2014; Fig. 4).

**Fig. 5:**
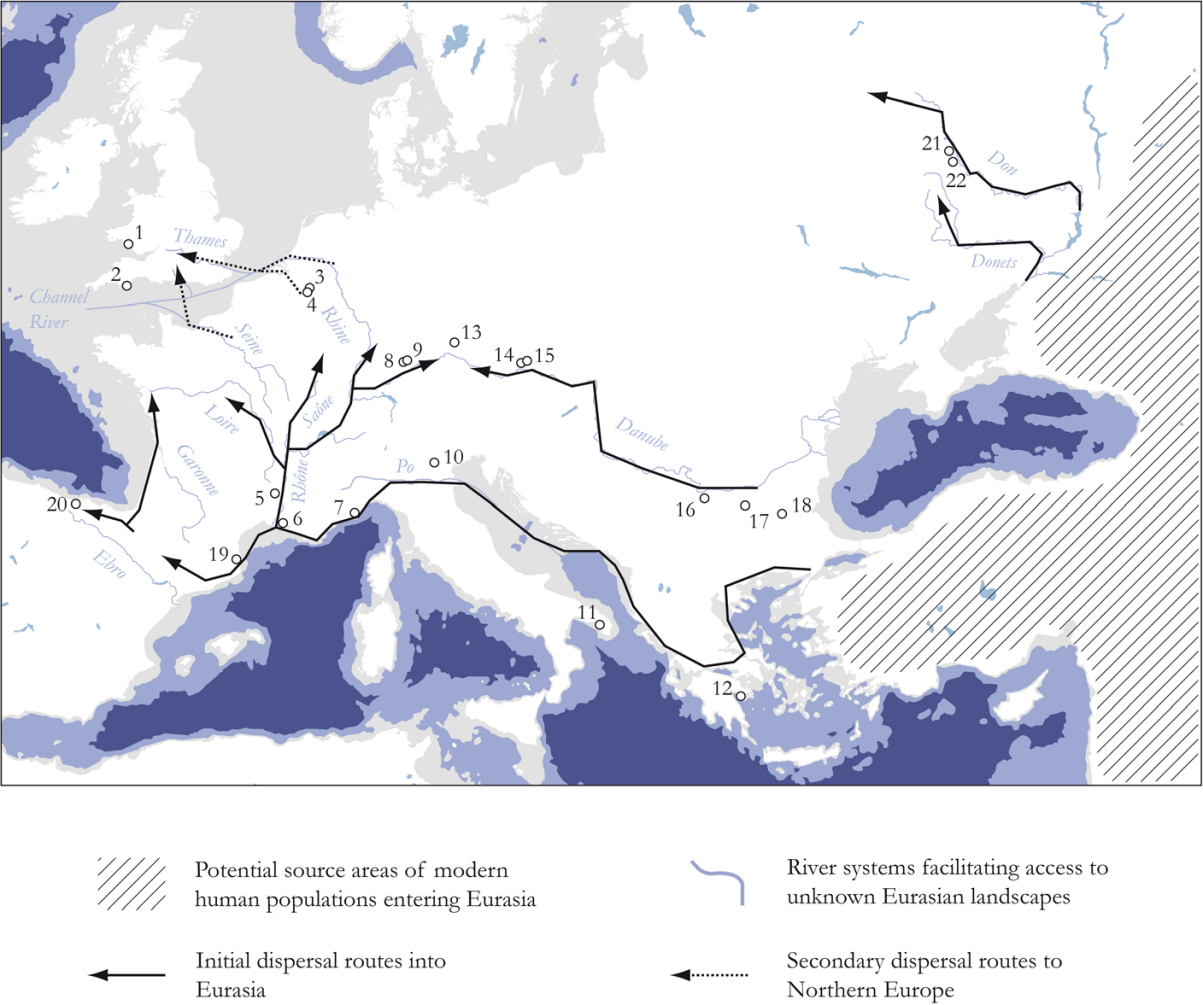
Schematic map of likely colonization routes to Central and Western Europe during the Early Upper Paleolithic. Large river valleys and the Mediterranean coastline act as primary migration corridors channeling the movement of AMHs. Key sites supporting this view are mapped (for age determinations, see S1, Electronic Supplementary Materials). It should be noted that this scenario opens up the possibility of a bidirectional dispersal to Europe, and to southern Germany in particular: *1* Goat’s Hole (Paviland), *2* Kent’s Cavern, *3* Goyet, *4* Trou Magrite, *5* Grotte Chauvet, *6* La Salpêtrière, *7* Riparo Mochi, *8* Hohle Fels, *9* Geißenklösterle, *10* Grotta di Fumane, *11* Grotta di Cavallo, *12* Franchthi, *13* Keilberg, *14* Willendorf II, *15* Senftenberg, *16* Kozarnika, *17* Temnata, *18* Bacho Kiro, *19* L’Arbreda, *20* El Castillo, *21* Voronezh, *22* Kostenki

The earliest evidence of an Aurignacian presence in Europe has been documented along major drainage systems (Davies 2001; Conard and Bolus 2003; Mellars 2006b; Anikovich *et al.* 2007; Dinnis 2012a) and coastal areas (Mellars 2006b, 2011; Moroni *et al.* 2013). Both spatial data and the internal chronology of the Aurignacian technocultural package therefore indicate the pivotal role of large river regimes in facilitating access to the vast and heterogeneous landscapes of the European continent (Hussain and Floss 2014; Higham *et al.* 2014; Fig. 5; compare S1, Electronic Supplementary Materials).

The Danube River in particular holds a key position in *structuring* population movement and sociocultural exchange in an east–west direction (Conard 2000, p. 351; Conard and Bolus 2003, 2008; Floss 2003a, 2009a; Bolus 2009). Recent radiocarbon dates from the key Swabian Jura sites of Hohle Fels and Geißenklösterle in the Ach valley, a small tributary of the Upper Danube, support this view and place the earliest occupation in the region even before Heinrich event IV (Conard 2009a; Higham *et al.* 2012, 2014). Dates between 40 and 42 ka cal. BP for a well-established Early Aurignacian presence in the Upper Danube suggest that AMH groups very rapidly entered the gates of Europe *via* this large river valley (Conard and Bolus 2003; Bolus 2009). The age determinations are supported by several other Early Aurignacian sites from the region that yielded calibrated ^14^C dates between 38 and 40 ka BP (Conard 2003, 2006; Nigst 2006; Jöris *et al.* 2010; Kind *et al.* 2015). Moreover, these sites share a regionally distinct “cultural heritage” represented by a unique tradition of both ivory figurines and personal ornaments (Hahn 1993; Conard and Floss 2000, 2010; Vanhaeren and d’Errico 2006; Floss 2007, 2009b; Conard 2007, 2009a, b; Conard *et al.* 2009; Porr 2010; Wolf 2015; Conard *et al.* 2015).

We would argue that the emergence of a regionally distinct tradition of figurine and ornament making already signals the end of a true “pioneer phase” in the initial incursion into the European mainland. From this perspective, the very early and therefore pioneer Aurignacian assemblages reflecting an initial advance into largely unknown landscapes are expected to be even more rudimentary and ephemeral in this respect (*cf*. Davies 2001, 2007). Recent evidence from Willendorf II in Lower Austria is in strong agreement with this model and demonstrates the presence of Aurignacian technologies without personal ornaments and figurines along the Danube already before 43 ka cal. BP (Nigst *et al.* 2014).

Combined with a raw material procurement pattern largely reproducing the spatial vector of the Danube (Floss and Kieselbach 2004, p. 76; Jöris *et al.* 2010), these findings point to a highly interconnected “taskscape” along the large river system. Hence, both chronometric evidence and material culture characteristics argue in favor of the Danube’s role as a spatial corridor for human dispersal and an axis of sociocultural exchange in the Early Upper Paleolithic (*e.g.*, Floss 2003a, b).

Being a typical Alpine river regime, the Pleistocene Danube can broadly be characterized as a wide, multichannel, and high-energy stream with frequent riverine islands (Ward *et al.* 2002; Lóczy 2007; Miklós and Neppel 2010, p. 108). As a consequence of the Later Pleistocene Rhine incision withdrawing considerable amounts of water from the Upper Danube hydrological system, flow velocities and water load decreased progressively during the course of the last glacial (Lóczy 2007; Miklós and Neppel 2010). Nevertheless, biodiversity within the extensive river catchment was relatively high due to a myriad of different ecological niches hosting various floral and faunal communities (Ward *et al.* 2002; Lóczy 2007; Wang *et al.* 2012).

Environmental and climatic data shows that this general fluvial profile of the Danube became accentuated at the end of MIS 3, resulting in a readily accessible, but highly dynamic and sensitive river regime in a cold steppe-type environment with only a few boreal trees along the river valley (*cf*. Weninger and Jöris 2008; Nigst *et al.* 2014; Riehl *et al.* 2014; compare Fig. 4). In this setting, the river becomes both physically and ecologically prominent, guarantees landscape connectivity, and establishes steep biodiversity gradients to the surrounding environments. Along with the fundamental accessibility of a braided Danube, this fluvial profile would have *afforded* people to exploit the river as a bridge between different landscape units. Furthermore, the Danube is the only east–west trending river in this part of the European continent and thus a naturally exceptional physiographic feature in this respect alone.

A similar role as an Early Aurignacian expansion route is hypothesized for the Don River system near the Black Sea (Anikovich *et al.* 2007; Fig. 5). Bataille (2013, 76ff.) has recently reinforced this model and extended it to the whole region, arguing for the important role of the Dniester, the Dnepr, and the Don in accessing new land in Eastern Europe during this period. In a systematic survey of Late Aurignacian occurrences on the British Isles, Dinnis (2008, 2009, 2012a) has proposed an analogous perspective for the now submerged Channel River system in explaining human dispersal into Northern Europe (*cf*. Fig. 5). The argument is mainly based on the striking western distribution of Late Aurignacian sites in Britain, which cannot be explained by differential find preservation or lack of research alone (Jacobi 2007; Pettitt 2008; Flas 2009; Dinnis 2012a, b). In the past, scholars have proposed that these Aurignacian groups came from northwestern France because of its close spatial proximity and a presumed environmental similarity between the two regions (Jacobi 1999; Pettitt 2008). The distinct spatial patterning of Paviland burin technologies, however, now clearly favors an eastern source area comprised of today’s Belgium and parts of northeastern France (Flas *et al.* 2006; Dinnis 2008, 2012a). It is thus very likely that the Pleistocene Channel River network *directed* AMH movement to the British mainland (Pettitt 2008; Dinnis 2008, 2009, 2012a, b). This model is particularly appealing because the Late Pleistocene Channel River was directly linked to the Seine and the Rhine river system at this time (Antoine *et al.* 2003; Lericolais *et al.* 2003; Toucanne *et al.* 2010), offering a high degree of landscape interconnectivity that could easily be exploited by mobile foraging groups coming from the heart of Europe (*cf*. Fig. 5). Moreover, the river occupied an extensive area in the landscape with multiple migratory channels, minor tributaries, and a massive floodplain, including swamp and marsh environments (*e.g.*, Hijma *et al.* 2012). Apart from being extremely attractive for animals in general, and migratory fauna in particular (Dinnis 2009, 2012a), this spatial configuration brings forth an accessible and affording fluvial feature, testified in a high degree of connectivity in the archaeological patterning.

To conclude, it appears that Aurignacian site occurrence at the European scale is strongly correlated with the presence of large river systems in the landscape (Otte 1979; Davies 2001; Van Andel *et al.* 2003). Individual prominent, accessible, and strongly accommodating river systems considerably shape the sociocultural geography of the Aurignacian record. These rivers clearly serve as key features ensuring connectivity between various occupational areas. We would argue that the role of these river systems as communication corridors and mobility conduits is largely a result of both their ecophysiographic characteristics and the *unique social context of dispersal and migration*. To begin with, if we accept the two-phase dispersal model of Aurignacian technologies, with a pioneer and a consolidated phase reflected in different sociocultural signatures (Davies 2001, 2007), the *conditions of landscape knowledge and learning* deserve special attention (*e.g.*, Golledge 2003; Kelly 2003). It is reasonable to assume that AMH groups entering Europe experienced largely unknown and unfamiliar landscapes and had very limited ecological knowledge on which spatial behavior, food acquisition, and sociocultural topology could be based. Large rivers are then very likely to play a key role because they are reliable spatial heuristics for orientation and the exploitation of the wider landscape. The visual prominence and physical accessibility of mostly braided river systems in the Early Aurignacian cold climatic phase further enhances their affordance structure. In the wider ecocultural system of Aurignacian times, these rivers can therefore be characterized as anchor points that organize spatial cognition and performance. Golledge (1978) already pointed out that such anchoring features are designated to hierarchically structure the local environment around them and serve as a “cheap” and effective means of landscape use and way-finding in particular (see also Couclelis *et al.* 1987). This emerging property would thus underscore the integral role of large rivers in the context of “fast and frugal” decision-making during human expansion and colonization.

Gärling *et al.* (1984) have shown that the “environmental legibility” that contributes to a spatial feature’s experienced quality varies according to the conditions of landscape knowledge and can thus be discussed in relation to three main stages: exploratory, adaptive, and abstract. The exploratory stage, broadly equitable to dispersal and migration scenarios, is generally characterized by the dominance of visual experience in orientation. Human groups therefore use *concrete* spatial representations mainly related to focal physiographic features (Golledge 2003, p. 36). Moreover, there seems to be a positive relationship between “landscape unfamiliarity” and the focality of spatial features that impact the mobility of social groups (Kelly 2003, p. 48). In the adaptive and abstract stages, on the other hand, sociocultural meaning becomes increasingly separated from physiographic properties. This general matrix of spatial performance might explain why large river systems play such an important role in the successive settlement of the European continent, both as *directing* and *channeling* spatial features, and offers a parsimonious blueprint for interpreting the spatiality of the Aurignacian record (compare Fig. 5).

Apart from large river systems, coastlines have recently also become an important candidate for channeling AMH dispersal into Europe (Mellars 2006a, b; Richter *et al.* 2012; Tsanova *et al.* 2012; Hublin 2014; see also Bulbeck 2007). This model is not in general disagreement with the claim that rivers spatially organize the accessing of new land during the Early Upper Paleolithic. A whole range of arguments supporting the crucial role of fluvial features in the colonization process can also account for coastlines. Although being effective barriers in most cases, they can also unfold powerful channeling properties (*e.g.*, Stringer 2000) that can supplement or even substitute for river conduits.

Since new ^14^C dates, obtained from marine mollusks, have become available from Aurignacian coastal sites in Greece, Italy, and southern France, the idea that AMH groups might have accessed Europe *via* two routes, along both the Danube corridor and the Mediterranean coast, has become very persuasive (*e.g.*, Douka *et al.* 2012). This emerging pattern is further supported by a recent reassessment of the anthropological status of the teeth remains from Grotta di Cavallo as *H. sapiens* (Benazzi *et al.* 2011; Bailey *et al.* 2014). They place the arrival of AMH groups at the Mediterranean shores *before* 40 ka cal. BP (Higham *et al.* 2014) and indicate a more complex archaeological signature for this second dispersal modality (Moroni *et al.* 2013; Hublin 2014). Together with the early dates from Franchthi Cave in Greece (Douka *et al.* 2011), Riparo Mochi in Italy (Douka *et al.* 2012; Higham *et al.* 2014), and the early Protoaurignacian sites in the Rhône delta, dating around 40 ka cal. BP (Douka, personal communication), the evidence clearly speaks in favor of a swift dispersal scenario along the coast resulting in the early penetration of southern France and northeastern Spain (compare S1, Electronic Supplementary Materials).

These dates also reintroduce the possibility of a bidirectional colonization of Central Europe in Aurignacian times, taking into account source populations along the Mediterranean coast of southern France that followed the Rhône-Saône axis up-river (compare Fig. 5). Although direct ^14^C dates are still lacking for the Aurignacian occupation at Germolles in Burgundy (Floss 1997, 2000a, b, 2001), there is nevertheless a clear indication that eastern France and southern Germany were inextricably connected in this period. A small collection of artifacts, including one diagnostic Aurignacian *pièce carinée* made of “Bohnerzjaspis” and several blades made of “Jurahornstein” (Jurassic chert), from the Grotte de la Verpillière I, most likely links Burgundy and the Breisgau region, where these raw materials can be sourced. From this perspective, it emerges that large river systems and coastlines both serve as *interconnected spatial conduits* to facilitate access to Europe’s “interior” landscapes during the Early Upper Paleolithic.

### Research History and Spatial Limits of Archaeological Entities Throughout the European Upper Paleolithic

**Fig. 6:**
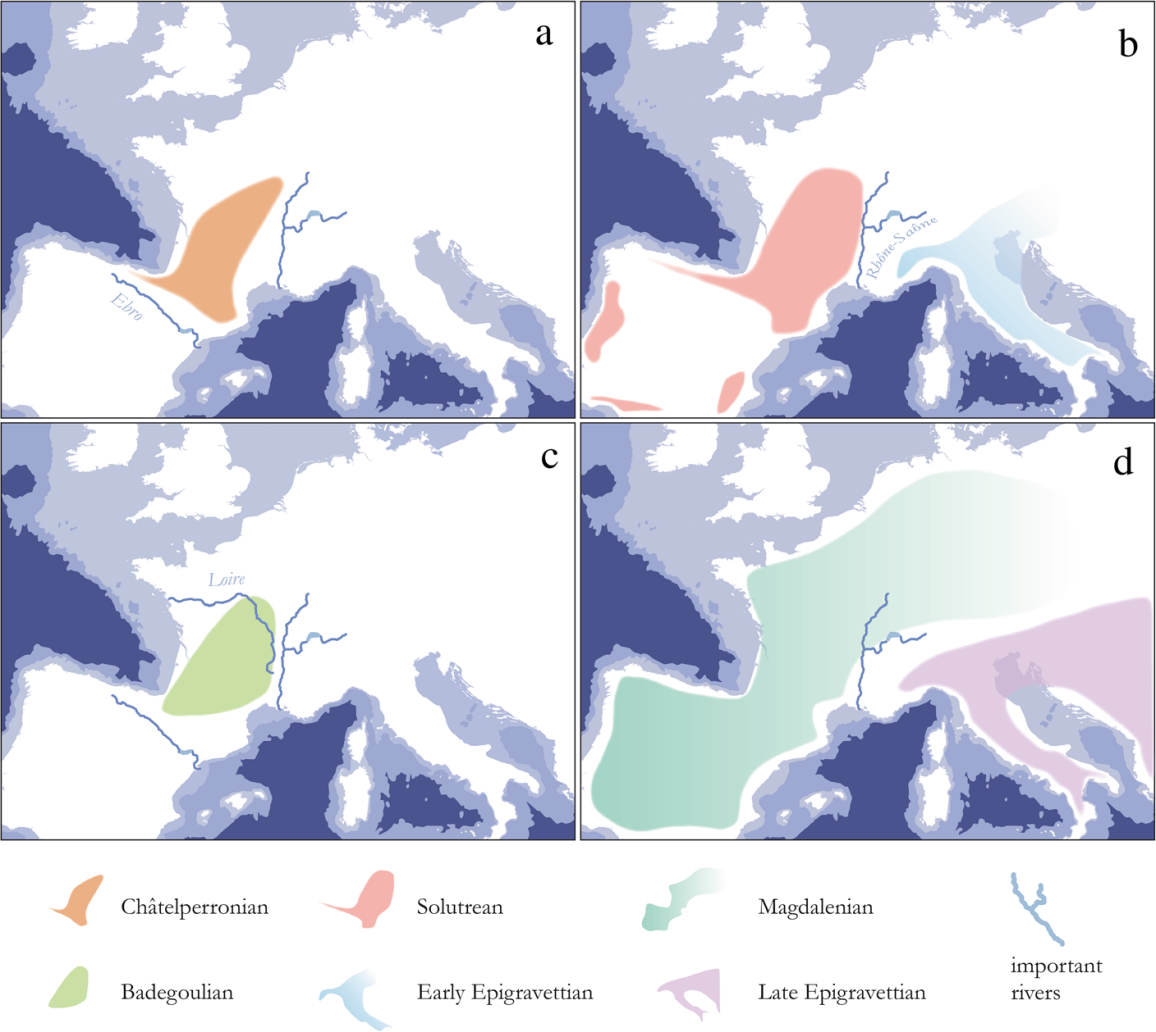
Spatial extension of initial and full Upper Paleolithic entities in relation to important river systems mentioned in the text: **a** Châtelperronian of southwestern France lying in-between the Ebro formation and the Rhône-Saône river system (Connet 2002; Pelegrin and Soressi 2007); **b** Solutrean and Early Epigravettian divided by the course of the Rhône (Mussi 2002; Banks *et al.* 2008); **c** Badegoulian framed by the Ebro, Rhône, Saône, and Loire river systems (Banks *et al.* 2011); and **d** Magdalenian and Late Epigravettian separated by the Rhône River (Mussi 2002). Spatial distributions are approximate and claim accuracy only in relation to the critical river courses

Large river systems are influential ecocultural features that considerably organize and constrain human mobility. Depending on both their natural profile and the sociocultural context in which they are encountered, experienced, and conceptualized, their role can change significantly. Whereas dispersal and migration scenarios favor a role of rivers as corridors, conduits, or trajectories, spatial consolidation processes can broadly be paralleled with the adaptive and abstract stage of “environmental legibility” of Gärling *et al.* (1984), which, in turn, is expected to result in a variety of different spatial effects. This is particularly plausible if the entities in question have strong historical roots in archaeological units preceding them in time, because it can be assumed that ecological and environmental knowledge is well available then. Several examples from the European Upper Paleolithic illustrate this variety (*e.g.*, Floss 2000a; Hussain and Floss 2014; Fig. 6) while showing that research history often heavily biases the spatial record. Issues of research intensity and focus are essentially a problem in macroscale approaches. Nevertheless, macroscale patterning remains one of the most important sources for exploring the role of river systems in different Upper Paleolithic settings, simply because rivers often structure *entire* ecocultural landscapes. In what follows, we outline a few examples that highlight the macroscale impact of large river systems in constraining the spatial extend of greater technotypological entities.

To begin with, the transitional or initial Upper Paleolithic Châtelperronian of southwestern France and northeastern Spain (Pelegrin and Soressi 2007; Soressi and Roussel 2014; Roussel 2011, 2014) spans an area between two large river systems that seem to limit its spatial extent: the Rhône River to the east and the Ebro River valley to the south (*cf*. Floss 2000a, p. 121; Fig. 6a). In this case, it seems that no ecophysiographic rationale can be introduced to explain this pattern. On the contrary, two arguments support an anchoring of the Châtelperronian’s spatial limitation by river courses in sociocultural tradition: first, the preceding *Moustérien de Tradition Acheuléene* (MTA), in which several scholars identify the technological roots of the Châtelperronian (Pelegrin and Soressi 2007; Roussel 2013), is also restricted to the west of the Rhône Valley, while the Central and Eastern European *Keilmessergruppen* (KMG) occupy large parts east of the river (Richter 2009; Ruebens 2013). Moreover, the MTA also stops before the Ebro escarpment (Ruebens 2013). This, therefore, raises the possibility that the structure of human geography in Late Middle Paleolithic times lays also the foundation for an ecocultural conception of “worldly limits” in the subsequent Châtelperronian. Such developments might reflect a general tendency toward regional diversification from around 60 ka cal. BP onward (*cf*. Langley *et al.* 2008), which can be interpreted as a process of “spatial consolidation in familiar landscapes,” where the coordination of different coexisting groups and sociocultural units in space plays an important role.

A similar role as a dissecting spatial feature can also be ascribed to the Rhône-Saône river system during the onset of the Last Glacial Maximum (Fig. 6b). The large fluvio-physiographic feature separates the Solutrean from the Early Epigravettien (Floss 2000a, 2005, 112f.; Banks *et al.* 2008; Hussain and Floss 2014). Banks *et al.* (2008) have suggested that this spatial pattern is largely a function of crucially different ecological conditions east and west of the Rhône River, leading to completely different technocultural adaptations. Nevertheless, it is still reasonable to argue that periglacial permafrost conditions in the Rhône Valley, with successive freezing and flooding events (Banks *et al.* 2008), hampered communication between human groups east and west of its banks. Along with the impact of nearby mountain ranges, glaciers, and associated glacial lakes, this would have resulted in a spatial configuration limiting human movement and communication across the river system. This setting, therefore, would have created strong affordances for avoiding the area and favors sociocultural frontier notions. The succeeding Badegoulian technocultural entity conserves this eastern margin, coinciding with the course of the Rhône-Saône river regime (Floss 2000a; Banks *et al.* 2011). Moreover, its source area is well defined by major river systems and coastlines in the west and south (Fig. 6c).

In the Late Upper Paleolithic, however, rivers seem to lose most of their structuring role altogether, the Rhône-Saône river frontier being a mere “historical relict,” at best (Mussi 2002; Fig. 6d). The duality between the Magdalenian and Late Epigravettian technotypological substrates may even be the result of divergent research traditions with different classification systems: the Bordian scheme in the case of Central and Western Europe and the Laplace scheme in Italy and large parts of Eastern Europe (Mussi 2002; Maier 2012a).

The stability of some overarching patterns in the face of ever-changing climate regimes and ecological conditions, such as the Rhône’s character as an important landmark through time, suggests that landscape knowledge and spatial conceptualizations are an important dimension of site distribution during the Late Pleistocene. At the same time, shifting relationships between fluvial features and archaeological archives demonstrate that each ecocultural system is unique, integrating and relating its components differently. In the case of the Rhône, however, generally high discharge values and flow velocities might argue in favor of a river *persona* that really marks a significant spatial threshold in the wider landscape (*cf*. Pardé 1925).

### Middle Upper Paleolithic Settlement Consolidation Along the Garonne Valley

**Fig. 7:**
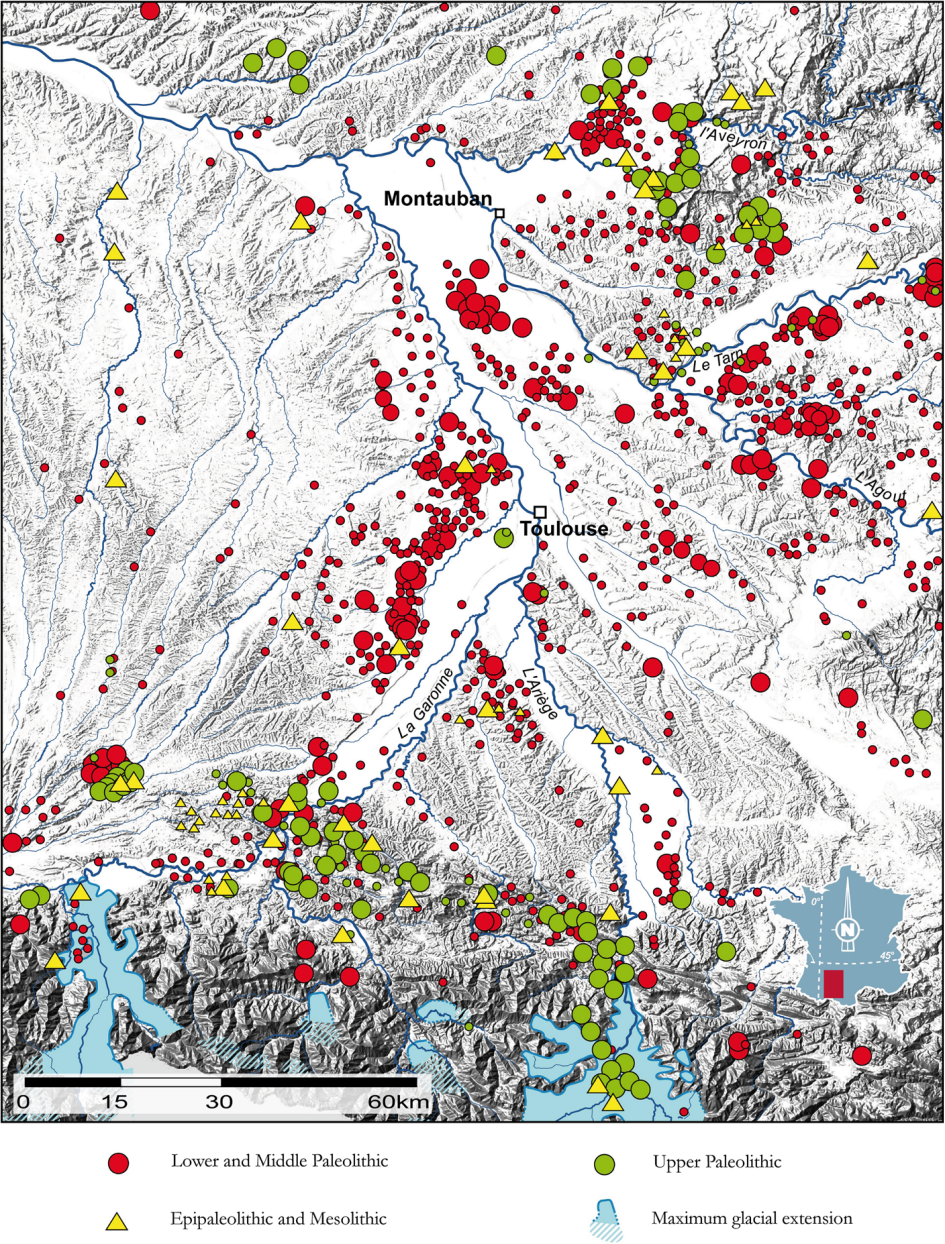
Recorded archaeological occurrences in the Upper Garonne valley from the Pleistocene to the Holocene transition. Upper Paleolithic sites cluster on the edges of the mountainous areas peripheral to the main valley, while the other time slices are also represented in the valley itself. Geomorphology and preservation issues cannot be invoked to explain this pattern because geological strata of the Upper Paleolithic are present but sterile in the valley, and Middle Paleolithic and Epipaleolithic site distribution demonstrates that research history is not the explaining factor. *Dot-size* indicates the frequency of occurrences. With courtesy of Marc Jarry and Laurent Bruxelles (INRAP, University of Toulouse)

As one of the largest river systems in Western Europe, the Garonne provides a good example of spatial segregation on a regional scale during the Upper Paleolithic in general and during the Middle Upper Paleolithic in particular. Recent interdisciplinary investigations along the river and in its catchment area have demonstrated the river’s special character in Upper Paleolithic times (Bruxelles and Jarry 2011, 2012; Jarry and Bruxelles 2012). Landscape archaeological research and high-resolution data on geomorphology and environmental conditions enable the diachronic reconstruction of the occupational history in the Garonne River catchment (Fig. 7). In contrast to other represented time slices, traces of Upper Paleolithic presence in the valley itself are almost completely lacking. Consequently, Bruxelles and Jarry (2011, 2012: Fig. 3) have argued that the Garonne Valley must have constituted an area of spatial avoidance during Upper Paleolithic times. Their comprehensive and region-wide study leaves no doubt that this finding is epistemologically robust. Firstly, the lack of evidence cannot be explained only by preservation issues or the state of research in the region and must therefore be regarded as true “evidence of absence.” Geomorphological results demonstrate the presence of the stratigraphic unit which corresponds to the Upper Paleolithic time slice in the area, thus excluding postdepositional processes as an explanation for the lack of site occurrences. Secondly, research history cannot account for the respective spatial patterning because the study area yields a firm number of both preceding Middle Paleolithic sites and succeeding Epipaleolithic/Mesolithic sites close to the river (Fig. 7). The Middle Paleolithic occupation in particular seems to be centered on the river basin itself, whereas Upper Paleolithic sites group adjacent to the main valley and occupy higher elevations only.

Bruxelles and Jarry (2012, p. 81) have suggested that this shift in spatial organization can be paralleled with a slight climatic deterioration at this time which, in turn, would explain the rupture in human river engagement. Such a climatic degradation, however, can also—and probably more convincingly—be interpreted as an experience and perception-shifting factor that considerably limited the accessibility and appeal of the river system in the wider landscape. The new ecophysiographic configuration of the fluvial feature within the overall spatial context thus would have afforded people to avoid the immediate vicinity and, hence, would have favored a “negative image” in the realm of sociocultural meaning. In this point, we are essentially following Tim Ingold, who persuasively argued that “it is important to note that no feature of the landscape is, of itself, a boundary. It can only become a boundary, or the indicator of a boundary, in relation to the activities of the people or animals for whom it is recognized or experienced as such” (Ingold 2000, 162ff.).

**Fig. 8:**
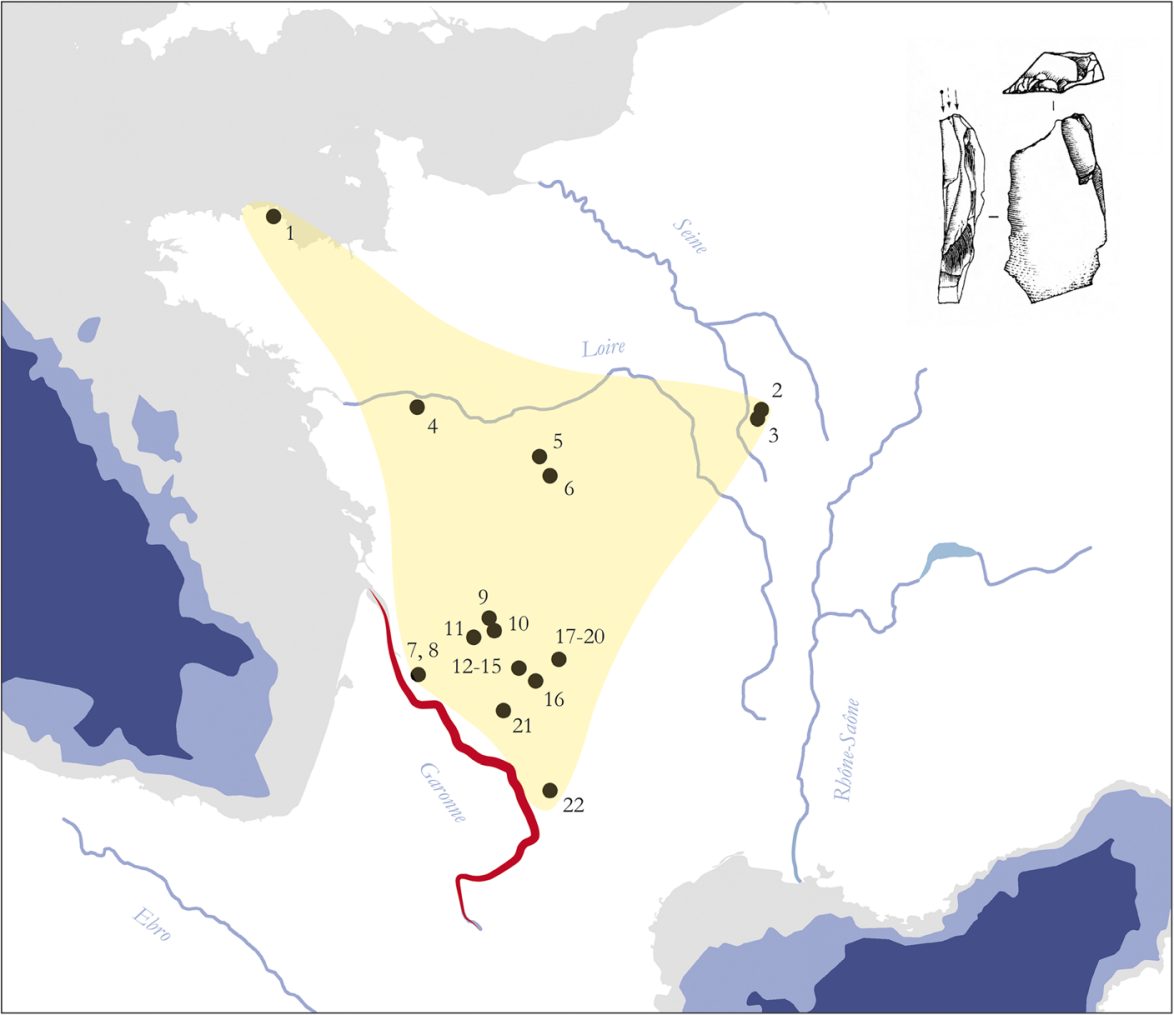
Distribution of Middle Upper Paleolithic sites yielding Raysse burin technology. The Garonne valley serves as well-defined southern frontier for the presence of Raysse burins in the Gravettian, separating the Raysse facies from the rest of the Gravettian world (modified after Klaric 2008: Fig. 1): *1* Plasenn’al Lomm, *2* Arcy-sur-Cure, Grotte du Trilobite, *3* Arcy-sur-Cure, Grotte du Renne, *4* La Martinière, *5* Les Roches de Pouligny-Saint Pierre, *6* La Picardie, *7* Les Artigaux, *8* Abri Lespaux, *9* Le Fourneau du Diable, *10* Les Jambes, *11* Solvieux, *12* Abri Pataud, *13* La Roque Saint-Christophe, *14* Masnègre ou Masnaigre, *15* La Rochette, *16* Le Flageolet I, *17* Les Morts, *18* Pré-Auberts, *19* Bassaler Nord, *20* Le Raysse, *21* Le Roc de Gavaudun, *22* Les Battuts. Note that the total distribution of the Gravettian exceeds the map by far

The sociocultural dimension of the Garonne River system is well reflected in the wider Middle Upper Paleolithic record of the region, supporting the outlined interpretation above. The almost depopulated Garonne River catchment coincides with one of the widely discussed margins of the Gravettian world in southwestern France (*e.g.*, Klaric 2008) and, therefore, intersects and disconnects the Gravettian taskscape at this particular point (Fig. 8). From this perspective, the Garonne River regime can be regarded as a spatial feature that *structures* the technological diversity of the Western European Gravettian. In particular, it clearly limits the southern extent of the Raysse burin distribution, which is considered to be a technological marker of social identity in this period (Djindjian *et al.* 1999, p. 185; Klaric *et al.* 2009).

**Fig. 9:**
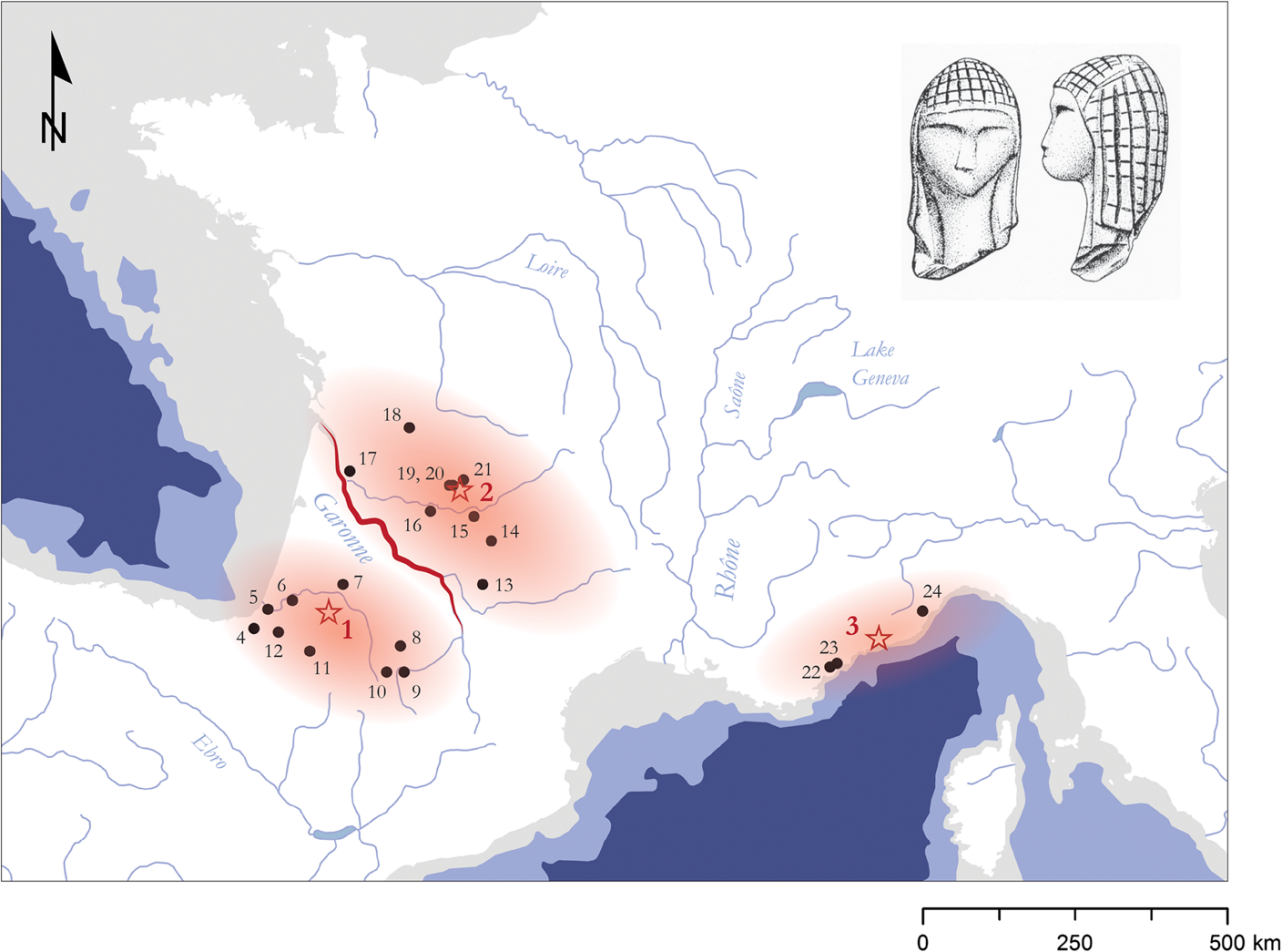
Regional Gravettian groups that center around the female figurine sites of Brassempouy, Laussel, and Balzi Rossi. The Garonne River can be considered the main feature that separates two of these units and organizes their spatial layout (redrawn from Simonet 2012: Fig. 86): *1* Brassempouy, *2* Laussel, *3* Balzi Rossi, *4* Lezia, *5* Le Prissé, *6* Tercis, *7* Pujo-le-Plan, *8* Lespugue, *9* Tarté, *10* Gargas, *11* Gatzarria, *12* Isturitz, *13* Les Battuts, *14* Pech-Merle, *15* Cougnac, *16* Cussac, *17* Pair-non-Pair, *18* Grotte du Vissage, *19* Cro-Magnon, *20* Pataud, *21* Tursac, *22* La Cabre, *23* Le Gratadis, *24* Arene Candide

Additional evidence for a structuring role of the Garonne River in the wider ecocultural space of the Gravettian derives from the nature of relationships between different key sites located in the neighborhood of the river system and the respective material culture archives they contain. Concerning the regional structure of the female figurine sites of the Gravettian, Simonet (2012) has recently argued that in southwestern France, two such core areas, separated by the Garonne fluvial system, can be distinguished. On the basis of raw material catchment areas and affinities in material culture composition, it can be demonstrated that a regional group centering around Brassempouy can be differentiated from a site group around Laussel (Simonet 2012, 85ff.; Fig. 9). It seems thus very clear that the Garonne forms a cultural frontier in Middle Upper Paleolithic times and therefore underpins the internal structure of Gravettian spatiality. It should be noted that this structuring property of the river is largely independent of zoogeographic patterns in the wider region (Stewart *et al.* 2003), therefore, further supporting a crucial sociocultural dimension of the attested frontier notion.

Today, the Garonne is a highly dynamic fluvial feature that regularly impacts its hinterland with middle to large magnitude floods and exhibits a powerful tidal bore (Chanson *et al.* 2011). Recent investigations have shown that large and rapid fluctuations in turbulent velocities and stresses during the tidal bore and flood flow are a striking characteristic of the river regime (Chanson *et al.* 2011; Simon *et al.* 2011). Whether this was already a property of the Late Pleistocene Garonne remains largely unclear (Chanson, personal communication). Nevertheless, the “restless” character of the river in general must have even become accentuated between 35 and 30 ka cal. BP, when the river profile was influenced by high amplitude climate oscillations (compare Fig. 4). This would have resulted in an amplified dynamism expressed by an ongoing alteration of braided and meandering river configurations. Combined with cold winds from the Atlantic (Bruxelles *et al.* 2010), this ecofluvio-physiographic setting underscores a sociocultural conceptualization of the river as mobility frontier.

Taken together, all these arguments indicate the important role of the Garonne River system in catalyzing spatiotemporal dynamics during the Middle Upper Paleolithic. The river’s role is closely linked to its natural characteristics, but also attests to the general framework of readily available landscape knowledge and the ongoing consolidation of AMH settlement in Europe after the colonization process in the proceeding Early Upper Paleolithic. From this perspective, the Garonne reflects regionalization and spatial diversification and, therefore, testifies to overarching trajectories of the ecocultural system of its time.

### Early to Late Upper Paleolithic Settlement Dynamics Along the Ebro River

The Ebro River system in northeastern Spain is the second largest river on the Iberian Peninsula and has long been of special importance for archaeologists and paleoanthropologists interested in the spatiotemporal dynamics of late Neanderthal demise and the emergence of AMH groups in this region (d’Errico *et al.* 1998; Delson and Havarti 2006; Zilhão 2009). In a seminal paper, d’Errico *et al.* (1998) have argued for the existence of an “Ebro frontier” separating late Neanderthal populations in southern Spain from the first AMH arrivals in southwestern France and northernmost Iberia (compare Zilhão 2000, 2009; Zilhão *et al.* 2010). Much attention has been paid to the apparently young ^14^C dates of Gorham’s Cave level IV in Gibraltar which seem to support this scenario (Finlayson *et al.* 2006, 2008; compare also Rodríguez-Vidal *et al.* 2014). Furthermore, a range of biogeographical arguments have been brought forward to demonstrate a refuge-like role of southern Iberia and Gibraltar, respectively (Finlayson and Carrión 2007; Jennings *et al.* 2011). In the face of considerable dating problems, however, it is unclear how much credibility these dates still bear (Jöris and Adler 2008; Jöris and Street 2008; Blockley *et al.* 2008; Pettitt 2008; Jöris *et al.* 2011; Maroto *et al.* 2012). Other issues touch upon the postulated favorable condition quality of the area south of the Ebro basin, which has recently been challenged by new climatic and biogeographic data indicating that southern Iberia was at times far from a refugiale zone (but see Balin *et al.* 2013). On the contrary, the critical time frame around Heinrich IV is characterized by a displacement of the 100-mm precipitation boundary to the north, resulting in an aridization of most of the area (Sepulchre *et al.* 2007; Jiménez-Espejo *et al.* 2007; Bradtmöller *et al.* 2012; Schmidt *et al.* 2012).

**Fig. 10:**
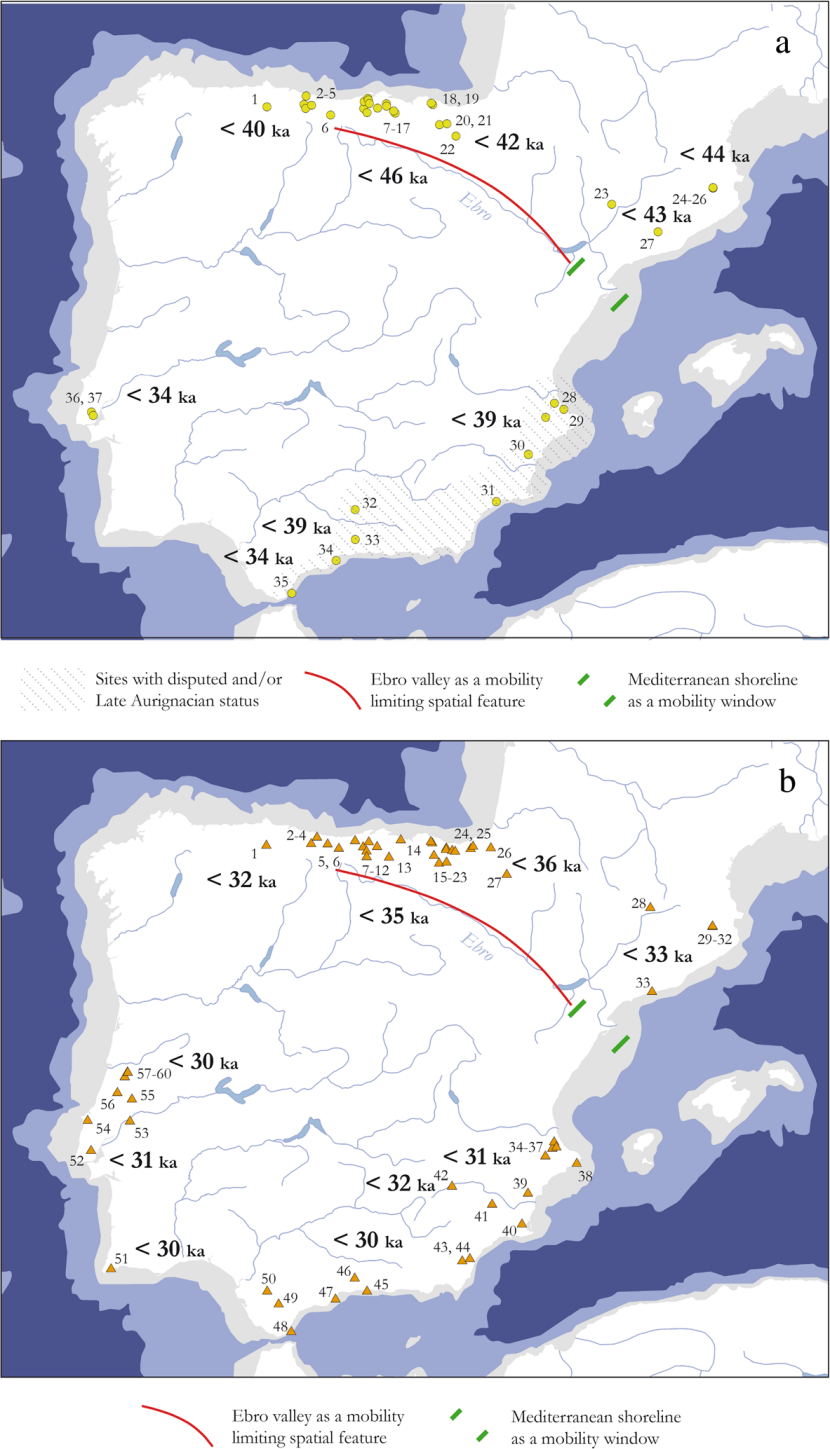
Early Upper Paleolithic and Gravettian site distribution on the Iberian Peninsula (modified after Schmidt *et al.* 2012). The spatiotemporal pattern shows that the Ebro is an important landmark for settlement organization. In the Early Upper Paleolithic (**a**), the Ebro valley is an important frontier hindering human movement. Only in the later or “evolved” Aurignacian, sites are recorded in the southern part of Iberia. This pattern testifies to the important role of the Mediterranean coastline as a powerful pull factor that channels movement to the south. Note, however, that the status of several sites in southern Iberia as Aurignacian is disputed. In the Gravettian (**b**), a comparable picture emerges. Sites in the north are still older than in the south, speaking in favor of a scenario in which southern Iberia was successfully settled only during the later phase of the Gravettian. Both maps show only cave sites. AMS dates are used to determine the spatiotemporal pattern (for age determinations, see S2 and S3, Electronic Supplementary Materials). Early Upper Paleolithic sites (**a**): *1* La Viña, *2* El Cierro, *3* Abrigo de la Güelga, *4* Conde (Forno), *5* Sopeña, *6* Esquilleu, *7* El Pendo, *8* El Ruso, *9* El Mazo de Camargo, *10* Cueva Morín, *11* El Rascaño, *12* Otero, *13* Cobrante, *14* El Polvorín, *15* Venta Laperra, *16* El Castillo, *17* Covalejos, *18* Santimamiñe, *19* Antoliñako Koba, *20* Labeko Koba, *21* Lezetxiki, *22* Coscobilo, *23* Cova Gran, *24* Reclau Viver, *25* L’Arbreda, *26* Mollet I, *27* Abric Romani, *28* Les Mallaetes, *29* Foradada, *30* Beneito, *31* Perneras, *32* Pirulejo, *33* Boquete de Zafarraya, *34* El Bajondillo, *35* Gorham’s Cave, *36* Pego do Diablo, *37* Gruta de Salemas. Middle Upper Paleolithic sites (**b**): *1* La Viña, *2* Sopeña, *3* Cueva de la Riera, *4* Cueto de la Mina, *5* Llonín, *6* Hornos de la Peña, *7* Altamira, *8* El Castillo, *9* El Pendo, *10* La Garma, *11* Cueva Morín, *12* El Rascaño, *13* El Mirón, *14* Abrigo del Cuco, *15* Bolinkoba, *16* Labeko Koba, *17* Lezetxiki, *18* Santimamiñe, *19* Antoliñako Koba, *20* Ermittia, *21* Aldatxarren, *22* Ekain, *23* Amalda, *24* Aitzbitarte III, *25* Torre, *26* Alkerdi, *27* Zatoya, *28* Roc de Melca, *29* Reclau Viver, *30* Davant Pau, *31* Mollet III, *32* L’Arbreda, *33* Balma de la Griera, *34* Beneito, *35* Parpallo, *36* Les Mallaetes, *37* Barranc Blanc, *38* Les Cendres, *39* Ratlla del Bubo, *40* Las Palomas, *41* Finca Doña Martina, *42* El Palomar, *43* Los Morceguillos, *44* Serrón-La Palica, *45* Nerja, *46* Boquette de Zafarraya, *47* El Bajondillo, *48* Gorham’s Cave, *49* Higueral de Motillas, *50* Higueral de Sierra Valleja, *51* Vale Boi, *52* Gruta de Salemas, *53* Lapa do Anecrial, *54* Casa da Moura, *55* Gruta do Caldeirão, *56* Lagar Velho, *57* Buraca Escura, *58* Buraca Grande, *59* Vale dos Covões (Abrigo 1), *60* Vale das Buracas

At the same time, zoogeographical reconstructions suggest that the Ebro Valley was in principle a permeable spatial feature connecting the faunal realms of northern Spain and southwestern France during MIS 3 (Stewart *et al.* 2003). Moreover, Banks *et al.* (2013) have recently argued that the general ecocultural fingerprint of Early Aurignacian and Protoaurignacian settlement does not exclude the colonization of the area south of the Ebro River, although site distributions reflect a clear rupture in site patterning there (Fig. 10a; compare S2, Electronic Supplementary Materials). These issues indicate that the pattern in question is difficult to explain by one-sided ecological approaches.

Yet—albeit not generally accepted—a range of Aurignacian sites seems to be present along the coast of southernmost Iberia (Schmidt *et al.* 2012; De la Peña and Vega Toscano 2013; Maíllo-Fernández 2013; Fig. 10a). Both chronometric evidence and technotypological information, however, place these assemblages into the later part of the regional Aurignacian sequence (Zilhão 2009; Maíllo-Fernández 2013); they therefore do not undermine the Ebro’s role as the mobility frontier in the pioneer phase of AMH dispersal in the Early Aurignacian. It thus appears that these coastal sites of an “evolved Aurignacian” signature (see Schmidt *et al.* 2012 for a recent summary) in southern Spain and eastern Portugal actually reflect a secondary dispersal event, several millennia after the initial establishment of AMH settlement on the peninsula. These sites are likely the result of mobility “windows” opened up by the eastern Mediterranean coastline that acts as a pulling factor and neutralizes the Ebro’s movement-limiting effect (see Fig. 10a). It is interesting to note that most Early Upper Paleolithic sites on the Iberian Peninsula are located within the wider Mediterranean drainage system (*cf*. Santisteban and Schulte 2007) which suggests an *intimate interweaving of fluvial and coastal regimes in this period*. From this perspective, it becomes clear that different spatial features often affect archaeological site organization quite differently and can both constrain or encourage each other. During the Early Upper Paleolithic, the Ebro Valley and the Mediterranean coast seem to be antagonists in this respect, the former largely confining mobility, the latter facilitating it.

The Ebro’s natural river profile is exceptional in many respects. Apart from having been a typical large-scale braided river system with extensive lateral channel movement and high water availability in the Late Pleistocene (*e.g.*, Luzón *et al.* 2008), the river has been characterized by an enormous lateral gravel bed and flood plain complex. Sedimentary and geomorphological evidence also points to higher sediment loads and flow velocities in the past (Wang *et al.* 2012). These biophysical characteristics might have effectively counterbalanced more favorable riparian landscape properties that are a feature of today’s Ebro catchment (*e.g.*, Magdaleno and Fernández-Yuste 2013). Based on these findings, it is interesting to note that during the Late Pleistocene, lateral doline formation created massive backswamp areas in ancient channel beds of the river system (Luzón *et al.* 2008). The combination of dynamic flow properties, wide floodplains, marshlands, and the mountain ranges of the *Sistema Ibérico* (Iberian Range) at the horizon has thus potentially constituted a very peculiar and almost “eerie” spatial setting that Early Upper Paleolithic groups might have encountered as “edge of the world.”

**Fig. 11:**
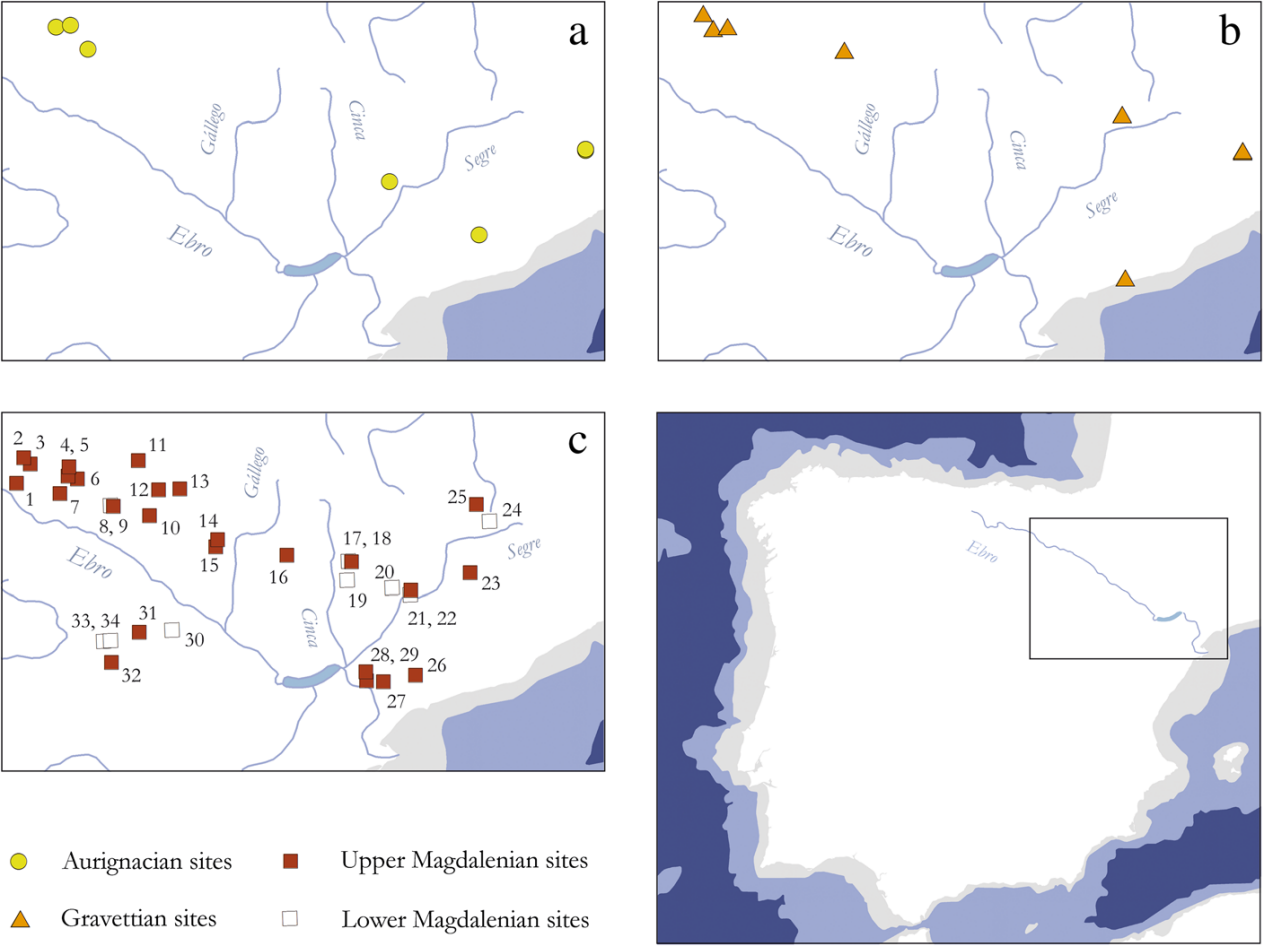
Site distribution from the Early Upper Paleolithic to the Magdalenian and Late Paleolithic in the Ebro catchment (spatial data from Schmidt *et al.* 2012 and Utrilla *et al.* 2012). The distribution pattern shows that a clear shift in settlement organization occurs during the Magdalenian period. Whereas in the Aurignacian (**a**) and Gravettian (**b**), sites are rarely located within the Ebro valley itself, the contrary can be observed in the Magdalenian and Later Paleolithic (**c**) where sites are even located on the western banks of the river system (for age determinations, see S4, Electronic Supplementary Materials). The shift in settlement preference indicates an important transformation of the Ebro’s role as a feature of spatial organization at the end of the Pleistocene: *1* Berniollo, *2* Urratxa, *3* Atrillon, *4* Anton Koba, *5* Kukuma, *6* Portugain, *7* Atxoste, *8* Legintxiki, *9* Leginpea, *10* Alaiz, *11* Abauntz, *12* Burutxukua, *13* Zatoya, *14* Peña 14, *15* Legunova, *16* Chaves, *17* Forcas I, *18* Forcas I, *19* Alonsé, *20* Cova Gran, *21* Parco, *22* Parco, *23* Guilanyá, *24* Montlleó, *25* Margineda, *26* Molí del Salt, *27* Boix, *28* Colls, *29* Hort de la Boquera, *30* Gato 2, *31* Bolichera, *32* Peña del Diablo 1/2, *33* Vergara, *34* Alejandre

The Gravettian occupation pattern in Iberia still signifies this frontier conceptualization and thus stands in Aurignacian “tradition.” Accordingly, the first phase of Gravettian presence in the region, like in Aurignacian times, is exclusively represented north of the Ebro basin, and the crossing of the river, again, occurred only in a later phase of the period (Schmidt *et al.* 2012; Fig. 11; compare S3, Electronic Supplementary Materials). In this regard, the coastal strip still acts as a conduit allowing for a swift dispersal of populations along the Mediterranean after ca. 32 ka cal. BP (Fig. 11). Taken together, the pattern of Gravettian site distribution through time exhibits strong similarities with the spatiotemporal structure of preceding Aurignacian sites in Iberia. It is possible that these similarities are a result of similar and partly shared pools of landscape knowledge and the relation of the latter to sociocultural understandings of particular landscape elements of the Ebro formation.

With the onset of the Magdalenian, however, the situation in the region changes dramatically and the Ebro Valley appears to be no major obstacle for human settlement anymore. Utrilla *et al.* (2012) have recently shown that the Ebro River system develops into a *crossroads* in this period, channeling both communication and mobility in northeastern Spain (see also Arrizabalaga *et al.* 2013): whereas during the Lower Magdalenian, most of the population was centered in the Jalón and Segre river basins, along communication axes toward the Meseta and southeastern France, respectively, the Middle Magdalenian was characterized by a “globalization” of interconnected communication vectors along the river system that can be documented on the basis of general affinities in material culture and portable art style in particular (Utrilla and Montes 2007; Montes and Utrilla 2008; Utrilla *et al.* 2012). The accentuation of the river as an important conduit for people and ideas is then paralleled by the general augmentation of spatial interconnectivity during the Magdalenian (see also Vanhaeren and d’Errico 2005).

In sum, archaeological site patterning on the Iberian Peninsula illustrates that the Ebro River system occupied different positions in different ecocultural systems. The role of the large biophysical formation in Upper Paleolithic spatial organization, therefore, not only reflects its natural characteristics but also the sociocultural context of human groups living in its vicinity. The Ebro is a perfect example to show that changing river understandings can be an important foundation of Upper Paleolithic spatiality. As a prominent spatial feature with “alienating” encounter properties, the river system effectively limited dispersal in the earlier part of the Upper Paleolithic while becoming an important catalyst for connecting sites and regions in its later part (Fig. 11; compare S4, Electronic Supplementary Materials). The shift in the river’s role from earlier to later Upper Paleolithic times can thus be interpreted as a *radical sociocultural reframing of the river*’*s persona* that is related to an intensification of social mobility and large-scale population dynamics.

### Late Upper Paleolithic Raw Material Movement Along the Rhine and the Danube

**Fig. 12:**
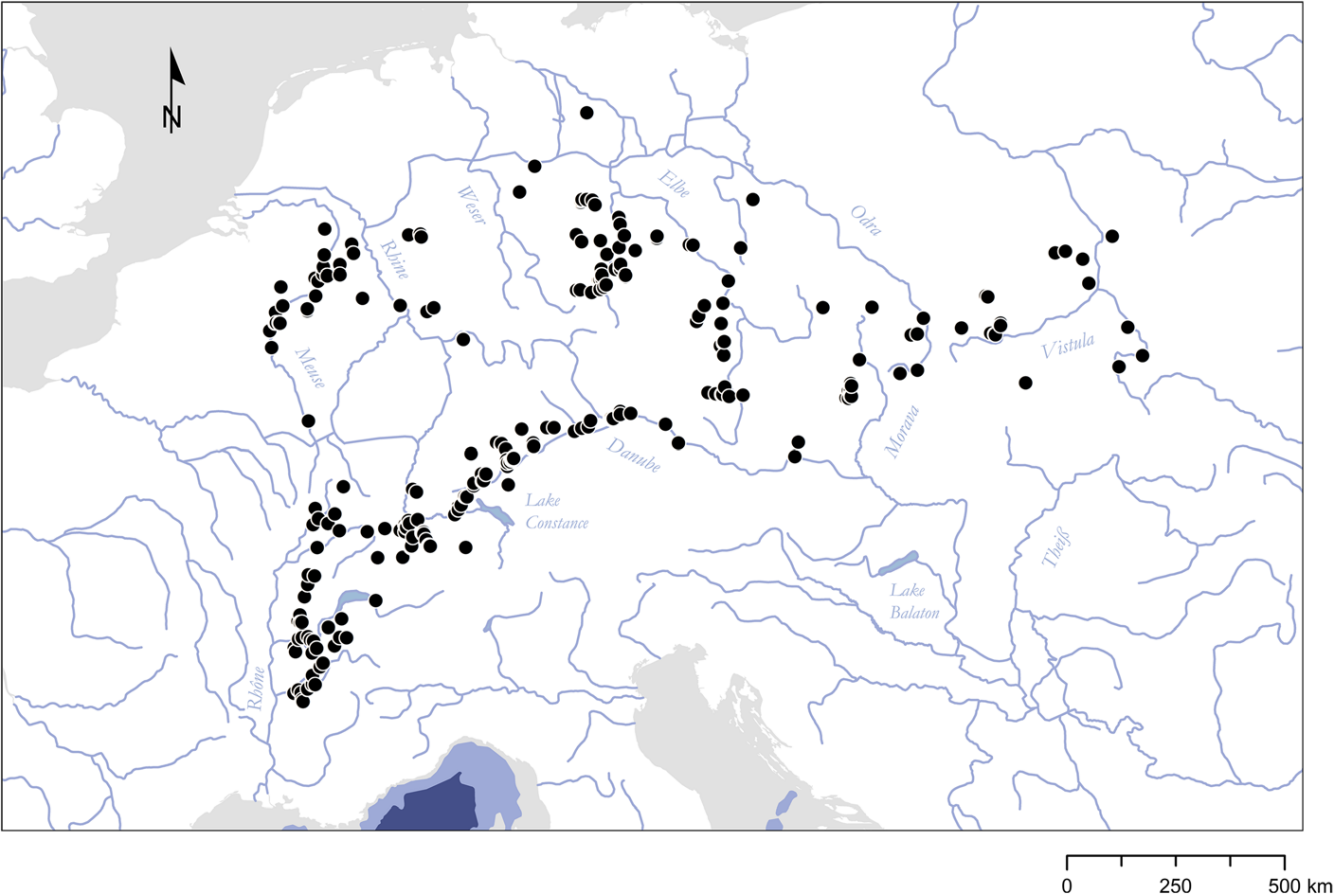
Site distribution of the Central European Magdalenian (spatial data with courtesy of Andreas Maier; see Maier 2012a for details). Sites are highly correlated with first-order rivers indicating an important role of fluvial systems in organizing Magdalenian sociocultural space (for site names and coordinates, see S5, Electronic Supplementary Materials)

**Fig. 13:**
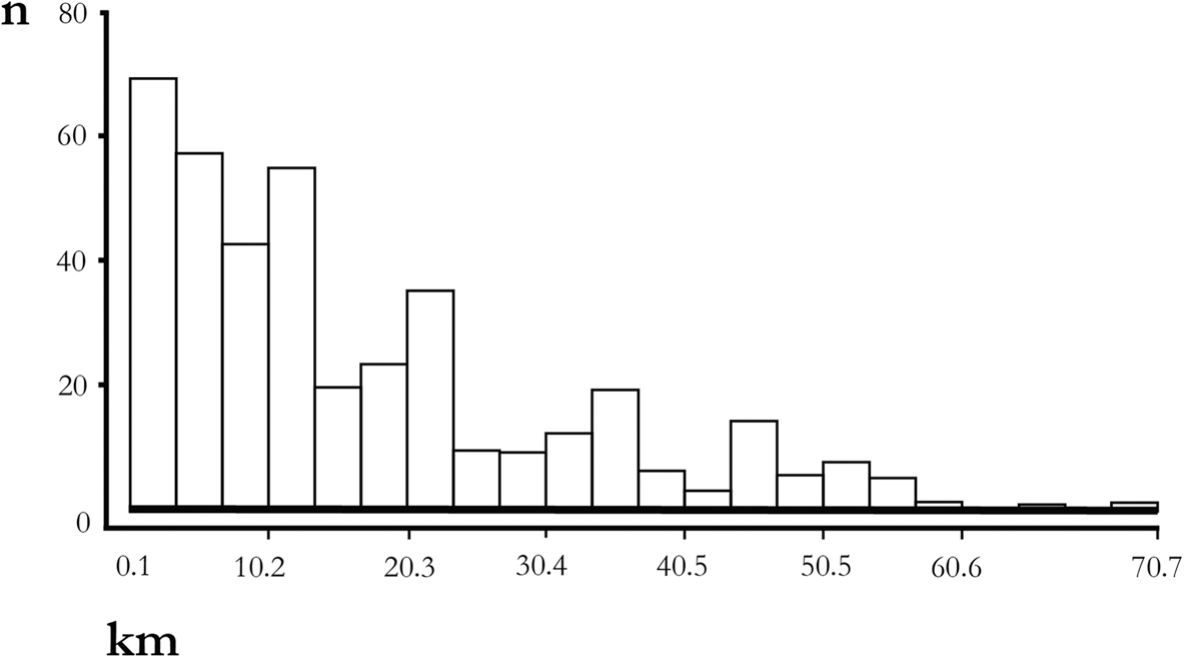
Frequency distribution illustrating the distance between Central European Magdalenian sites and first-order rivers (*x*-axis: distance to the next first-order river in kilometers, *y*-axis: number of sites per distance class). The data has been analyzed with the NEAR tool in ArcGIS 10.1 (spatial data with courtesy of Andreas Maier; see Maier 2012a for details)

The Central European Magdalenian offers a comprehensive case study for debating the role of large-scale river systems in the construction of sociocultural space during the Late Upper Paleolithic (*cf*. Hussain and Floss 2014). It is well known that the Magdalenian spatial performance portrays a strong affinity to river courses in general (*cf*. Floss 2003b; Bosinski 2008, p. 11; Maier 2012a). Even more radically, Maier (2012a, b) has recently shown that Magdalenian site distribution in Central Europe can be explained almost completely by river regime (see also Floss 2003b; Fig. 12). All but a few of the Magdalenian sites from this part of Europe are situated closer than 20 km to the next first-order river. This pattern is reflected in the reverse relationship between river distance and site frequency (Fig. 13), clearly indicating a crucial and up-to-now largely underestimated role of river systems in organizing the wider cultural geography of the Central European Magdalenian. It is interesting to note that in southwestern Germany, for example, there is even a positive relationship between larger sites documenting an intensified occupation, yielding both habitational structures *sensu lato* and portable art, and their proximity to large river valleys (Weniger 1987).

Yet, to date the most important archaeological archive for evidencing the interconnectivity of different sites that can be related to fluvial features in a second step is exotic raw materials (Floss 1994; Féblot-Augustins 1997; Eriksen 2002). The most relevant ones are nonlocal flint or chert, jet, and ornamental mollusks. Patterns in transportation vectors in the Magdalenian point to an important role of the north–south-directed Rhine-Saône-Rhône axis and the east–west-oriented Danube fluvial system in catalyzing the flow of these materials (Floss 1994, 2000a, b, 2002b, 2003b, 2009a; Féblot-Augustins 2009; Table 1).

**Table 1:** Selected sample of lithic/chert and mollusk raw material transport distances in the European Magdalenian that can be linked to the mediating role of the Rhône-Saône Formation, the Rhine River, and the Danube fluvial system

Site	Type	Variety	Source area	Distance of displacement (km)	Evidenced river axis	References
Gönnersdorf	Silex	Bohnerzjaspis	Upper Rhine region	300	Rhine	Floss 1994; Burkert and Floss 2005; Street *et al.* (2012)
Gönnersdorf	Silex	Chalzedony	Mainz Basin, western Germany	80–100	Rhine	Floss (1994), Street *et al.* (2012)
Gönnersdorf	Silex	Kieseloolith	Mainz Basin, western Germany	80–100	Rhine	Floss 1994; Street *et al.* (2012)
Gönnersdorf	Silex		Markgräflerland, southwestern Germany	290	Rhine-Saône	Maier (2012a, b)
Gönnersdorf	Mollusk	*Dentalium inaequicostatum*, *Dentalium*, *Vulgare*, *Homalopoma sanguineum*	Mediterranean, Rhône delta	800	Rhine-Saône-Rhône	Féblot-Augustins (1997, 2009), Álvarez-Fernandez (2009)
Andernach-Martinsberg	Mollusk	*Cyclope nerita*, *Homalopoma sanguineum*	Mediterranean, Rhône delta	800	Rhine-Saône-Rhône	Féblot-Augustins (1997, 2009), Álvarez-Fernandez (2009)
Dreiech-Götzenhain	Silex	Hornstein	Isteiner Klotz, southwestern Germany	230	Rhine	Terberger *et al.* (2013)
Probstfels	Silex	Chert	Kehlheim, Altmühl Valley, southern Germany	220	Danube	Maier (2012a, b)
Brillenhöhle	Silex	Chert	Kehlheim, Altmühl Valley, southern Germany	200	Danube	Maier (2012a, b)
Brillenhöhle	Silex	Tabular chert	Franconian Jura, southern Germany	160	Danube	Burkert and Floss (2005)
Brillenhöhle	Silex	Bohnerzjaspis	Upper Rhine region	180	Rhine	Burkert and Floss (2005)
Brillenhöhle	Silex	Kreidefeuerstein (Jurassic flint)	Franconian Jura?, southern Germany	190	Danube	Burkert and Floss (2005)
Hohle Fels	Silex	Plattenhornstein (tabular chert)	Franconian Jura (Abensberg), southern Germany	160	Danube	Floss (1994), Burkert and Floss (2005)
Hohle Fels	Silex	Tabular chert	Franconian Jura, southern Germany	160	Danube	Burkert and Floss (2005)
Hohle Fels	Silex	Bohnerzjaspis	Upper Rhine region	180	Rhine	Burkert and Floss (2005)
Hohle Fels	Silex	“Rocky crystal”	Upper Rhine region	180	Rhine	Burkert and Floss (2005)
Buttentalhöhle	Silex	Tabular chert	Franconian Jura, southern Germany	240	Danube	Burkert and Floss (2005)
Burkhartshöhle	Silex	Tabular chert	Franconian Jura, southern Germany	175	Danube	Burkert and Floss (2005)
Hohlenstein-Stadel	Silex	Tabular chert	Franconian Jura, southern Germany	40–120	Danube	Burkert and Floss (2005)
Bärenhöhle	Silex	Jurassic chert	Franconian Jura, southern Germany	40–120	Danube	Burkert and Floss (2005)
Bärenhöhle	Silex	Cretaceous quartzite	Fanconian Jura, southern Germany	80	Danube	Burkert and Floss (2005)
Bärenhöhle	Silex	Plattenhornstein (tabular chert)	Franconian Jura (Abensberg), southern Germany	120	Danube	Burkert and Floss )^2005^)
Bärenhöhle	Silex	Tabular chert	Franconian Alb, southern Germany	120	Danube	Burkert and Floss (2005)
Grappin-Arlay	Silex	“Silex café au lait”	Swiss Jura, Switzerland	70	Rhône-Danube	Cupillard and Welte (2006)
Grappin-Arlay	Silex		Etrelles, eastern France	70	Rhône-Rhine	Cupillard and Welte (2006)
Neuchâtel-Monruz	Silex		Mâconnais, eastern France	180	Rhône-Saône	Maier (2012a, b)
Neuchâtel-Monruz	Mollusk	*Brotis escheri*, *Viviparus suevicus*	Upper Danube Valley, southern Germany	270	Danube-Rhône	Maier (2012a, b)
Neuchâtel-Monruz	Silex		Singen, southwest Germany	170	Danube-Rhône	Maier (2012a, b)
Douattes	Silex		Olten-Wangen, southwest Germany	210	Danube-Rhône	Maier (2012a, b)
Douattes	Silex		Mâconnais?, eastern France	100	Rhône-Saône	Maier (2012a, b)
Veyrier	Silex		Olten-Wangen, southwest Germany	220	Danube-Rhône	Maier (2012a, b)
Veyrier	Silex		Mâconnais?, eastern France	110	Rhône-Saône	Maier (2012a, b)

In Central Europe, raw material movement along the Rhine is exemplified by the presence of “Bohnerzjaspis” (jasper) in the well-known Magdalenian site of Gönnersdorf, several hundred kilometers away from its source area in southwestern Germany (Floss 1991, 1994, 2009a). Although these pieces are likely to belong to the latest and more ephemeral Magdalenian phase, rather than to the main occupation of the site (Street *et al.* 2012), they point to the same spatial dislocation vector as informative material from the latter. The best example of such a link to the south from the main find concentration in Gönnersdorf is represented by a small group of chalcedony and “Kieseloolith” pieces that are likely to have been imported to the site from the Mainz basin, some 80 to 100 km away (Floss 1994; Street *et al.* 2012). Additional evidence for a north–south trajectory along the Rhine and its tributaries has recently been reported from the Magdalenian site of Dreiech-Götzenhain near Offenbach (Terberger *et al.* 2013). The assemblage has yielded a special “Hornstein” (chert) variety that can be traced back to the outcrops of Isteiner Klotz in southwestern Germany where the Upper Rhine meets the High Rhine.

Marine mollusks often indicate far more extended transfer vectors than exotic lithic or chert do. Andernach find concentration II, for example, produced subrecent ornamental mollusks of different species originating in the Mediterranean, at least 800 km to the south (Floss 1994, p. 218; Eriksen 2002). The provenance of marine mollusks from the Rhineland and the confluence area of the Upper and High Rhine in southwestern Germany in particular (Féblot-Augustins 1997, 2009; Floss 2002a; Álvarez-Fernandez 2009) is most likely the region around the Rhône delta. These materials, therefore, testify to the large-scale interconnectivity of the Rhône, the Saône, and the Rhine, that can also be detected, even though on smaller scales, in lithic raw material transfer signatures of several sites from Switzerland and eastern France (compare Table 1).

Evidence for the movement of lithic raw materials along the great Danube River during the Magdalenian is most notably constituted by “Plattenhornstein,” a special tabular chert variety that originates in the Franconian Jura (Floss 1994). Pieces of this particular raw material group appear in several Magdalenian sites of the Swabian Jura, bridging distances up to 160 km up-river and connecting both regions (Floss 1994, 2000a; Burkert and Floss 2005; Maier 2012a; compare Table 1). It is important to note that these raw materials are by no means the only physical indication for an east–west interconnectivity of the Central European Magdalenian parallel to the Danube. The Upper Danube area shares some striking sociocultural peculiarities, from which regularly red-dotted stones, often plaquettes, are particularly worth mentioning (Conard and Floss 1999; Conard and Uerpmann 2000; Floss and Conard 2001, 2009; Conard and Malina 2010). They have been found in Magdalenian layers of Hohle Fels and Kleine Scheuer in the Swabian Jura and, among others, in Obere Klause in the Altmühl Valley (Huber and Floss 2014; Conard *et al.* 2015, p. 109), thus clearly revealing an area of “common cultural heritage” and an enclosed communication space at the least (Floss 2014; Hussain and Floss 2014). These findings are consistent with Erikson’s (2002) suggestion that the emphasis on lithic raw material transfer (interpreted as expression of direct or embedded procurement) on this spatial axis would favor scenarios of actual group movement along the Danube. From this perspective, the material evidence clearly points to a mediating role of the Danube River system in the Magdalenian, channeling and regulating both the flow of people and materials.

**Fig. 14:**
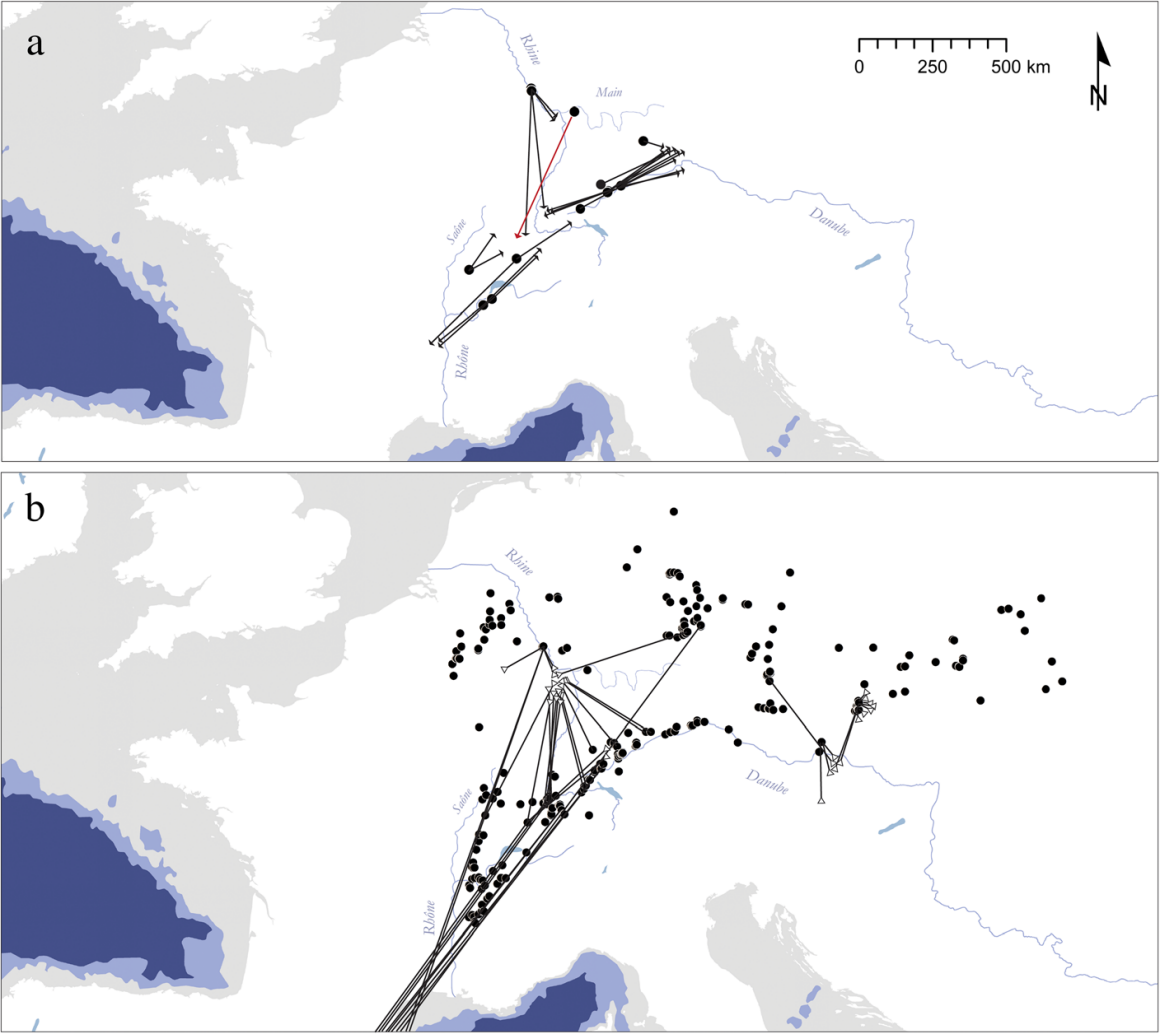
Relationship between the course of the Rhône-Saône formation, the Rhine River, and the Danube fluvial system and **a** lithic/chert (sample) and **b** mollusk/amber (total) displacement vectors in the Central European Magdalenian (*arrows* point/*triangles* mark the likely raw material source area). The connection between southwestern Germany and the Main area established by Dreiech-Götzenhain mentioned in the text is *highlighted* (modified and schematized after Floss 1994 and Maier 2012a; compare Table 1 for details)

It is thus plausible to understand lithic raw material movements as an expression of direct and embedded procurement within seasonal and/or annual cycles of social units (Floss 1994; Maier 2012a), while the transfer of ornamental mollusks is more likely to be rooted in intergroup communication systems, for example in interregional exchange networks (Eriksen 2002). If this interpretation is accepted, the current evidence seems to suggest that the connection of the north–south and east–west fluvial axes at the interface of eastern France and southwestern Germany can be characterized as a *spatial nexus* that controlled and mitigated the flow of people into both directions (Fig. 14). The spatial vector of intergroup communication, by contrast, is almost exclusively north–south oriented, which might indicate stronger social fragmentation along the latitudes. It certainly shows that large-scale river systems like the Rhine and the Danube had a huge impact on the spatial organization of the Central European Magdalenian in general and on patterns of mobility and contact in particular.

The idea that the main spatial vector for intergroup communication runs transversal to the main vector of residential intragroup mobility (*e.g.*, Eriksen 2002, p. 46) is supported by the recent discovery of a whale bone fragment in the Magdalenian faunal assemblage of Andernach-Martinsberg in the Neuwied Basin (Langley and Street 2013). The piece, although until now unparalleled, indicates contact zones between the Upper Rhine area and the Mediterranean coast of southwestern France where the only comparisons have been found so far. Drawing on the apparently strong “gravitational force” of great rivers in Magdalenian times, this finding seems to suggest that the most likely route behind this pattern is traveling along the Mediterranean coastline eastwards and then along the Rhine-Saône-Rhône axis northwards. The bridged distance, however, exceeds raw material displacement distances of lithics, chert, and mollusks by orders of magnitude and, thus, clearly favors a down-the-line transfer modality involving multiple groups. Additional evidence for a communication interface between the north–south-directed fluvial axis and the Mediterranean coast comes from seal engravings on slate plaques from both Gönnersdorf and Andernach (Bosinski and Bosinski 2009; Bosinski 2011, p. 45). Since not a single seal bone has been documented so far in the Central Rhineland, it seems very unlikely that seals were an endemic species or even migrated so far north during the Magdalenian (see Langley and Street 2013 for a detailed discussion). Hence, it must have been the idea rather than the animal that traveled considerable distance in space.

Essentially, the same argument can be made for the distribution of female silhouette depictions without head (*femmes sans tête*) in the later part of the Magdalenian. The main area of distribution for this “icône culturelle” (Bosinski 2011) is southwestern France, but the presence of the same motif has also been documented in a few sites along the great river axes of Central Europe (*e.g.*, Wagner 1984; Höck 1993; Bullinger 2006), for example in Magdalenian layers from Petersfels Cave near the crossing of Danube, Rhine, and Rhône-Saône formation where several related jet figures have been unearthed (Jelinek 1988). It is striking that various species of subrecent Mediterranean mollusks have been reported from the same layers (Rähle 1983; Maier 2012a, p. 116). Additional evidence comes from the Magdalenian site of Gönnersdorf in the Neuwied Basin further up the Rhine where similar representations of female silhouettes appear as engravings on stone plaquettes (Bosinski 2011, 69ff.).

The pattern of sociocultural interconnectivity along the Rhine and the Danube axes is partly related to climatic and environmental parameters of the time that define the place of both rivers in the wider land- and taskscape. In Central Europe, the Magdalenian marks the onset of a recolonization phase after severe glacial conditions during the Last Glacial Maximum (LGM) where most of the area has been uninhabitable. Rivers were therefore part of flattened steppe-tundra landscapes, where LGM conditions have filled up the river valleys with sediment loads. Reduced discharge values in tandem with starting alpine glacier melting resulted in wide, braided river characteristics (*cf*. Ward *et al.* 2002; Lóczy 2007; Miklós and Neppel 2010; Wang *et al.* 2012) that grant fluvial systems a high degree of accessibility and visibility and, thus, a significant degree of spatial prominence. These landscapes are largely river-dominated—“riverscapes” so to speak—while vegetation is only slowly returning to Central Europe. Greater numbers of boreal trees along the Danube, for example, are only documented from about 16 ka cal. BP onward in the paleoenvironmental record of the region (*e.g.*, Samartin *et al.* 2012; Heiri *et al.* 2014). The important role of the Danube and the Rhine river systems in organizing the sociocultural space of the Central European Magdalenian can therefore be rooted in the peculiarities of experiencing this particular spatial setting and encountering the affordance structure of large rivers within it.

It is maybe crucial to acknowledge that the specific articulation of ecoclimatic conditions and a sociocultural colonization background is reminiscent of the proposed scenario for the Early Upper Paleolithic and its river management. Analogously, it can be argued that much of the in-depth landscape knowledge about Central Europe has likely been lost over the course of the LGM and the associated population retreat. The reintroduction of human settlement into the region can thus be paralleled again with an exploratory stage of landscape learning (*sensu* Gärling *et al.* 1984). From this perspective, it is easier to understand why large river systems seem to make their reappearance as powerful catalysts for both communication and mobility in and after the recolonization of Central Europe by Magdalenian groups.

Although it has been argued so far that during the Central European Magdalenian the notion of river conduits clearly prevails, frontier situations constituted by fluvial features can nevertheless be identified on finer grained scales of analysis. The Magdalenian occupation pattern in the area where the Limmat, Reuss, and Aar rivers flow together and join the High Rhine provides a good example here. Despite the close spatial proximity of Magdalenian sites between the border area of the Swiss and the Swabian Jura, their respective site clusters appear to have exploited different and almost exclusive raw material sources. Moreover, although site density is generally high on both sides (east and west) of the confluence, not a single Magdalenian site has been documented so far in the area itself (Maier 2012a: Fig. 30). Consequently, Maier (Maier 2012a, b, p. 98) has recently interpreted this finding as the expression of an internal Magdalenian border, separating two regional groups with virtually no mutual interaction. The advent of Magdalenian settlement in both regions is only marginally offset, starting around 17 ka cal. BP in the Swiss Jura and around 16 to 16.5 ka cal. BP in the Swabian Jura, while both remain continuously occupied until ca. 14 ka cal. BP (Housley *et al.* 1997). Hence, combined chronospatial evidence clearly points to the contemporaneity of both groups and thus, by implication, to the social nature of the isolation pattern.

## Discussion

Approaching human-environment interactions within the framework of ecocultural system theory allows for the meaningful integration of ecological, geomorphological, and archaeological data of the distant past. Each ecocultural system is unique and defined by the precise structure of relationships between climatic, environmental, and physiographic variables on the one hand, and the modalities of sociocultural organization on the other. Although both dimensions influence each other mutually, the relationship is not necessarily symmetrical. Hierarchical regulation is a crucial property of such dynamic hybrid systems. Various variables can effectively *constrain* each other on different organizational levels and in very different ways (Farina 2010, 127ff.). One way to capture the organizational structure of ecocultural systems is to explore their underlying logic of selection and significance in conjunction with the heterogeneous nature of their constitutive parts (*cf*. Kockelman 2011). Within this framework, focality, heuristics, affordance structures, and entangled relationships become central concepts to address the issue and to determine the specific place of discrete spatial features in larger ecocultural systems.

Debating the role of large Pleistocene river systems in the spatiocultural organization of Upper Paleolithic units, therefore, warrants the shift in focus from the natural configuration of fluvial features and the cultural configuration of archaeological entities to the *structural relationship* between the two. Structural similarity and structural difference are critical concepts for understanding the place of both people and rivers within the wider ecocultural system of their time. From this point of view, it is interesting to note that the structural relationship between large rivers, their wider ecoclimatic context, the spatial patterning of sites, and larger sociohistorical constellations appear to be rather similar in the European Early Upper Paleolithic and Late Upper Paleolithic, whereas the Middle Upper Paleolithic seems to deviate from this pattern.

In the first case, accessible, wide, and multichannel river systems in relatively cold climates and open environments co-occur with a pronounced interconnectedness of sites along major drainage axes that are related to colonization events *sensu lato*. Landscape knowledge is poor and large river systems are highly focal features in the landscape, thus inviting people to exploit rivers heuristically, resulting in fluvial conduits. At the same time, it is clear that the precise sociocultural expressions underlying this structural relationship are far from being identical for both time slices, indicating some substantial differences in the exploitation of comparable focality and affordance structures over time. Evidence for the Early Aurignacian, for instance, points to small-scale site interconnectivity, which is reflected in restricted regions of “common cultural heritage” and embedded raw material procurement along the fluvial conduits (Féblot-Augustins 1997; Djindjian *et al.* 1999). In the Central European Magdalenian, on the other hand, site interconnectivity is often large scale (*e.g.*, Floss 1994, p. 326; Brantingham 2006) and has been described as a “small-world network” (Maier 2012b). Rivers in such a context not only catalyze movement but also facilitate the distribution of materials and ideas across multiple groups (Féblot-Augustins 1997, 2009; Conard and Floss 1999; Floss 2000a, b, 2002b; Langley and Street 2013). It is hence very tempting to argue that the intimate relationship between Magdalenian sites and large river systems has initially been shaped tacitly during the recolonization process of Central Europe after the LGM and was later exploited to organize and structure the social realm. Simultaneously, Magdalenian spatial patterning from the confluence area of the Limmat, Reuss, and Aar exemplifies that structuring social groups can easily turn into separating them. Notwithstanding, on a pan-European scale, the dominant role of rivers in the Magdalenian is clearly one of a *spatial nexus*. This pattern seems to be robust and is, for example, also well reflected in the chronospatial record of the Ebro Valley in Western Europe, where the river develops into crossroads during the Magdalenian (Utrilla *et al.* 2012; Arrizabalaga *et al.* 2013), holding comparable properties as the fluvial axes of the Rhône, Saône, Rhine, and Danube in Central Europe (Hussain and Floss 2014). The presence of small-scale river boundaries in this setting, as indicated by the Limmat-Reuss-Aar formation for example, might simply reflect the need for buffering against conflict potential during and/or shortly after dispersal processes (see Gutiérrez Nájera 2009 for an anthropological example) and might be a promising avenue for future research. During the initial colonization of Europe in the Early Upper Paleolithic by AMH groups, this aspect might indeed be a critical factor because conflict potential is not only a matter of internal “negotiation,” since—although debatable—AMHs and endemic Neanderthals might have actually encountered each other as the former set foot on the continent for the first time (d’Errico *et al.* 1998; Mellars 2006a; Weninger and Jöris 2008; Higham *et al.* 2014).

To sum up, both in the European Early Upper Paleolithic and in the Late Upper Paleolithic, large river systems support the internal integrity of their sociocultural realms, but at the same time, significant differences in how the offered opportunities are integrated in the wider ecocultural system can be observed. It is interesting to note here that this finding enigmatically reiterates Jared Diamond’s (2009) famous argument on the unique geographic structure of Central and Western Europe with its pronounced natural axes and a highly interconnected hydrological system that should have affected the continent’s development ever since.

The Middle Upper Paleolithic reveals a different picture. There, highly variable river characteristics that stretch a continuum from braided to meandering with an often abrupt transition between the two co-occur with the fragmentation of the social landscape during the process of settlement consolidation after the successful dispersal of AMHs in earlier periods (*cf*. Noiret 2013). In this setting, the formation of regional identities and a much more consistent and larger scale emergence of “areas of common cultural heritage” than before (see Roebroeks *et al.* 2000; Otte 2013 and references therein) signal an intensified relationship between the landscape and its inhabitants. Landscape knowledge has thus expected to be rather elaborated and highly entangled with sociocultural beliefs and values. Accordingly, the conceptualization of rivers and their integration to the ecocultural system is generally variable, following the logic of the adaptive and abstract stages of “environmental legibility” of Gärling *et al.* (1984). The case of the Garonne, for example, nicely exemplifies that the internal diversification of the Gravettian world is fostered by the river, anchoring a social threshold in space and thereby separating different spheres of interaction (Klaric *et al.* 2009; Simonet 2012). It is, of course, an open question whether this finding represents a large-scale trend in the European Middle Upper Paleolithic, but at least it seems to be clear that the emphasis on spatial segregation is a new dimension of river significance that fits perfectly into the wider context of the period.

Evidently, many factors play a decisive role in the formation of an ecocultural system. Differently organized ecocultural systems can also accommodate for different behavioral performances and their archaeological correlates. A river’s role can thus change through time and the entire web of structural relationships that characterizes its systemic context changes with it. It is in this sense that river courses and their shifting role in organizing sociocultural space can serve as heuristic devices to better understand the deeply embedded and entangled nature of Upper Paleolithic lifeways. The human-river relationship, therefore, virtually helps us to get a better grasp on what is happening at the systemic level.

The recognition that the architecture of ecocultural systems actually matters also implies that—although some spatial features are often more important than others—concurring landscape constituents such as mountains, lowlands, and plains play their *own* part in shaping the spatiality of Upper Paleolithic entities. The issue is, of course, complicated by the fact that many of these features are practically difficult to separate from each other. Hence, studying the impact of river courses on past modalities of spatial organization can only be the first step on the way to better understand the spatial dimension of the archaeological record.

## Conclusion

Pleistocene river systems are part of ecocultural systems. As such, their role in and impact on Upper Paleolithic spatial organization is largely dependent on their position within the structure of relationships that make up the system. Because these relationships are rarely congeneric and thus in most cases both multiscale as well as hierarchically organized, it is crucial to theorize the relative importance of different system components for the actual performance of the system. This is particularly necessary because ecocultural systems are hybrid systems that include human sociocultural units and ecological as well as biophysiographic “agents.” Several factors ensure that rivers frequently occupy an important position in these systems: large river systems anchor behavior of mobile agents spatially because of their visual and ecological focality, they present powerful affordance and heuristic structures that invite agents to organize their spatial behavior in certain nonarbitrary ways, and they bring natural and cultural landscape dimensions closely together by being prominent carriers of meaning and significance. These factors are of course interrelated, but they clearly demonstrate that the story of Upper Paleolithic river systems is also the story of Upper Paleolithic societies—and the other way around. There is at least an intimate structural relationship between the way such fluvial features present themselves, how they are encountered and experienced, and how they are conceptualized and finally integrated into the architecture of the wider ecocultural system.

Tracing these relationships throughout the Central and Western European Upper Paleolithic reveals some interesting overarching patterns and shows that the categorization of large river systems on a continuum between *frontiers* and *trajectories* is a fruitful approach. It also demonstrates that these notions are differently negotiated in relation to different sociocultural domains, for example in relation to group mobility, object mobility, and communication. These differences once again emphasize the organizational exceptionality of various historically situated ecocultural systems and the importance to discuss these peculiarities in relation to spatiality.

In particular, two distinct patterns of systemic river integration can be identified in the Central and Western European Upper Paleolithic on a continental scale: the first is characterized by the entanglement of cold climate regimes, accessible river systems in open environments, colonization scenarios, and poor landscape knowledge and signifies the Early Upper Paleolithic and the Late Upper Paleolithic after the LGM. The second is marked by the entanglement of variable climatic conditions, dynamic river systems in changing environments, consolidation of the European settlement after successful dispersal, as well as rich landscape knowledge and can broadly be placed in the Middle Upper Paleolithic. The former is an articulation of explorative stages in human-environment relationships supporting a conduit notion of river courses, whereas the latter reflects an adaptive and more cemented stage and denotes the increasing weight of fluvial features that express and reproduce sociocultural boundaries.

These findings once again show that moving beyond the dichotomy of nature and culture is a guiding imperative for upcoming research in the field (*cf*. Wilcock *et al.* 2013). They also stress the importance of better theorizing the structural relationships between different empirical archives in order to integrate them in a meaningful way. Debating the totality of past human-environment relationships within the framework of ecocultural systems might contribute to this endeavor.

## Electronic Supplementary Materials

Below is the link to the electronic supplementary material.

ESM 1 (DOCX 102 kb)
