# Supplementary material for: Streams as Entanglement of Nature and Culture: European Upper Paleolithic River Systems and Their Role as Features of Spatial Organization
Source: J Archaeol Method Theory. 2015 Oct 7;23(4):1162–218. doi: 10.1007/s10816-015-9263-x (PMC5750683; doi:10.1007/s10816-015-9263-x)
Supplement: Supplementary file 1 — (DOCX 102 kb) [file 10816_2015_9263_MOESM1_ESM.docx]

Supplementary materials

**S1** Published ^14^C-dates for Early Upper Paleolithic sites mapped in **Fig. 5**. Dates have been calibrated with CalPal using the Intcal09 calibration curve (site-order according to an east-west transect).

| **Site** | **Lab code** | **14C age** | **14C std** | **calBP** | **calBP std** | **Material** | **Layer** | **Method** | **References** |
| --- | --- | --- | --- | --- | --- | --- | --- | --- | --- |
| Kostenki 14 | OxA-9568 | 32,600 | 280 | 37,370 | 580 | Charcoal | IVb | AMS | Sinitsyn 2003; Jöris et al. 2011 |
| Kostenki 14 | GrA-13302 | 34,940 | 630 | 40,000 | 950 | Charcoal | IVb | AMS | Sinitsyn 2003; Jöris et al. 2011 |
| Kostenki 14 | OxA-9569 | 35,280 | 330 | 40,290 | 650 | Charcoal | IVb | AMS | Sinitsyn 2003; Jöris et al. 2011 |
| Kostenki 14 | GrA-15957 | 36,040 | 250 | 41,190 | 360 | Charcoal | IVb | AMS | Sinitsyn 2003; Jöris et al. 2011 |
| Kostenki 14 | GrA-15961 | 36,540 | 270 | 41,550 | 320 | Charcoal | IVb | AMS | Sinitsyn 2003; Jöris et al. 2011 |
| Kostenki 14 | GrA-15958 | 35,330 | 240 | 40,390 | 540 | Charcoal | Hearth | AMS | Sinitsyn 2003; Jöris et al. 2011 |
| Kostenki 14 | GrA-15962 | 35,870 | 250 | 41,040 | 380 | Charcoal | Hearth | AMS | Sinitsyn 2003; Jöris et al. 2011 |
| Kostenki 14 | GrA-15965 | 36,010 | 250 | 41,160 | 360 | Charcoal | Hearth | AMS | Sinitsyn 2003; Jöris et al. 2011 |
| Kostenki 14 | GrA-15956 | 36,320 | 270 | 41,420 | 330 | Charcoal | Hearth | AMS | Sinitsyn 2003; Jöris et al. 2011 |
| Kostenki 14 | GrA-10948 | 37,240 | 430 | 42,030 | 440 | Charcoal | Hearth | AMS | Sinitsyn 2003; Jöris et al. 2011 |
| Bacho Kiro | OxA-3183 | 37,650 | 1450 | 42,040 | 1630 | Charcoal | 11 | AMS | Database Stage 3 Project |
| Bacho Kiro | OxA-3212 | 34,800 | 1150 | 39,380 | 1760 | Tooth | 11 | AMS | Database Stage 3 Project |
| Bacho Kiro | OxA-3213 | 38,500 | 1700 | 42,820 | 1700 | Bone | 11 | AMS | Database Stage 3 Project |
| Bacho Kiro | GrN-7569 | 32,700 | 300 | 37,520 | 660 | Charcoal | 6b | conventional | Database Stage 3 Project |
| Bacho Kiro | OxA-3182 | 33,300 | 820 | 38,300 | 1310 | Charcoal | 6b | AMS | Database Stage 3 Project |
| Bacho Kiro | OxA-3181 | 32,200 | 780 | 36,870 | 1260 | Charcoal | 7 | AMS | Database Stage 3 Project |
| Kozarnika | GifA-99706 | 36,200 | 540 | 41,190 | 610 |  | VII |  | Tsanova et al. 2012 |
| Kozarnika | GifA-101050 | 37,170 | 700 | 41,970 | 660 |  | VII |  | Tsanova et al. 2012 |
| Kozarnika | GifA-10994 | 38,700 | 140 | 42,990 | 260 |  | VII |  | Tsanova et al. 2012 |
| Kozarnika | GifA-99662 | 39,310 | 100 | 43,500 | 340 |  | VII |  | Tsanova et al. 2012 |
| Franchthi | OxA-21069 | 23,510 | 90 | 28,270 | 190 | Shell | R | AMS | Douka et al. 2011 |
| Franchthi | OxA-20615 | 32,110 | 200 | 36,650 | 200 | Shell | R | AMS | Douka et al. 2011 |
| Franchthi | OxA-20616 | 35,600 | 255 | 40,780 | 420 | Shell | P | AMS | Douka et al. 2011 |
| Franchthi | OxA-21070 | 41,080 | 390 | 44,780 | 420 | Shell | R | AMS | Douka et al. 2011 |
| Franchthi | OxA-22270 | 29,780 | 160 | 34,470 | 230 | Shell | Q | AMS | Douka et al. 2011 |
| Franchthi | dupl. | 30,580 | 160 | 35,000 | 180 | Shell | Q | AMS | Douka et al. 2011 |
| Franchthi | OxA-20253 | 34,980 | 220 | 40,030 | 610 | Shell | Q | AMS | Douka et al. 2011 |
| Franchthi | OxA-21351 | 26,910 | 120 | 31,270 | 110 | Shell | P | AMS | Douka et al. 2011 |
| Franchthi | OxA-21115 | 30,410 | 160 | 34,900 | 160 | Shell | P | AMS | Douka et al. 2011 |
| Franchthi | GifA-80104/SacA-11206 | 32,110 | 330 | 36,460 | 640 | Charcoal | R | AMS | Douka et al. 2011 |
| Franchthi | GifA-09381/SacA-15334 | 33,250 | 420 | 37,830 | 760 | Calcite? | Q | AMS | Douka et al. 2011 |
| Grotta del Cavallo | OxA-21072 | 19,685 | 75 | 23,580 | 200 | Shell | D1 | AMS | Benazzi et al. 2011 |
| Grotta del Cavallo | dupl. | 19,235 | 75 | 22,930 | 320 | Shell | D1 | AMS | Benazzi et al. 2011 |
| Grotta del Cavallo | OxA-19254 | 35,080 | 230 | 40,140 | 580 | Shell | D1=DIb | AMS | Benazzi et al. 2011 |
| Grotta del Cavallo | OxA-19255 | 36,260 | 250 | 41,390 | 310 | Shell | D2=DIb | AMS | Benazzi et al. 2011 |
| Grotta del Cavallo | OxA-20631 | 36,780 | 310 | 41,700 | 340 | Shell | DII | AMS | Benazzi et al. 2011 |
| Grotta del Cavallo | OxA-19257 | 42,360 | 400 | 45,580 | 440 | Shell | D3=DII | AMS | Benazzi et al. 2011 |
| Grotta del Cavallo | OxA-19258 | 36,000 | 400 | 41,100 | 510 | Shell | D8=DII? | AMS | Benazzi et al. 2011 |
| Grotta del Cavallo | OxA-19256 | 39,060 | 310 | 43,310 | 440 | Shell | E1=E-D | AMS | Benazzi et al. 2011 |
| Grotta del Cavallo | OxA-X-2280-16 | 38,300 | 400 | 42,710 | 430 | Shell | E1=E-D | AMS | Benazzi et al. 2011 |
| Grotta del Cavallo | OxA-19242 | 39,990 | 340 | 43,930 | 470 | Shell | E4=EII-I | AMS | Benazzi et al. 2011 |
| Senftenberg | GrN-16887 | 36,350 | 600 | 41,300 | 650 | Charcoal |  | AMS | Nigst and Haesaerts 2012 |
| Willendorf II | GrN-17805 | 38,880 | 1530 | 43,200 | 1460 | Charcoal | 3 | AMS | Nigst and Haesaerts 2012 |
| Willendorf II | GrN-11192 | 34,100 | 1200 | 39,000 | 1760 | Charcoal | 3 | AMS | Nigst and Haesaerts 2012 |
| Willendorf II | GrA-896 | 37,930 | 750 | 42,520 | 720 | Charcoal | 3 | AMS | Nigst and Haesaerts 2012 |
| Willendorf II | GrN-1273 | 32,060 | 250 | 36,630 | 230 | Charcoal | 4 | AMS | Nigst and Haesaerts 2012 |
| Willendorf II | GrA-501 | 31,210 | 260 | 35,750 | 500 | Charcoal | 4 | AMS | Nigst and Haesaerts 2012 |
| Willendorf II | H-249/1276 | 31,700 | 1800 | 36,390 | 2860 | Charcoal | 4 | AMS | Nigst and Haesaerts 2012 |
| Keilberg | KN-4690 | 37,500 | 1450 | 41,890 | 1670 | Charcoal | 2a | conventional | Uthmeier 2004 |
| Keilberg | KN-4691 | 37,500 | 1205 | 42,180 | 1280 | Charcoal | 2a | conventional | Uthmeier 2004 |
| Keilberg | KN-4692 | 38,600 | 1200 | 43,020 | 1170 | Charcoal | 2a | conventional | Uthmeier 2004 |
| Grotta di Fumane | OxA-17981 | 33,890 | 220 | 38,880 | 290 | Charcoal | D3b | AMS-ABOx | Higham et al. 2009 |
| Grotta di Fumane | OxA-17569 | 35,640 | 220 | 40,830 | 380 | Charcoal | A2 | AMS-ABOx | Higham et al. 2009 |
| Grotta di Fumane | OxA-19584 | 35,850 | 310 | 41,000 | 440 | Charcoal | A2 | AMS-ABOx | Higham et al. 2009 |
| Grotta di Fumane | OxA-19414 | 34,180 | 270 | 39,310 | 510 | Charcoal | A2 | AMS-ABOx | Higham et al. 2009 |
| Grotta di Fumane | OxA-19412 | 34,940 | 280 | 39,970 | 670 | Charcoal | A2 | AMS-ABOx | Higham et al. 2009 |
| Grotta di Fumane | OxA-17570 | 35,180 | 220 | 40,250 | 550 | Charcoal | A2 | AMS-ABOx | Higham et al. 2009 |
| Geißenklösterle | OxA-21725 | 37,400 | 800 | 42,110 | 750 | Bone | GK 86 III 294 | AMS ultrafiltration | Higham et al. 2012 |
| Geißenklösterle | OxA-21726 | 34,200 | 550 | 39,110 | 1060 | Bone | GK 55 IId 319 | AMS ultrafiltration | Higham et al. 2012 |
| Geißenklösterle | OxA-21659 | 35,050 | 600 | 40,080 | 930 | Bone | GK 77 III 627 | AMS ultrafiltration | Higham et al. 2012 |
| Geißenklösterle | OxA-21744 | 36,850 | 750 | 41,680 | 760 | Bone | GK 77 III 641 | AMS ultrafiltration | Higham et al. 2012 |
| Geißenklösterle | OxA-21745 | 36,650 | 750 | 41,510 | 790 | Bone | GK 66 IIIa 1073 | AMS ultrafiltration | Higham et al. 2012 |
| Geißenklösterle | OxA-21746 | 36,850 | 800 | 41,660 | 820 | Bone | GK 67 IIIa 1453 | AMS ultrafiltration | Higham et al. 2012 |
| Geißenklösterle | OxA-21722 | 38,900 | 1000 | 43,230 | 980 | Bone | GK 66 III 1144 | AMS ultrafiltration | Higham et al. 2012 |
| Geißenklösterle | OxA-21743 | 36,100 | 700 | 40,870 | 920 | Bone | GK 67 IIIb 1655 | AMS ultrafiltration | Higham et al. 2012 |
| Geißenklösterle | OxA-21723 | 37,800 | 900 | 42,470 | 900 | Bone | GK 69 IIIb 958 | AMS ultrafiltration | Higham et al. 2012 |
| Geißenklösterle | OxA-21721 | 37,300 | 800 | 42,050 | 740 | Bone | GK 57 IIIb 1238 | AMS ultrafiltration | Higham et al. 2012 |
| Hohle Fels | KIA 32056 | 29,710 | 210 | 34,230 | 380 | Bone | IIIa Feature 1 | AMS | Conard and Bolus 2008; Conard 2009 |
| Hohle Fels | KIA 32055 | 30,340 | 290 | 34,890 | 230 | Bone | IIIa Feature 1 | AMS | Conard and Bolus 2008; Conard 2009 |
| Hohle Fels | KIA 16038 | 29,840 | 210 | 34,490 | 270 | Bone | IIIa | AMS | Conard and Bolus 2008; Conard 2009 |
| Hohle Fels | KIA 18877 | 30,170 | 250 | 34,800 | 190 | Charcoal | IIIa | AMS | Conard and Bolus 2008; Conard 2009 |
| Hohle Fels | OxA-4601 | 30,550 | 550 | 35,450 | 660 | Bone | IIIa | AMS | Conard and Bolus 2008; Conard 2009 |
| Hohle Fels | KIA 18876 | 31,010 | 600 | 35,640 | 740 | Charcoal | IIIa | AMS | Conard and Bolus 2008; Conard 2009 |
| Hohle Fels | KIA 16039 | 31,140 | 250 | 35,710 | 500 | Bone | IIIa | AMS | Conard and Bolus 2008; Conard 2009 |
| Hohle Fels | KIA 18878 | 29,780 | 330 | 34,230 | 500 | Charcoal | IIIb | AMS | Conard and Bolus 2008; Conard 2009 |
| Hohle Fels | KIA 3505 | 29,990 | 340 | 34,530 | 370 | Bone | IIIb | AMS | Conard and Bolus 2008; Conard 2009 |
| Hohle Fels | KIA 32060 | 30,110 | 220 | 34,780 | 170 | Bone | IV Feature 6 | AMS | Conard and Bolus 2008; Conard 2009 |
| Hohle Fels | KIA 32058 | 30,420 | 220 | 34,910 | 200 | Bone | IV Feature 6 | AMS | Conard and Bolus 2008; Conard 2009 |
| Hohle Fels | KIA 32059 | 30,460 | 250 | 34,950 | 220 | Bone | IV Feature 6 | AMS | Conard and Bolus 2008; Conard 2009 |
| Hohle Fels | OxA-4980 | 28,750 | 750 | 33,110 | 1160 | Bone | IV | AMS | Conard and Bolus 2008; Conard 2009 |
| Hohle Fels | KIA 32057 | 30,040 | 210 | 34,750 | 160 | Bone | IV | AMS | Conard and Bolus 2008; Conard 2009 |
| Hohle Fels | OxA-4600 | 31,100 | 600 | 35,670 | 720 | Bone | IV | AMS | Conard and Bolus 2008; Conard 2009 |
| Hohle Fels | KIA 18879 | 31,160 | 1530 | 35,580 | 2310 | Charcoal | IV | AMS | Conard and Bolus 2008; Conard 2009 |
| Hohle Fels | KIA 16037 | 32,470 | 290 | 37,110 | 440 | Bone | IV | AMS | Conard and Bolus 2008; Conard 2009 |
| Hohle Fels | KIA 16036 | 33,090 | 260 | 37,740 | 640 | Bone | IV | AMS | Conard and Bolus 2008; Conard 2009 |
| Hohle Fels | KIA 35464 | 31,750 | 260 | 36,060 | 480 | Bone | Va | AMS | Conard and Bolus 2008; Conard 2009 |
| Hohle Fels | KIA 35463 | 32,030 | 280 | 36,310 | 530 | Bone | Va | AMS | Conard and Bolus 2008; Conard 2009 |
| Hohle Fels | KIA 35462 | 32,090 | 350 | 36,450 | 650 | Bone | Va | AMS | Conard and Bolus 2008; Conard 2009 |
| Hohle Fels | KIA 35460 | 32,370 | 280 | 36,990 | 380 | Bone | Va | AMS | Conard and Bolus 2008; Conard 2009 |
| Hohle Fels | KIA 35459 | 32,550 | 300 | 37,260 | 520 | Bone | Va | AMS | Conard and Bolus 2008; Conard 2009 |
| Hohle Fels | OxA-19783 | 31,760 | 200 | 36,100 | 440 | Bone | Va 10 | AMS | Conard 2009 |
| Hohle Fels | OxA-19859 | 34,570 | 260 | 39,650 | 590 | Bone | Va 10 | AMS | Conard 2009 |
| Hohle Fels | KIA 16035 | 33,290 | 270 | 37,880 | 630 | Bone | Vb | AMS | Conard and Bolus 2008; Conard 2009 |
| Hohle Fels | KIA 18880 | 34,190 | 340 | 39,360 | 580 | Charcoal | Vb | AMS | Conard and Bolus 2008; Conard 2009 |
| Hohle Fels | KIA 16034 | 35,710 | 360 | 40,840 | 520 | Bone | Vb | AMS | Conard and Bolus 2008; Conard 2009 |
| Hohle Fels | OxA-19860 | 31,290 | 180 | 35,780 | 460 | Charcoal | Vb | AMS | Conard 2009 |
| Hohle Fels | OxA-19780 | 31,380 | 180 | 35,830 | 450 | Bone | Vb | AMS | Conard 2009 |
| Hohle Fels | OxA-19779 | 34,720 | 280 | 39,780 | 630 | Bone | Vb | AMS | Conard 2009 |
| Hohle Fels | OxA-19782 | 32,140 | 310 | 36,750 | 310 | Bone | Vb | AMS | Conard 2009 |
| Hohle Fels | OxA-19781 | 40,000 | 500 | 43,930 | 590 | Bone | Vb | AMS | Conard 2009 |
| Riparo Mochi | OxA-19800 | 24,600 | 100 | 29,420 | 130 | Shell | D29 | AMS | Douka et al. 2012 |
| Riparo Mochi | OxA-19801 | 25,490 | 110 | 30,400 | 150 | Shell | E32 | AMS | Douka et al. 2012 |
| Riparo Mochi | OxA-19857 | 26,030 | 110 | 30,760 | 200 | Shell | F34 | AMS | Douka et al. 2012 |
| Riparo Mochi | OxA-19728 | 26,410 | 110 | 31,050 | 110 | Shell | F40 | AMS | Douka et al. 2012 |
| Riparo Mochi | OxA-20629 | 32,910 | 220 | 37,630 | 620 | Shell | F44 | AMS | Douka et al. 2012 |
| Riparo Mochi | OxA-19614 | 32,370 | 160 | 36,890 | 260 | Shell | F49 | AMS | Douka et al. 2012 |
| Riparo Mochi | OxA-20360 | 31,960 | 150 | 36,540 | 150 | Shell | G51 | AMS | Douka et al. 2012 |
| Riparo Mochi | OxA-19802 | 30,770 | 150 | 35,510 | 150 | Shell | G51 | AMS | Douka et al. 2012 |
| Riparo Mochi | OxA-20630 | 33,180 | 230 | 37,810 | 610 | Shell | G54 | AMS | Douka et al. 2012 |
| Riparo Mochi | OxA-19290 | 36,750 | 210 | 41,690 | 210 | Shell | G57 | AMS | Douka et al. 2012 |
| Riparo Mochi | OxA-19569 | 36,350 | 260 | 41,440 | 320 | Charcoal | G60 | AMS | Douka et al. 2012 |
| Riparo Mochi | OxA-19729 | 26,140 | 110 | 30,820 | 190 | Shell | H2 | AMS | Douka et al. 2012 |
| Riparo Mochi | OxA-22268 | 24,870 | 120 | 29,830 | 270 | Shell | HH1 | AMS | Douka et al. 2012 |
| Riparo Mochi | OxA-19289 | 32,330 | 150 | 36,830 | 220 | Shell | I3 | AMS | Douka et al. 2012 |
| Riparo Mochi | OxA-19730 | 34,930 | 200 | 39,970 | 600 | Shell | I5 | AMS | Douka et al. 2012 |
| Riparo Mochi | OxA-20000 | 36,320 | 270 | 41,420 | 330 | Shell | I5 | AMS | Douka et al. 2012 |
| Trou Magrite | OxA-6564 | 25,080 | 320 | 29,940 | 410 | Bone point |  |  | Charles et al. 2003; Dinnis 2012 |
| Trou Magrite | GX-18538G | 30,100 | 2200 | 34,920 | 3020 | Bone | 2 |  | Straus 1995; Dinnis 2012 |
| Trou Magrite | GX-18537G | 34,225 | 1925 | 38,730 | 2620 | Bone | 2 |  | Straus 1995; Dinnis 2012 |
| Grotte Chauvet | GifA 101454 | 27,130 | 490 | 31,680 | 510 | Charcoal | Cave painting | AMS | Combier and Jouve 2012 |
| Grotte Chauvet | GifA-95127 | 26,120 | 400 | 30,760 | 360 | Charcoal | Cave painting | AMS | Combier and Jouve 2012 |
| Grotte Chauvet | GifA-95129 | 26,980 | 410 | 31,390 | 310 | Charcoal | Torch rubbing | AMS | Combier and Jouve 2012 |
| Grotte Chauvet | GifA-95130 | 26,980 | 420 | 31,400 | 320 | Charcoal | Torch rubbing | AMS | Combier and Jouve 2012 |
| Grotte Chauvet | GifA-101453 | 26,160 | 260 | 30,790 | 280 | Charcoal | Torch rubbing | AMS | Combier and Jouve 2012 |
| Grotte Chauvet | GifA-95126 | 30,940 | 610 | 35,600 | 710 | Charcoal | Cave painting | AMS | Combier and Jouve 2012 |
| Grotte Chauvet | GifA-95132 | 32,410 | 720 | 36,980 | 1220 | Charcoal | Cave painting | AMS | Combier and Jouve 2012 |
| Grotte Chauvet | GifA-95133 | 30,790 | 600 | 35,540 | 690 | Charcoal | Cave painting | AMS | Combier and Jouve 2012 |
| Grotte Chauvet | GifA-98157 | 20,790 | 340 | 24,890 | 660 | Charcoal | Cave painting | AMS | Combier and Jouve 2012 |
| Grotte Chauvet | GifA-96065 | 30,230 | 530 | 34,760 | 810 | Charcoal | Cave painting | AMS | Combier and Jouve 2012 |
| Grotte Chauvet | GifA-95128 | 30,340 | 570 | 34,970 | 920 | Charcoal | Cave painting | AMS | Combier and Jouve 2012 |
| Grotte Chauvet | GifA-96063 | 31,350 | 620 | 35,820 | 760 | Charcoal | Cave painting | AMS | Combier and Jouve 2012 |
| L'Arbreda | OxA-19935 | 30,950 | 220 | 36,090 | 300 | Charcoal | G | AMS | Maroto et al. 2012 |
| L'Arbreda | OxA-3729 | 37,340 | 1000 | 42,030 | 620 | Bone | H | AMS | Soler Subils et al. 2008 |
| L'Arbreda | AA-3779 | 37,700 | 1000 | 42,260 | 610 | Charcoal | H | AMS | Soler Subils et al. 2008 |
| L'Arbreda | AA-3780 | 37,700 | 1000 | 42,260 | 610 | Charcoal | H | AMS | Soler Subils et al. 2008 |
| L'Arbreda | AA-3782 | 38,700 | 1200 | 42,910 | 790 | Charcoal | H | AMS | Soler Subils et al. 2008 |
| L'Arbreda | AA-3781 | 39,900 | 1300 | 43,750 | 980 | Charcoal | H | AMS | Soler Subils et al. 2008 |
| L'Arbreda | OxA-3730 | 35,480 | 820 | 40,290 | 1090 | Bone | H | AMS | Soler Subils et al. 2008 |
| L'Arbreda | OxA-21667 | 32,250 | 450 | 36,760 | 870 | Bone | G | AMS ultrafiltration | Wood et al. 2014 |
| L'Arbreda | OxA-21783 | 32,100 | 450 | 36,490 | 770 | Bone | G | AMS ultrafiltration | Wood et al. 2014 |
| L'Arbreda | OxA-21666 | 32,750 | 450 | 37,570 | 750 | Bone | G | AMS ultrafiltration | Wood et al. 2014 |
| L'Arbreda | SANU-29018 | 32,100 | 540 | 36,610 | 950 | Bone | H | AMS ultrafiltration | Wood et al. 2014 |
| L'Arbreda | SANU-29017 | 34,800 | 760 | 39,740 | 1180 | Bone | H | AMS ultrafiltration | Wood et al. 2014 |
| L'Arbreda | SANU-29019 | 35,900 | 860 | 40,600 | 1150 | Bone | H | AMS ultrafiltration | Wood et al. 2014 |
| L'Arbreda | SANU-29016 | 35,700 | 830 | 40,470 | 1120 | Bone | H | AMS ultrafiltration | Wood et al. 2014 |
| L'Arbreda | SANU-29014 | 31,900 | 530 | 36,310 | 830 | Bone | H | AMS ultrafiltration | Wood et al. 2014 |
| L'Arbreda | OxA-21674 | 33,800 | 550 | 38,580 | 1110 | Bone | H | AMS ultrafiltration | Wood et al. 2014 |
| L'Arbreda | OxA-21665 | 35,850 | 700 | 40,600 | 990 | Bone | H | AMS ultrafiltration | Wood et al. 2014 |
| L'Arbreda | OxA-21784 | 36,000 | 700 | 40,770 | 940 | Bone | H | AMS ultrafiltration | Wood et al. 2014 |
| L'Arbreda | OxA-21664 | 35,900 | 650 | 40,710 | 900 | Bone | H | AMS ultrafiltration | Wood et al. 2014 |
| Kent's Cavern | OxA-13921 | 36,040 | 330 | 41,160 | 430 | Bone | above KC4 maxilla | AMS ultrafiltration | Higham et al. 2011 |
| Kent's Cavern | OxA-14210 | 36,370 | 320 | 41,450 | 360 | Bone | above KC4 maxilla | AMS ultrafiltration | Higham et al. 2011 |
| Kent's Cavern | OxA-14701 | 35,650 | 330 | 40,800 | 490 | Bone | above KC4 maxilla | AMS ultrafiltration | Higham et al. 2011 |
| Kent's Cavern | OxA-14059 | 35,600 | 700 | 40,420 | 1020 | Bone | above KC4 maxilla | AMS ultrafiltration | Higham et al. 2011 |
| Kent's Cavern | OxA-13965 | 37,200 | 550 | 41,990 | 540 | Bone | above KC4 maxilla | AMS ultrafiltration | Higham et al. 2011 |
| Kent's Cavern | OxA-14715 | 35,150 | 330 | 40,130 | 690 | Bone | below KC4 maxilla | AMS ultrafiltration | Higham et al. 2011 |
| Kent's Cavern | OxA-14285 | 43,600 | 3600 | 46,120 | 3160 | Bone | below KC4 maxilla | AMS ultrafiltration | Higham et al. 2011 |
| Kent's Cavern | OxA-14761 | 45,000 | 2200 | 47,220 | 1910 | Bone | below KC4 maxilla | AMS ultrafiltration | Higham et al. 2011 |
| Kent's Cavern | OxA-13888 | 40,000 | 700 | 43,940 | 740 | Bone | below KC4 maxilla | AMS ultrafiltration | Higham et al. 2011 |
| Goat's Hole (Paviland) | OxA-16412 | 28,870 | 180 | 33,580 | 490 | Bone | Red Lady | AMS ultrafiltration | Jacobi and Higham 2008; Dinnis 2012 |
| Goat's Hole (Paviland) | OxA-16413 | 29,490 | 210 | 34,050 | 430 | Bone | Red Lady | AMS ultrafiltration | Jacobi and Higham 2008; Dinnis 2012 |
| El Castillo | OxA-2473 | 37,100 | 2200 | 40,950 | 2090 | Charcoal | 18b | AMS | Cabrera Valdés et al. 2001; Soto-Barreiro 2003 |
| El Castillo | AA-2407 | 37,700 | 1800 | 41,700 | 1640 | Charcoal | 18b | AMS | Cabrera Valdés et al. 2001; Zilhão 2006 |
| El Castillo | AA-2406 | 38,500 | 1800 | 42,770 | 1250 | Charcoal | 18b | AMS | Cabrera Valdés et al. 2001; Zilhão 2006 |
| El Castillo | OxA-2474 | 38,500 | 1300 | 42,790 | 840 | Charcoal | 18b | AMS | Cabrera Valdés et al. 2001; Zilhão 2006 |
| El Castillo | GifA-49147 | 39,500 | 2000 | 43,610 | 1460 | Charcoal | 18 | AMS | Zilhão 2006 |
| El Castillo | OxA-10187 | 42,900 | 1400 | 46,150 | 1500 | Bone | 18 | AMS | Zilhão 2006 |
| El Castillo | OxA-2478 | 39,800 | 1400 | 43,710 | 1040 | Charcoal | 18c | AMS | Cabrera Valdés et al. 2001; Zilhão 2006 |
| El Castillo | AA-2405 | 40,000 | 2100 | 44,030 | 1580 | Charcoal | 18c | AMS | Cabrera Valdés et al. 2001; Zilhão 2006 |
| El Castillo | OxA-2476 | 40,700 | 1500 | 44,360 | 1200 | Charcoal | 18c | AMS | Cabrera Valdés et al. 2001; Zilhão 2006 |
| El Castillo | OxA-2475 | 40,700 | 1600 | 44,390 | 1270 | Charcoal | 18b | AMS | Cabrera Valdés et al. 2001; Zilhão 2006 |
| El Castillo | OxA-2477 | 41,100 | 1700 | 44,720 | 1390 | Charcoal | 18c | AMS | Cabrera Valdés et al. 2001; Zilhão 2006 |
| El Castillo | GifA-89147 | 42,200 | 2100 | 45,840 | 1980 | Bone | 18c | AMS | Cabrera Valdés et al. 2001; Zilhão 2006 |

**S2** Published AMS^14^C-dates for Early Upper Paleolithic sites on the Iberian Peninsula mapped in **Fig. 10a**. Dates have been calibrated with CalPal using the Intcal09 calibration curve (alphabetical site-order).

| **Site** | **Lab code** | **14C age** | **14C std** | **calBP** | **calBP std** | **Material** | **Layer** | **Method** | **References** |
| --- | --- | --- | --- | --- | --- | --- | --- | --- | --- |
| Abric Romani | NzA-1818 | 23160 | 490 | 27790 | 650 | Charcoal | 2 | AMS | Vaquero 1997; Zilhão 2006 |
| Abric Romani | NzA-1817 | 28440 | 650 | 32970 | 630 | Charcoal | 2 | AMS | Vaquero 1997; Zilhão 2006 |
| Abric Romani | AA-8037A | 35400 | 810 | 40240 | 1080 | Charcoal | 2 | AMS | Zilhão 2006 |
| Abric Romani | NzA-2311 | 36590 | 640 | 41650 | 460 | Charcoal | 2 | AMS | Vaquero 1997; Zilhão 2006 |
| Abric Romani | AA-6608 | 36740 | 920 | 41540 | 730 | Charcoal | 2 | AMS | Zilhão 2006 |
| Abric Romani | AA-7395 | 37290 | 990 | 42000 | 610 | Charcoal | 2 | AMS | Zilhão 2006 |
| Abric Romani | AA-8037B | 37900 | 1000 | 42380 | 610 | Charcoal | 2 | AMS | Zilhão 2006 |
| Abrigo del Cuco | GrA-32436 | 30020 | 160 | 35350 | 230 | Bone | XIII | AMS | Maroto et al. 2012 |
| Abrigo de Sopeña | Beta-171157 | 32870 | 530 | 37220 | 900 | Bone | XI | AMS | Pinto Llona et al. 2006 |
| Abrigo de Sopeña | GrA-39760 | 34470 | 650 | 40300 | 880 | Bone | XI | AMS | Maroto et al. 2012 |
| Beneito | AA-1388 | 33900 | 1100 | 38660 | 660 | Charcoal | C4 | AMS | Iturbe and Cortell 1987; 1992; Zilhão 2006 |
| Cobrante | GrA-22441 | 30480 | 250 | 34632 | 262 | Bone | 5 | AMS | Rasines del Río 2005; 2009 |
| Cobrante | GrA-22442 | 33320 | 310 | 37850 | 757 | Bone | 6 | AMS | Rasines del Río 2005; 2009 |
| Conde (Forno) | Beta-217216 | 32530 | 440 | 36940 | 880 | Bone | N20C | AMS | Uzquiano Ollero et al. 2008 |
| Conde (Forno) | Beta-224303 | 34730 | 500 | 39910 | 900 | Bone | N20B | AMS | Uzquiano Ollero et al. 2008 |
| Cova Gran | Beta-207576 | 21690 | 120 | 25870 | 190 | Shell | 497A | AMS | Martínez-Moreno et al. 2010 |
| Cova Gran | Beta-207577 | 26220 | 220 | 31090 | 320 | Shell | 497C | AMS | Martínez-Moreno et al. 2010 |
| Cova Gran | Beta-207578 | 32630 | 450 | 37020 | 860 | Charcoal | 497D | AMS | Martínez-Moreno et al. 2010 |
| Cova Gran | AA-68834-ABOX | 34179 | 247 | 39650 | 900 | Charcoal | 497D | AMS-ABOX | Martínez-Moreno et al. 2010 |
| Covalejos | GrA-33877 | 37940 | 400 | 42700 | 370 | Tooth | C | AMS | Maroto et al. 2012 |
| El Bajondillo | Ua-18050 | 32770 | 1065 | 37460 | 1530 | Charcoal Sediment | 11 | AMS | Cortés Sánchez 2007 |
| El Bajondillo | Ua-17150 | 33690 | 1195 | 38490 | 1860 | Charcoal Sediment | 11 | AMS | Cortés Sánchez 2007 |
| El Castillo | OxA-2473 | 37100 | 2200 | 40950 | 2090 | Charcoal | 18b | AMS | Cabrera Valdés et al. 2001; Soto-Barreiro 2003 |
| El Castillo | AA-2407 | 37700 | 1800 | 41700 | 1640 | Charcoal | 18b | AMS | Cabrera Valdés et al. 2001; Zilhão 2006 |
| El Castillo | AA-2406 | 38500 | 1800 | 42770 | 1250 | Charcoal | 18b | AMS | Cabrera Valdés et al. 2001; Zilhão 2006 |
| El Castillo | OxA-2474 | 38500 | 1300 | 42790 | 840 | Charcoal | 18b | AMS | Cabrera Valdés et al. 2001; Zilhão 2006 |
| El Castillo | GifA-49147 | 39500 | 2000 | 43610 | 1460 | Charcoal | 18 | AMS | Zilhão 2006 |
| El Castillo | OxA-10187 | 42900 | 1400 | 46150 | 1500 | Bone | 18 | AMS | Zilhão 2006 |
| El Castillo | OxA-2478 | 39800 | 1400 | 43710 | 1040 | Charcoal | 18c | AMS | Cabrera Valdés et al. 2001; Zilhão 2006 |
| El Castillo | AA-2405 | 40000 | 2100 | 44030 | 1580 | Charcoal | 18c | AMS | Cabrera Valdés et al. 2001; Zilhão 2006 |
| El Castillo | OxA-2476 | 40700 | 1500 | 44360 | 1200 | Charcoal | 18c | AMS | Cabrera Valdés et al. 2001; Zilhão 2006 |
| El Castillo | OxA-2475 | 40700 | 1600 | 44390 | 1270 | Charcoal | 18b | AMS | Cabrera Valdés et al. 2001; Zilhão 2006 |
| El Castillo | OxA-2477 | 41100 | 1700 | 44720 | 1390 | Charcoal | 18c | AMS | Cabrera Valdés et al. 2001; Zilhão 2006 |
| El Castillo | GifA-89147 | 42200 | 2100 | 45840 | 1980 | Bone | 18c | AMS | Cabrera Valdés et al. 2001; Zilhão 2006 |
| Foradá | Beta-132350 | 26610 | 460 | 31300 | 440 | Charcoal | II | AMS | Zilhão 2006 |
| Foradá | Beta-132351 | 28300 | 170 | 32730 | 270 | Charcoal | II | AMS | Zilhão 2006 |
| Foradá | Beta-103782 | 27190 | 150 | 31870 | 120 | Charcoal | V | AMS | Zilhão 2006 |
| Foradá | Beta-103781 | 29440 | 190 | 33840 | 270 | Charcoal | V | AMS | Zilhão 2006 |
| Foradá | Beta-103783 | 29950 | 200 | 34250 | 180 | Charcoal | VI | AMS | Zilhão 2006 |
| Foradá | Beta-132349 | 33900 | 310 | 39494 | 1012 | Charcoal | VII | AMS | Zilhão 2006 |
| Gorham's Cave | OxA-6997 | 25680 | 280 | 30610 | 380 | Bone | CON 7 | AMS | Finlayson et al. 2006 |
| Gorham's Cave | OxA-7792 | 28680 | 240 | 33160 | 350 | Charcoal | CON 7 | AMS | Pettitt et al. 2000; Zilhão 2006 |
| Gorham's Cave | OxA-7077 | 29250 | 650 | 33550 | 590 | Charcoal | CON 9 | AMS | Pettitt et al. 2000; Zilhão 2006 |
| Gorham's Cave | OxA-7075 | 29800 | 700 | 33990 | 610 | Charcoal | CON 9 | AMS | Pettitt et al. 2000; Zilhão 2006 |
| Gorham's Cave | OxA-7074 | 30200 | 700 | 34410 | 620 | Charcoal | CON 9 | AMS | Pettitt et al. 2000; Zilhão 2006 |
| Gorham's Cave | OxA-7076 | 30250 | 700 | 34460 | 620 | Charcoal | CON 9 | AMS | Pettitt et al. 2000; Zilhão 2006 |
| Gorham's Cave | OxA-7388 | 2910 | 340 | 3060 | 410 | Bone | CON 11 | AMS | Zilhão 2006 |
| Gorham's Cave | OxA-7110 | 29250 | 750 | 33530 | 660 | Charcoal | CON 13a | AMS | Pettitt et al. 2000; Zilhão 2006 |
| Labeko Koba | Ua-3035 | 23365 | 350 | 28220 | 380 | Bone | IV middle | AMS | Arrizabalaga 2000 |
| Labeko Koba | Ua-3322 | 30615 | 820 | 34820 | 690 | Bone | IV middle | AMS | Arrizabalaga 2000 |
| Labeko Koba | Ua-3321 | 31450 | 915 | 35970 | 1210 | Bone | VII upper | AMS | Arrizabalaga 2000 |
| Labeko Koba | Ua-3320 | 26910 | 500 | 31550 | 440 | Bone | VII lower | AMS | Arrizabalaga 2000 |
| Labeko Koba | OxA-21768 | 33600 | 500 | 38210 | 920 | Bone | IV | AMS ultrafiltration | Wood et al. 2014 |
| Labeko Koba | OxA-21780 | 33550 | 550 | 38200 | 980 | Bone | IV | AMS ultrafiltration | Wood et al. 2014 |
| Labeko Koba | OxA-21779 | 34650 | 600 | 39820 | 900 | Bone | V | AMS ultrafiltration | Wood et al. 2014 |
| Labeko Koba | OxA-21767 | 34750 | 600 | 39880 | 910 | Bone | V | AMS ultrafiltration | Wood et al. 2014 |
| Labeko Koba | OxA-21794 | 32200 | 450 | 36660 | 820 | Bone | VI | AMS ultrafiltration | Wood et al. 2014 |
| Labeko Koba | OxA-21841 | 32150 | 450 | 36580 | 780 | Bone | VI | AMS ultrafiltration | Wood et al. 2014 |
| Labeko Koba | OxA-21778 | 35100 | 600 | 40110 | 930 | Bone | VI | AMS ultrafiltration | Wood et al. 2014 |
| Labeko Koba | OxA-21793 | 35400 | 650 | 40300 | 980 | Bone | VII | AMS ultrafiltration | Wood et al. 2014 |
| Labeko Koba | OxA-21840 | 35250 | 650 | 40210 | 980 | Bone | VII | AMS ultrafiltration | Wood et al. 2014 |
| Labeko Koba | OxA-X-2314-43 | 36500 | 750 | 41370 | 820 | Bone | VII | AMS ultrafiltration | Wood et al. 2014 |
| Labeko Koba | OxA-21766 | 36850 | 800 | 41660 | 820 | Bone | VII | AMS ultrafiltration | Wood et al. 2014 |
| L'Arbreda | OxA-19935 | 30950 | 220 | 36090 | 300 | Charcoal | G | AMS | Maroto et al. 2012 |
| L'Arbreda | OxA-3729 | 37340 | 1000 | 42030 | 620 | Bone | H | AMS | Soler Subils et al. 2008 |
| L'Arbreda | AA-3779 | 37700 | 1000 | 42260 | 610 | Charcoal | H | AMS | Soler Subils et al. 2008 |
| L'Arbreda | AA-3780 | 37700 | 1000 | 42260 | 610 | Charcoal | H | AMS | Soler Subils et al. 2008 |
| L'Arbreda | AA-3782 | 38700 | 1200 | 42910 | 790 | Charcoal | H | AMS | Soler Subils et al. 2008 |
| L'Arbreda | AA-3781 | 39900 | 1300 | 43750 | 980 | Charcoal | H | AMS | Soler Subils et al. 2008 |
| L'Arbreda | OxA-3730 | 35480 | 820 | 40290 | 1090 | Bone | H | AMS | Soler Subils et al. 2008 |
| L'Arbreda | OxA-21667 | 32250 | 450 | 36760 | 870 | Bone | G | AMS ultrafiltration | Wood et al. 2014 |
| L'Arbreda | OxA-21783 | 32100 | 450 | 36490 | 770 | Bone | G | AMS ultrafiltration | Wood et al. 2014 |
| L'Arbreda | OxA-21666 | 32750 | 450 | 37570 | 750 | Bone | G | AMS ultrafiltration | Wood et al. 2014 |
| L'Arbreda | SANU-29018 | 32100 | 540 | 36610 | 950 | Bone | H | AMS ultrafiltration | Wood et al. 2014 |
| L'Arbreda | SANU-29017 | 34800 | 760 | 39740 | 1180 | Bone | H | AMS ultrafiltration | Wood et al. 2014 |
| L'Arbreda | SANU-29019 | 35900 | 860 | 40600 | 1150 | Bone | H | AMS ultrafiltration | Wood et al. 2014 |
| L'Arbreda | SANU-29016 | 35700 | 830 | 40470 | 1120 | Bone | H | AMS ultrafiltration | Wood et al. 2014 |
| L'Arbreda | SANU-29014 | 31900 | 530 | 36310 | 830 | Bone | H | AMS ultrafiltration | Wood et al. 2014 |
| L'Arbreda | OxA-21674 | 33800 | 550 | 38580 | 1110 | Bone | H | AMS ultrafiltration | Wood et al. 2014 |
| L'Arbreda | OxA-21665 | 35850 | 700 | 40600 | 990 | Bone | H | AMS ultrafiltration | Wood et al. 2014 |
| L'Arbreda | OxA-21784 | 36000 | 700 | 40770 | 940 | Bone | H | AMS ultrafiltration | Wood et al. 2014 |
| L'Arbreda | OxA-21664 | 35900 | 650 | 40710 | 900 | Bone | H | AMS ultrafiltration | Wood et al. 2014 |
| La Viña | Ly-49 (OxA) | 16490 | 250 | 19770 | 350 | Bone | IX | AMS | Hedges et al. 1997; Soto-Barreiro 2003 |
| La Viña | OxA-4092 | 19930 | 220 | 23880 | 290 | Bone | XIII A | AMS | Hedges et al. 1997 |
| La Viña | GifA-95463 | 31860 | 680 | 36290 | 1120 | Charcoal | XIII-middle/upper | AMS | Zilhão 2006 |
| La Viña | OxA-19195 | 30130 | 170 | 34780 | 150 | Bone | XI | AMS | Wood et al. 2014 |
| La Viña | OxA-21687 | 30600 | 370 | 35430 | 600 | Bone | XI | AMS ultrafiltration | Wood et al. 2014 |
| La Viña | OxA-21686 | 20820 | 130 | 24800 | 260 | Bone | XI | AMS ultrafiltration | Wood et al. 2014 |
| La Viña | OxA-X-2290-19 | 27900 | 280 | 32210 | 540 | Bone | XI | AMS ultrafiltration | Wood et al. 2014 |
| La Viña | OxA-21689 | 31500 | 400 | 35880 | 580 | Bone | XII | AMS ultrafiltration | Wood et al. 2014 |
| La Viña | OxA-21678 | 31600 | 400 | 35940 | 590 | Bone | XII | AMS ultrafiltration | Wood et al. 2014 |
| La Viña | OxA-21845 | 30650 | 360 | 35450 | 580 | Bone | XIII | AMS ultrafiltration | Wood et al. 2014 |
| La Viña | OxA-21705 | 31160 | 380 | 35700 | 570 | Bone | XIII | AMS ultrafiltration | Wood et al. 2014 |
| Lezetxiki | OxA-22021 | 29250 | 320 | 34440 | 500 | Bone | III | AMS | Maroto et al. 2012 |
| Lezetxiki | OxA-21838 | 30830 | 380 | 35980 | 390 | Bone | III | AMS | Maroto et al. 2012 |
| Lezetxiki | OxA-21837 | 34550 | 100 | 40420 | 690 | Bone | III | AMS | Maroto et al. 2012 |
| Mollet I | OxA-3728 | 33780 | 730 | 38690 | 1660 | Bone | 0.60-0.80 m | AMS | Maroto 1994 |
| Morín | OxA-19084 | 40060 | 350 | 43900 | 480 | Charcoal | 8 | AMS | Maroto et al. 2012 |
| Morín | GrA-33891 | 33430 | 250 | 38440 | 850 | Charcoal | 9 | AMS | Maroto et al. 2012 |
| Pego do Diabo | VERA-4048B | 27290 | 230 | 31940 | 170 | Bone | 2 (spit 2b) | AMS | Zilhão et al. 2010 |
| Pego do Diabo | VERA-4048C | 27740 | 230 | 32280 | 250 | Bone | 2 (spit 2b) | AMS | Zilhão et al. 2010 |
| Pego do Diabo | VERA-4048UF1B | 28040 | 250 | 32520 | 300 | Bone | 2 (spit 2b) | AMS | Zilhão et al. 2010 |
| Pego do Diabo | VERA-4048 | 28320 | 280 | 32760 | 350 | Bone | 2 (spit 2b) | AMS | Zilhão et al. 2010 |
| Pego do Diabo | VERA-4047UF2B | 28360 | 270 | 32800 | 340 | Bone | 2 (spit 2b) | AMS | Zilhão et al. 2010 |
| Pego do Diabo | VERA-4047 | 29090 | 270 | 33550 | 350 | Bone | 2 (spit 2b) | AMS | Zilhão et al. 2010 |
| Pego do Diabo | VERA-4047UF1B | 29150 | 280 | 33590 | 350 | Bone | 2 (spit 2b) | AMS | Zilhão et al. 2010 |
| Reclau Viver | OxA-3726 | 30190 | 500 | 34420 | 410 | Bone | B | AMS | Maroto et al. 1996; Zilhão 2006 |
| Reclau Viver | OxA-3727 | 40000 | 1400 | 43840 | 1060 | Bone | A | AMS | Maroto et al. 1996; Zilhão 2006 |

**S3** Published AMS^14^C-dates for Middle Upper Paleolithic sites on the Iberian Peninsula mapped in **Fig. 10b**. Dates have been calibrated with CalPal using the Intcal09 calibration curve (alphabetical site-order).

| **Site** | **Lab code** | **14C age** | **14C std** | **calBP** | **calBP std** | **Material** | **Layer** | **Method** | **References** |
| --- | --- | --- | --- | --- | --- | --- | --- | --- | --- |
| Abrigo del Cuco | GrNA-32097 | 23400 | 210 | 28180 | 150 | Bone | III | AMS | Peña Alonso 2009 |
| Aitzbitarte III | Ua | 27580 | 550 | 32270 | 460 | Bone | IV | AMS | Altuna 2003 |
| Aitzbitarte III | Ua | 28320 | 605 | 32870 | 600 | Bone | IV | AMS | Altuna 2003 |
| Aitzbitarte III | Ua | 28950 | 655 | 33320 | 630 | Bone | IV | AMS | Altuna 2003 |
| Aitzbitarte III | Ua-2243 | 23230 | 330 | 27940 | 360 | Bone | V | AMS | Soto-Barreiro 2003 |
| Aitzbitarte III | Ua-2626 | 24545 | 415 | 29280 | 520 | Bone | V | AMS | Soto-Barreiro 2003 |
| Aitzbitarte III | Ua-1917 | 21130 | 290 | 25240 | 360 | Bone | VI | AMS | Soto-Barreiro 2003; Arrizabalaga 2009; Peña Alonso 2009 |
| Aitzbitarte III | Ua-2628 | 23830 | 345 | 28790 | 470 | Bone | VI | AMS | Soto-Barreiro 2003 |
| Aitzbitarte III | Ua-2627 | 24635 | 475 | 29370 | 580 | Bone | VI | AMS | Soto-Barreiro 2003 |
| Aitzbitarte III | Ua-2245 | 24920 | 410 | 29790 | 430 | Bone | VI | AMS | Soto-Barreiro 2003 |
| Aitzbitarte III | Ua-2244 | 25380 | 430 | 30370 | 440 | Bone | VI | AMS | Soto-Barreiro 2003 |
| Aitzbitarte III | Ua-24964 | 19230 | 200 | 22950 | 400 |  | III | AMS | Altuna et al. 2013 |
| Aitzbitarte III | Ua-24963 | 22580 | 295 | 27120 | 610 |  | III | AMS | Altuna et al. 2013 |
| Aitzbitarte III | Ua-11148 | 25815 | 475 | 30380 | 570 |  | III | AMS | Altuna et al. 2013 |
| Aitzbitarte III | Ua-11146 | 24240 | 365 | 28980 | 480 |  | III | AMS | Altuna et al. 2013 |
| Aitzbitarte III | Ua-37961 | 26260 | 480 | 30830 | 400 |  | III | AMS | Altuna et al. 2013 |
| Aitzbitarte III | Ua-18464 | 27580 | 550 | 32140 | 730 |  | III | AMS | Altuna et al. 2013 |
| Aitzbitarte III | Ua-24965 | 22420 | 290 | 27010 | 620 |  | IV | AMS | Altuna et al. 2013 |
| Aitzbitarte III | Ua-18465 | 28320 | 605 | 32930 | 1090 |  | IV | AMS | Altuna et al. 2013 |
| Aitzbitarte III | Ua-24967 | 26350 | 475 | 30860 | 400 |  | IV | AMS | Altuna et al. 2013 |
| Aitzbitarte III | Ua-24966 | 27165 | 520 | 31770 | 580 |  | IV | AMS | Altuna et al. 2013 |
| Aitzbitarte III | Ua-37962 | 28530 | 645 | 33000 | 1100 |  | IV | AMS | Altuna et al. 2013 |
| Aitzbitarte III | Ua-18466 | 28950 | 655 | 33200 | 1110 |  | Va | AMS | Altuna et al. 2013 |
| Aitzbitarte III | Ua-18467 | 31210 | 860 | 35980 | 1010 |  | Va | AMS | Altuna et al. 2013 |
| Buraca Escura | OxA-5524 | 21820 | 200 | 26160 | 370 | Bone | C2a | AMS | Aubry et al. 2001 |
| Buraca Escura | OxA-5523 | 22700 | 240 | 27410 | 380 | Bone | C2e | AMS | Aubry et al. 2001 |
| Buraca Grande | GifA-93048 | 23920 | 300 | 28850 | 440 | Charcoal | 9B | AMS | Aubry et al. 1997 |
| Casa da Moura | TO-1102 | 25900 | 220 | 30830 | 340 | Bone | 1b | AMS | Straus et al. 1988 |
| Cova de les Cendres | Beta-142282 | 21230 | 180 | 25300 | 270 | Charcoal | XIV | AMS | Villaverde Bonilla and Roman 2004 |
| Cova de les Cendres | Beta-155606 | 24080 | 150 | 28960 | 350 | Charcoal | XVIA | AMS | Villaverde Bonilla and Roman 2004 |
| Cova de les Cendres | Beta-142283 | 24240 | 220 | 29090 | 400 | Charcoal | XVIA | AMS | Villaverde Bonilla and Roman 2004 |
| Cova de les Cendres | Beta-189078 | 25850 | 260 | 30790 | 370 | Charcoal | XVIC | AMS | Villaverde Bonilla and Roman 2004 |
| Cueto de la Mina | Ua-3587 | 26470 | 520 | 31180 | 490 | Bone | H | AMS | Soto-Barreiro 2003 |
| El Castillo | Beta 242617 | 24070 | 150 | 28940 | 330 | Bone | 12 | AMS | Bernaldo de Quirós et al. 2013 |
| El Castillo | Beta 298431 | 25520 | 140 | 30450 | 190 | Bone | 12 | AMS | Bernaldo de Quirós et al. 2013 |
| El Castillo | Beta 298430 | 25920 | 140 | 30690 | 230 | Bone | 12 | AMS | Bernaldo de Quirós et al. 2013 |
| El Castillo | Beta 298433 | 29600 | 180 | 34140 | 390 | Bone | 14 | AMS | Bernaldo de Quirós et al. 2013 |
| El Castillo | Beta 298432 | 29740 | 190 | 34390 | 270 | Bone | 14 | AMS | Bernaldo de Quirós et al. 2013 |
| El Mirón | GX-27113 | 27580 | 210 | 32140 | 200 | Charcoal | 128 | AMS | Peña Alonso 2009 |
| El Palomar | Beta-185410 | 26430 | 210 | 31270 | 290 | Bone | IV | AMS | Vega and Martín 2006 |
| Labeko Koba | Ua-3323 | 21665 | 305 | 25880 | 540 | Bone | IV middle | AMS | Arrizabalaga 2000 |
| Lagar Velho | Sac-1561 | 21380 | 810 | 25730 | 1050 | Charcoal | 6 (Q.Z west) | AMS | Duarte et al. 1999; Zilhão and Trinkaus 2000 |
| Lagar Velho | OxA-8418 | 22180 | 180 | 26610 | 340 | Charcoal | 6 (Q.A-190 cm) | AMS | Duarte et al. 1999; Zilhão and Trinkaus 2000 |
| Lagar Velho | OxA-9571 | 23130 | 130 | 27990 | 100 | Bone | GC 3 | AMS | Zilhão and Trinkaus 2000; Aubry et al. 2011 |
| Lagar Velho | OxA-9572 | 23170 | 140 | 28020 | 100 | Bone | GC 3 | AMS | Zilhão and Trinkaus 2000; Aubry et al. 2011 |
| Lagar Velho | Beta-139361 | 22720 | 90 | 27450 | 320 | Bone | GC 4 | AMS | Zilhão and Trinkaus 2000; Aubry et al. 2011 |
| Lagar Velho | OxA-10674 | 24950 | 230 | 29920 | 190 | Bone | GC 5 | AMS | Zilhão and Trinkaus 2000; Aubry et al. 2011 |
| Lagar Velho | OxA-8422 | 23920 | 220 | 28830 | 400 | Bone | gs top | AMS | Duarte et al. 1999; Zilhão and Trinkaus 2000 |
| Lagar Velho | OxA-8423 | 24520 | 240 | 29340 | 380 | Bone | gs top | AMS | Duarte et al. 1999; Zilhão and Trinkaus 2000 |
| Lagar Velho | OxA-8421 | 24660 | 260 | 29510 | 320 | Bone | gs top | AMS | Duarte et al. 1999; Zilhão and Trinkaus 2000 |
| Lagar Velho | GrA-13310 | 24860 | 200 | 29840 | 150 | Charcoal | gs top | AMS | Duarte et al. 1999; Zilhão and Trinkaus 2000 |
| Lagar Velho | Wk-9571 | 23042 | 142 | 27920 | 130 | Bone | ls/ms boundary | AMS | Zilhão and Trinkaus 2000 |
| Lagar Velho | WK-9256 | 22493 | 107 | 27270 | 300 | Charcoal | ms | AMS | Zilhão and Trinkaus 2000 |
| Lagar Velho | OxA-8426 | 20570 | 130 | 24590 | 130 | Charcoal | us | AMS | Zilhão and Trinkaus 2000 |
| Lagar Velho | OxA-8424 | 22300 | 300 | 26900 | 580 | Charcoal | us | AMS | Zilhão and Trinkaus 2000 |
| Lagar Velho | OxA-8425 | 22670 | 160 | 27400 | 330 | Charcoal | us | AMS | Zilhão and Trinkaus 2000 |
| L'Arbreda | OxA-21669 | 25780 | 210 | 30630 | 270 | Bone | E | AMS ultrafiltration | Wood et al. 2014 |
| L'Arbreda | OxA-21668 | 26100 | 210 | 30770 | 250 | Bone | E | AMS ultrafiltration | Wood et al. 2014 |
| L'Arbreda | OxA-21782 | 28280 | 290 | 32480 | 610 | Bone | F | AMS ultrafiltration | Wood et al. 2014 |
| L'Arbreda | OxA-21781 | 28260 | 280 | 32470 | 600 | Bone | F | AMS ultrafiltration | Wood et al. 2014 |
| La Viña | OxA-21688 | 24640 | 190 | 31110 | 170 | Bone | IX | AMS ultrafiltration | Wood et al. 2014 |
| Les Mallaetes | Beta | 25120 | 240 | 30050 | 200 | Charcoal | Level 12 (sector E) | AMS | Arsuaga et al. 2002 |
| Nerja | Beta-131576 | 24480 | 110 | 29510 | 170 | Charcoal | 13 | AMS | Aura Tortosa et al. 1998; 2006 |
| Nerja | Beta-189080 | 24200 | 200 | 29060 | 380 | Charcoal | 12 | AMS | Aura Tortosa et al. 1998; 2006 |

**S4** Published AMS^14^C-dates for Magdalenian and Late Upper Paleolithic sites in the Ebro valley mapped in **Fig. 11c**. Dates have been calibrated with CalPal using the Intcal09 calibration curve (alphabetical site-order).

| **Site** | **Lab code** | **14C age** | **14C std** | **calBP** | **calBP std** | **Material** | **Layer** | **Method** | **References** |
| --- | --- | --- | --- | --- | --- | --- | --- | --- | --- |
| Abauntz | GrA-39336 | 12,220 | 60 | 14,310 | 220 | Harpoon |  | AMS | Utrilla and Mazo unpublished data; Utrilla et al. 2012 |
| Alonsé | GrA-21536 | 15,069 | 90 | 18,240 | 230 | Charcoal | m | AMS | Montes 2005 |
| Alonsé | GrA-21537 | 14,840 | 90 | 18,120 | 250 | Charcoal | m | AMS | Montes 2005 |
| Atxoste | GrA-19503 | 12,540 | 80 | 14,980 | 150 | Bone | h2 | AMS | Barandiarán et al. 2006 |
| Atxoste | GrA-19502 | 12,200 | 90 | 14,300 | 240 | Bone | g | AMS | Barandiarán et al. 2006 |
| Atxoste | GrA-19554 | 12,070 | 60 | 14,020 | 160 | Bone | f2 | AMS | Barandiarán et al. 2006 |
| Atxoste | GrA-22866 | 11,760 | 70 | 13,670 | 70 | Bone | VIIc | AMS | Barandiarán et al. 2006 |
| Atxoste | GrA-19870 | 11,730 | 80 | 13,630 | 90 | Bone | h | AMS | Barandiarán et al. 2006 |
| Atxoste | GrA-22865 | 11,720 | 70 | 13,630 | 90 | Bone | VIIb | AMS | Barandiarán et al. 2006 |
| Atxoste | GrA-23107 | 11,690 | 80 | 13,580 | 110 | Bone | VIIc | AMS | Barandiarán et al. 2006 |
| Colls | AA-8645 | 10,950 | 120 | 12,890 | 110 |  | II | AMS | Bergadá 1998 |
| Colls | AA-8646 | 10,050 | 85 | 11,600 | 190 |  | II | AMS | Bergadá 1998 |
| Cova Gran | Beta-233606 | 16,800 | 80 | 20,020 | 290 | Charcoal | EA-3 | AMS | Mora et al. 2011 |
| Cova Gran | Beta-265984 | 15,120 | 70 | 18,300 | 250 | Charcoal | 6P | AMS | Mora et al. 2011 |
| Cova Gran | Beta-259273 | 14,760 | 70 | 18,110 | 310 | Charcoal | 4P | AMS | Mora et al. 2011 |
| Cova Gran | Beta-187224 | 13,660 | 50 | 16,775 | 225 | Charcoal | S4H | AMS | Mora et al. 2011 |
| Gato 2 | GrA-30684 | 18,850 | 100 | 22,687 | 243 | Charcoal | II | AMS | Blasco and Rodanés 2004 |
| Gato 2 | GrA-22505 | 18,650 | 140 | 22,480 | 160 | Charcoal | II | AMS | Blasco and Rodanés 2004 |
| Gato 2 | GrA-22503 | 18,260 | 130 | 21,960 | 270 | Charcoal | II | AMS | Blasco and Rodanés 2004 |
| Gato 2 | GrA-30683 | 18,090 | 90 | 21,690 | 170 | Charcoal | II | AMS | Blasco and Rodanés 2004 |
| Gato 2 | GrA-42226 | 17,700 | 70 | 21,240 | 100 | Bone | II | AMS | Blasco and Rodanés 2004 |
| Forcas I | GrA-25979 | 14,440 | 70 | 17,680 | 70 | Bone | 15 | AMS | Utrilla and Mazo 2007 |
| Forcas I | GrA-33986 | 12,600 | 60 | 15,080 | 120 | Bone | 14 | AMS | Utrilla and Mazo 2007 |
| Forcas I | GrA-32957 | 12,440 | 50 | 14,810 | 100 | Bone | 13d | AMS | Utrilla and Mazo 2007 |
| Forcas I | GrA-33987 | 12,010 | 60 | 13,900 | 80 | Bone | 13a | AMS | Utrilla and Mazo 2007 |
| Kukuma | Ua-2625 | 11,550 | 130 | 13,440 | 130 | Bone | 11 | AMS | Baldeón and Berganzá 1997 |
| Legintxiki | Ua | 17,025 | 95 | 20,460 | 110 |  | II | AMS | Nuin 1996 |
| Legintxiki | Ua-3397 | 14,865 | 140 | 18,150 | 270 | Bone | Ia | AMS | Nuin 1996 |
| Legunova | GrA-22089 | 12,500 | 90 | 14,910 | 170 | Charcoal | q | AMS | Montes 2005 |
| Legunova | GrA-24296 | 12,060 | 60 | 14,010 | 150 | Charcoal | q | AMS | Montes 2005 |
| Legunova | GrA-22087 | 11,980 | 80 | 13,880 | 90 | Charcoal | q | AMS | Montes 2005 |
| Legunova | GrA-27843 | 11,780 | 60 | 13,680 | 70 | Charcoal | q | AMS | Montes 2005 |
| Legunova | GrA-27841 | 11,640 | 60 | 13,510 | 80 | Charcoal | q | AMS | Montes 2005 |
| Legunova | GrA-27846 | 11,240 | 60 | 13,150 | 60 | Charcoal | q | AMS | Montes 2005 |
| Legunova | GrA-24295 | 10,760 | 60 | 12,730 | 30 | Charcoal | m | AMS | Montes 2005 |
| Montlleó | OxA-14034 | 15,550 | 140 | 18,710 | 80 | Charcoal | Sector C | AMS | Mangado et al. 2009 |
| Montlleó | OxA-9017 | 15,440 | 80 | 18,650 | 50 | Bone | Sector B | AMS | Mangado et al. 2009 |
| Parco | GifA-95552 | 14,300 | 150 | 17,470 | 200 | Charcoal | XI | AMS | Bergadá 1998 |
| Parco | GifA-95542 | 14,040 | 140 | 17,270 | 180 | Charcoal | VII | AMS | Bergadá 1998 |
| Parco | AA-8644 | 13,950 | 150 | 17,170 | 160 | Charcoal | VI | AMS | Bergadá 1998 |
| Parco | GifA-95565 | 13,890 | 130 | 17,070 | 100 |  | V | AMS | Bergadá 1998 |
| Parco | OxA-10798 | 13,175 | 60 | 16,040 | 280 | Charcoal | II | AMS | Mangado et al. 2006 |
| Parco | GifA-95564 | 13,070 | 140 | 15,960 | 360 |  | III | AMS | Bergadá 1998 |
| Parco | OxA-13596 | 13,025 | 50 | 15,640 | 100 | Charcoal | II | AMS | Mangado et al. 2006 |
| Parco | OxA-13597 | 12,995 | 50 | 15,570 | 60 | Charcoal | II | AMS | Mangado et al. 2006 |
| Parco | AA-8643 | 12,900 | 130 | 15,540 | 230 | Charcoal | IV | AMS | Bergadá 1998 |
| Parco | OxA-10796 | 12,605 | 60 | 14,960 | 300 | Charcoal | II | AMS | Mangado et al. 2006 |
| Parco | OxA-10835 | 12,560 | 130 | 14,990 | 230 | Charcoal | II | AMS | Mangado et al. 2006 |
| Parco | OxA-10797 | 12,460 | 60 | 14,840 | 120 | Charcoal | II | AMS | Mangado et al. 2006 |
| Parco | OxA-8656 | 11,430 | 60 | 13,330 | 70 | Charcoal | Ib | AMS | Bergadá 1998 |
| Parco | OxA-8657 | 11,270 | 90 | 13,170 | 90 | Charcoal | Ic | AMS | Bergadá 1998 |
| Parco | GifA-95562 | 10,930 | 100 | 12,880 | 90 |  | Ia inf | AMS | Bergadá 1998 |
| Parco | GifA-95563 | 10,770 | 110 | 12,760 | 80 |  | Ia inf | AMS | Bergadá 1998 |
| Parco | GifA-95543 | 10,420 | 110 | 12,330 | 200 | Charcoal | Ia sup | AMS | Bergadá 1998 |
| Parco | AA-12410 | 10,190 | 100 | 11,860 | 220 |  | Ia sup | AMS | Bergadá 1998 |
| Peña del Diablo 1 | GrN-21014 | 10,760 | 140 | 12,740 | 120 |  | 2 | AMS | Utrilla et al. 1999 |
| Urratxa III | Ua-11433 | 10,240 | 100 | 12,010 | 240 | Bone | II | AMS | Barandiarán et al. 2006 |

**S5** Central European Magdalenian sites mapped in **Fig. 12** and used for spatial analysis (ordered by country). Coordinates are given in decimal degrees. Classes: **1** ^14^C-dates *and* typology support a Magdalenian association; **2** ^14^C-dates *or* typological arguments speak in favour of a Magdalenian attribution.

| **Site** | **Layer** | **Longitude** | **Latitude** | **Country** | **Class** |
| --- | --- | --- | --- | --- | --- |
| Campalou | CA 3a | 5 14 29 | 45 03 45 | France | 1 |
| La Garenne | B5 | 5 16 34 | 45 40 00 | France | 1 |
| La Garenne | B4 | 5 16 34 | 45 40 00 | France | 1 |
| La Garenne | B3 | 5 16 34 | 45 40 00 | France | 1 |
| La Garenne | B2 | 5 16 34 | 45 40 00 | France | 1 |
| La Garenne | B1+B2 | 5 16 34 | 45 40 00 | France | 1 |
| La Garenne | A2-A3 | 5 16 34 | 45 40 00 | France | 1 |
| La Garenne | A | 5 16 34 | 45 40 00 | France | 1 |
| La Garenne | Z | 5 16 34 | 45 40 00 | France | 1 |
| Croze-sur-Suran | R7 | 5 19 41 | 46 06 07 | France | 1 |
| Calvaire, A. du | IV inf. | 5 20 12 | 45 06 31 | France | 1 |
| Calvaire, A. du | III moy. | 5 20 12 | 45 06 31 | France | 1 |
| Calvaire, A. du | II sup. | 5 20 12 | 45 06 31 | France | 1 |
| Balme d'Isère | foyer A | 5 20 17 | 45 51 04 | France | 1 |
| Colombière, La | B | 5 22 16 | 46 04 53 | France | 1 |
| Raillarde, La |  | 5 25 01 | 45 51 81 | France | 1 |
| Passagère |  | 5 31 35 | 45 07 16 | France | 1 |
| Grappin (G. St.-Vincent) |  | 5 31 37 | 46 45 46 | France | 1 |
| Colomb |  | 5 31 39 | 45 97 36 | France | 1 |
| Chênelaz | 2C / 2 | 5 31 60 | 45 53 06 | France | 1 |
| Bibi, G. (Eremitage) |  | 5 37 33 | 45 19 19 | France | 1 |
| Farincourt, G. de | I+II | 5 40 55 | 47 41 58 | France | 1 |
| Farincourt, G. de | surface | 5 40 55 | 47 41 58 | France | 1 |
| Farincourt, G. de | III-E | 5 40 55 | 47 41 58 | France | 1 |
| Farincourt, G. de | III-B | 5 40 55 | 47 41 58 | France | 1 |
| Balme de Glos | 10 to 11 | 5 40 58 | 45 11 07 | France | 1 |
| Balme de Glos |  | 5 40 58 | 45 11 07 | France | 1 |
| Romains | 3 | 5 42 45 | 45 42 54 | France | 1 |
| Romains | 2b | 5 42 45 | 45 42 54 | France | 1 |
| Romains | 2a | 5 42 45 | 45 42 54 | France | 1 |
| Seveux |  | 5 44 59 | 47 33 22 | France | 1 |
| A. de la Fru | Aire I | 5 47 07 | 45 26 48 | France | 1 |
| A. de la Fru | Aire II | 5 47 07 | 45 26 48 | France | 1 |
| Jean Pierre I | 9A+B | 5 50 41 | 45 30 02 | France | 1 |
| Jean Pierre II |  | 5 50 42 | 45 30 02 | France | 1 |
| Baume Noire | M5 | 5 56 48 | 47 29 18 | France | 1 |
| Baume Noire | M2 | 5 56 48 | 47 29 18 | France | 1 |
| Baume Noire | M3 | 5 56 48 | 47 29 18 | France | 1 |
| Baume Noire | M4 | 5 56 48 | 47 29 18 | France | 1 |
| Douattes, Les | Level 7/6 | 5 57 12 | 46 00 52 | France | 1 |
| Banges, G. du | G sup.+inf. | 6 04 49 | 45 45 06 | France | 1 |
| La Guillotine, Chariez |  | 6 05 17 | 47 37 22 | France | 1 |
| Rigney 1, G. de | D | 6 10 53 | 47 23 14 | France | 1 |
| Rigney 1, G. de | M | 6 10 53 | 47 23 14 | France | 1 |
| Veyrier |  | 6 11 20 | 45 52 31 | France | 1 |
| Auzary-Thônes |  | 6 19 48 | 45 52 08 | France | 1 |
| Roc-la-Tour | 1 | 4 46 59 | 49 53 26 | France | 1 |
| Roc-la-Tour | 2 | 4 46 59 | 49 53 26 | France | 1 |
| Roc-la-Tour | 3 | 4 46 59 | 49 53 26 | France | 1 |
| Roc-la-Tour | 4 | 4 46 59 | 49 53 26 | France | 1 |
| Beptenaz, G de |  | 5 15 02 | 45 43 27 | France | 2 |
| Tessonière, G. de la |  | 5 21 57 | 46 11 29 | France | 2 |
| Balme-Rousse | D2a | 5 23 37 | 45 04 00 | France | 2 |
| Gay, A. |  | 5 24 25 | 46 05 05 | France | 2 |
| Bobache, A. de | 4+3 | 5 24 55 | 44 58 02 | France | 2 |
| Gigny, G. de | IV sup. | 5 27 37 | 46 27 07 | France | 2 |
| Gigny, G. de | IV inf. | 5 27 37 | 46 27 07 | France | 2 |
| Chaze 2 |  | 5 32 24 | 46 45 52 | France | 2 |
| Hoteaux, Les |  | 5 35 06 | 45 50 16 | France | 2 |
| Bonne Femme |  | 5 37 11 | 45 38 55 | France | 2 |
| Olette, Gotte d l' |  | 5 37 28 | 45 11 27 | France | 2 |
| Fontabert |  | 5 37 29 | 45 19 02 | France | 2 |
| Rigny "En Terredey" | Coll. Daval | 5 38 14 | 47 28 18 | France | 2 |
| Pugieu, A. de |  | 5 38 54 | 45 49 14 | France | 2 |
| Chaumois-Boivin, G. de | 2 | 5 40 09 | 46 44 53 | France | 2 |
| Étrembières, G. du Four, l'Hôpital |  | 6 13 37 | 46 08 07 | France | 2 |
| Rochedane | D1/D2 | 6 45 31 | 47 22 24 | France | 2 |
| Sweikhuizen-Oude Stort |  | 5 50 36 | 50 57 15 | Netherlands | 2 |
| Schaelsberg, Valkenburg |  | 5 50 48 | 50 51 44 | Netherlands | 2 |
| Sweikhuizen-Koolweg |  | 5 51 01 | 50 57 01 | Netherlands | 2 |
| Echt |  | 5 52 01 | 51 06 27 | Netherlands | 2 |
| Mesch-Steenberg | total | 5 44 30 | 50 46 09 | Netherlands | 1 |
| Sweikhuizen-Groene Paal |  | 5 50 52 | 50 56 58 | Netherlands | 1 |
| Griendtsveen |  | 5 52 45 | 51 26 36 | Netherlands | 1 |
| Eyserheide | 160 | 5 55 55 | 50 50 18 | Netherlands | 1 |
| Trou de Blaireaux | III 1 | 4 44 36 | 50 06 54 | Belgium | 2 |
| Trou de Blaireaux | III 2 | 4 44 36 | 50 06 54 | Belgium | 2 |
| Trou de Blaireaux | III 3 | 4 44 36 | 50 06 54 | Belgium | 2 |
| Trou de Blaireaux | II 1 | 4 44 36 | 50 06 54 | Belgium | 2 |
| Trou du Frontal |  | 4 57 24 | 50 12 52 | Belgium | 2 |
| Roche Plate | 12 | 5 32 36 | 48 53 58 |  | 2 |
| Trou da Somme |  | 4 51 31 | 50 13 17 | Belgium | 1 |
| Bois Laiterie |  | 4 51 49 | 50 21 35 | Belgium | 1 |
| Roche-al-Rue |  | 4 52 47 | 50 13 13 | Belgium | 1 |
| Trou de Chaleux | 5 | 4 56 30 | 50 13 18 | Belgium | 1 |
| Trou de Chaleux | excavation Otte | 4 56 30 | 50 13 18 | Belgium | 1 |
| Trou des Nutons (Furfooz) |  | 4 57 11 | 50 12 44 | Belgium | 1 |
| Orp | est | 4 58 23 | 50 41 40 | Belgium | 1 |
| Orp | ouest | 4 58 23 | 50 41 40 | Belgium | 1 |
| Goyet 3 | chamber A, Horizont 1 | 5 00 48 | 50 26 44 | Belgium | 1 |
| Coléoptère, G. de | Niveau B | 5 31 07 | 50 22 43 | Belgium | 1 |
| Verlaine, G. de Sy |  | 5 31 08 | 50 24 22 | Belgium | 1 |
| Kanne | secteur central | 5 40 46 | 50 48 29 | Belgium | 1 |
| Trou Walou |  | 5 41 31 | 50 34 34 | Belgium | 1 |
| Bildstockfels, Engen, Baden |  | 8 46 14 | 47 52 07 | Germany | 2 |
| Petersfels | P6AH2 | 8 48 15 | 47 51 39 | Germany | 2 |
| Petersfels | P6AH3/4 | 8 48 15 | 47 51 39 | Germany | 2 |
| Probstfels | I-IV | 8 57 23 | 48 03 02 | Germany | 2 |
| Kuhstallhöhle |  | 9 06 59 | 48 10 45 | Germany | 2 |
| Burghöhle Dietfurt |  | 9 08 21 | 48 04 42 | Germany | 2 |
| Zigeunerfels | H/I | 9 09 35 | 48 05 14 | Germany | 2 |
| Annakapellenhöhe |  | 9 12 32 | 48 10 49 | Germany | 2 |
| Nikolaushöhle | 2 | 9 12 34 | 48 10 40 | Germany | 2 |
| Randecker Maar |  | 9 31 28 | 48 34 21 | Germany | 2 |
| Hohlefels Hütten | yellow | 9 38 18 | 48 22 23 | Germany | 2 |
| Aichbühl B |  | 9 40 03 | 48 01 43 | Germany | 2 |
| Schmiechenfels | 2 | 9 42 34 | 48 20 50 | Germany | 2 |
| Sirgenstein Südwand |  | 9 45 34 | 48 23 12 | Germany | 2 |
| Geißenklösterle | AH Io | 9 46 49 | 48 23 36 | Germany | 2 |
| Bocksteinhöhle | III | 10 09 17 | 48 31 59 | Germany | 2 |
| Bocksteinschmiede&Bocksteinloch | c | 10 09 21 | 48 31 59 | Germany | 2 |
| Bockstein-Törle | AH II, III | 10 09 24 | 48 31 59 | Germany | 2 |
| Hohlenstein-Stadel | II | 10 10 20 | 48 32 57 | Germany | 2 |
| Hohlenstein-Stadel | III | 10 10 20 | 48 32 57 | Germany | 2 |
| Kleine Scheuer im Hohlenstein-Stadel III | III | 10 10 21 | 48 32 57 | Germany | 2 |
| Vogelherd | III | 10 11 38 | 48 33 31 | Germany | 2 |
| Vogelherd | II | 10 11 38 | 48 33 31 | Germany | 2 |
| Weinberghöhlen | Vorplatz Höhle 1+2 | 11 02 10 | 48 45 30 | Germany | 2 |
| Weinberghöhlen | Saazer Loch | 11 02 10 | 48 45 30 | Germany | 2 |
| Weinberghöhlen | Graben außen | 11 02 10 | 48 45 30 | Germany | 2 |
| Weinberghöhlen | Vorplatz Höhle 3 | 11 02 10 | 48 45 30 | Germany | 2 |
| Eitensheim |  | 11 19 13 | 48 49 10 | Germany | 2 |
| Mittlere Klause | AH 1 | 11 47 04 | 48 56 04 | Germany | 2 |
| Sesselfelsgrotte | C2 | 11 47 22 | 48 56 06 | Germany | 2 |
| Sesselfelsgrotte | C1 | 11 47 22 | 48 56 06 | Germany | 2 |
| Sesselfelsgrotte | B3 | 11 47 22 | 48 56 06 | Germany | 2 |
| Sesselfelsgrotte | B2 | 11 47 22 | 48 56 06 | Germany | 2 |
| Siegfriedfelsen, A. am | D1 | 11 48 49 | 48 55 23 | Germany | 2 |
| Siegfriedfelsen, A. am | D2 | 11 48 49 | 48 55 23 | Germany | 2 |
| Siegfriedfelsen, A. am | D3 | 11 48 49 | 48 55 23 | Germany | 2 |
| Siegfriedfelsen, A. am | D4 | 11 48 49 | 48 55 23 | Germany | 2 |
| Obernederhöhle | 2 upper +1 | 11 50 15 | 48 55 60 | Germany | 2 |
| Tunnelhöhle |  | 11 58 44 | 49 01 54 | Germany | 2 |
| Fürst-Albrecht-Höhle |  | 11 58 54 | 48 59 21 | Germany | 2 |
| Viersener Donk I |  | 6 26 06 | 51 14 52 | Germany | 2 |
| Eierberg |  | 6 28 08 | 51 07 42 | Germany | 2 |
| Kamphausen |  | 6 28 22 | 51 07 35 | Germany | 2 |
| Irlich, Sandgrube |  | 7 26 16 | 50 27 02 | Germany | 2 |
| Feldhofhöhle | 2,3 4? | 7 51 17 | 51 22 40 | Germany | 2 |
| Balverhöhle |  | 7 52 19 | 51 20 21 | Germany | 2 |
| Dreieich-Götzenhein Nord (II) |  | 8 44 03 | 50 00 20 | Germany | 2 |
| Aschenstein |  | 9 54 03 | 51 55 09 | Germany | 2 |
| Quedlinburg, Bockshornschanze |  | 11 09 50 | 51 47 15 | Germany | 2 |
| Quedlinburg, Lehof |  | 11 09 54 | 51 48 41 | Germany | 2 |
| Gorsleben |  | 11 11 00 | 51 16 39 | Germany | 2 |
| Gatersleben |  | 11 17 13 | 51 49 24 | Germany | 2 |
| Königsaue, Bruchberg |  | 11 24 12 | 51 48 51 | Germany | 2 |
| Aschersleben | total | 11 27 40 | 51 45 11 | Germany | 2 |
| Rennerweg, Anhöhe |  | 11 30 45 | 50 47 09 | Germany | 2 |
| Forstbergplateau über dem Hain |  | 11 31 38 | 50 47 00 | Germany | 2 |
| Dehnamühle, üb. d. |  | 11 31 48 | 50 47 35 | Germany | 2 |
| Forstebrg |  | 11 31 59 | 50 47 19 | Germany | 2 |
| Herdloch bei Ranis | 1+2 | 11 33 51 | 50 39 43 | Germany | 2 |
| Kirchberg |  | 11 33 51 | 50 47 34 | Germany | 2 |
| Urdhöhle |  | 11 38 28 | 50 41 26 | Germany | 2 |
| Weißenborn, Speckberg |  | 11 52 55 | 50 55 27 | Germany | 2 |
| Petersberg |  | 11 57 00 | 51 35 36 | Germany | 2 |
| Weißenfels |  | 11 57 22 | 51 12 09 | Germany | 2 |
| Wallen (Friedensdorf / Kriegsdorf |  | 12 03 51 | 51 21 25 | Germany | 2 |
| Pfortener Berg, Gera |  | 12 05 11 | 50 50 49 | Germany | 2 |
| Schafgraben, Zwötzen |  | 12 05 30 | 50 50 48 | Germany | 2 |
| Kuppelberg |  | 12 44 10 | 51 19 26 | Germany | 2 |
| Wachtelberg A |  | 12 44 19 | 51 21 05 | Germany | 2 |
| Metten | I+II | 12 54 56 | 48 51 18 | Germany | 2 |
| Vilshofen-Kuffing |  | 13 10 50 | 48 35 56 | Germany | 2 |
| Niederlommatzsch |  | 13 24 25 | 51 14 22 | Germany | 2 |
| Baselitz | I | 13 29 01 | 51 14 02 | Germany | 2 |
| Baselitz | III | 13 29 01 | 51 14 02 | Germany | 2 |
| Baselitz | IV | 13 29 01 | 51 14 02 | Germany | 2 |
| Burk 2, Bautzen | 2 | 14 27 57 | 51 11 57 | Germany | 2 |
| Fußgönheim 2 |  | 8 17 06 | 49 27 01 | Germany | 2 |
| Fußgöhnheim 1 |  | 8 17 18 | 49 27 24 | Germany | 2 |
| Schweskau |  | 11 17 18 | 52 55 14 | Germany | 2 |
| Munzingen |  | 7 41 32 | 47 57 59 | Germany | 1 |
| Munzingen | GH4 1976-77 | 7 41 32 | 47 57 59 | Germany | 1 |
| Teufelsküche |  | 7 46 09 | 47 55 10 | Germany | 1 |
| Petersfels | P1AH2 | 8 48 15 | 47 51 39 | Germany | 1 |
| Petersfels | P1AH3A | 8 48 15 | 47 51 39 | Germany | 1 |
| Petersfels | P1AH3B | 8 48 15 | 47 51 39 | Germany | 1 |
| Petersfels | P1AH4 | 8 48 15 | 47 51 39 | Germany | 1 |
| Petersfels | P2 | 8 48 15 | 47 51 39 | Germany | 1 |
| Petersfels | P3AH1/2 | 8 48 15 | 47 51 39 | Germany | 1 |
| Petersfels | P3AH2 | 8 48 15 | 47 51 39 | Germany | 1 |
| Petersfels | P3AH2/3 | 8 48 15 | 47 51 39 | Germany | 1 |
| Petersfels | P3AH3 | 8 48 15 | 47 51 39 | Germany | 1 |
| Petersfels | P3AH3/4 | 8 48 15 | 47 51 39 | Germany | 1 |
| Petersfels | P3AH4 | 8 48 15 | 47 51 39 | Germany | 1 |
| Petersfels | P3AH5 | 8 48 15 | 47 51 39 | Germany | 1 |
| Petersfels | P5AH2 | 8 48 15 | 47 51 39 | Germany | 1 |
| Petersfels | P5AH4 | 8 48 15 | 47 51 39 | Germany | 1 |
| Petersfels | P5AH5 | 8 48 15 | 47 51 39 | Germany | 1 |
| Petersfels | P6AH4 | 8 48 15 | 47 51 39 | Germany | 1 |
| Petersfels | P6AH5 | 8 48 15 | 47 51 39 | Germany | 1 |
| Petersfels | P6AH6 | 8 48 15 | 47 51 39 | Germany | 1 |
| Petersfels | P3AH1 | 8 48 15 | 47 51 39 | Germany | 1 |
| Gnirshöhle 1 |  | 8 48 54 | 47 51 34 | Germany | 1 |
| Gnirshöhle 2 |  | 8 48 54 | 47 51 34 | Germany | 1 |
| Napoleonskopf |  | 8 54 01 | 48 27 27 | Germany | 1 |
| Käppele |  | 9 25 34 | 48 36 24 | Germany | 1 |
| Braunfirst |  | 9 31 52 | 48 35 28 | Germany | 1 |
| Felsställe | IIIb | 9 39 05 | 48 16 57 | Germany | 1 |
| Felsställe | IIIa | 9 39 05 | 48 16 57 | Germany | 1 |
| Schuntershöhle |  | 9 39 23 | 48 19 57 | Germany | 1 |
| Schussenquelle | main | 9 39 29 | 48 01 19 | Germany | 1 |
| Schussenquelle | Comp.A | 9 39 29 | 48 01 19 | Germany | 1 |
| Schussenquelle | Comp.C | 9 39 29 | 48 01 19 | Germany | 1 |
| Helga-Aabri | IIIa+b | 9 44 33 | 48 22 24 | Germany | 1 |
| Helga-Abri | IIId | 9 44 33 | 48 22 24 | Germany | 1 |
| Hohle Fels, Schelklingen | Ia | 9 45 20 | 48 22 20 | Germany | 1 |
| Hohle Fels, Schelklingen | Ib | 9 45 20 | 48 22 20 | Germany | 1 |
| Hohle Fels, Schelklingen | Ic | 9 45 20 | 48 22 20 | Germany | 1 |
| Hohle Fels, Schelklingen | IIa | 9 45 20 | 48 22 20 | Germany | 1 |
| Brillenhöhle | IV lower | 9 46 42 | 48 24 22 | Germany | 1 |
| Brillenhöhle | IV upper | 9 46 42 | 48 24 22 | Germany | 1 |
| Kleine Scheuer im Rosenstein | total | 9 56 39 | 48 47 19 | Germany | 1 |
| Spitzbubenhöhle | GH8+9=AH2 | 10 10 46 | 48 35 52 | Germany | 1 |
| Hohlenstein bei Ederheim |  | 10 27 55 | 48 48 32 | Germany | 1 |
| Kaufertsberg | yellow, lower | 10 36 39 | 48 48 33 | Germany | 1 |
| Kaufertsberg | yellow-brown, upper | 10 36 39 | 48 48 33 | Germany | 1 |
| Speckberg | A+B | 11 11 13 | 48 48 07 | Germany | 1 |
| Obere Klause | lower level | 11 46 38 | 48 56 13 | Germany | 1 |
| Obere Klause | upper level | 11 46 38 | 48 56 13 | Germany | 1 |
| Kastelhänghöhle | 5 | 11 47 11 | 48 55 45 | Germany | 1 |
| Barbing |  | 12 11 52 | 49 00 12 | Germany | 1 |
| Beeck |  | 6 11 30 | 50 58 52 | Germany | 1 |
| Alsdorf |  | 6 11 42 | 50 51 10 | Germany | 1 |
| Kartstein | 5 | 6 39 34 | 50 32 40 | Germany | 1 |
| Andernach-Martinsberg | KI | 7 24 01 | 50 26 01 | Germany | 1 |
| Andernach-Martinsberg | KII | 7 24 01 | 50 26 01 | Germany | 1 |
| Andernach-Martinsberg | KIII | 7 24 01 | 50 26 01 | Germany | 1 |
| Andernach-Martinsberg | K IV | 7 24 01 | 50 26 01 | Germany | 1 |
| Gönnersdorf | I | 7 24 45 | 50 26 46 | Germany | 1 |
| Gönnersdorf | II | 7 24 45 | 50 26 46 | Germany | 1 |
| Gönnersdorf | III | 7 24 45 | 50 26 46 | Germany | 1 |
| Gönnersdorf | IV | 7 24 45 | 50 26 46 | Germany | 1 |
| Martinshöhle, Letmathe |  | 7 36 37 | 51 22 03 | Germany | 1 |
| Wildweiberlei |  | 7 59 27 | 50 22 10 | Germany | 1 |
| Wildscheuer | V | 8 07 47 | 50 25 12 | Germany | 1 |
| Dreieich-Götzenhain Ost (I) | total | 8 44 23 | 49 59 55 | Germany | 1 |
| Gadenstedt |  | 10 12 54 | 52 14 48 | Germany | 1 |
| Bad Frankenhausen | total | 11 04 26 | 51 22 12 | Germany | 1 |
| Quedlinburg, Mühlgraben |  | 11 11 26 | 51 49 20 | Germany | 1 |
| Teufelsbrücke | 1 | 11 23 28 | 50 37 15 | Germany | 1 |
| Teufelsbrücke | 2 | 11 23 28 | 50 37 15 | Germany | 1 |
| Teufelsbrücke | 3 | 11 23 28 | 50 37 15 | Germany | 1 |
| Teufelsbrücke | 4 | 11 23 28 | 50 37 15 | Germany | 1 |
| Nebra |  | 11 34 16 | 51 17 32 | Germany | 1 |
| Jena "an der hohen Saale" |  | 11 35 22 | 50 55 19 | Germany | 1 |
| Rothestein "Kuhberg" |  | 11 35 45 | 50 50 41 | Germany | 1 |
| Kahla-Löbschütz |  | 11 35 56 | 50 48 24 | Germany | 1 |
| Ölknitz |  | 11 36 11 | 50 50 41 | Germany | 1 |
| KnieG. |  | 11 38 29 | 50 41 29 | Germany | 1 |
| Lausnitz, A. Theure |  | 11 41 24 | 50 43 20 | Germany | 1 |
| Saaleck |  | 11 42 12 | 51 06 45 | Germany | 1 |
| Lengefeld | surface | 11 42 19 | 51 06 49 | Germany | 1 |
| Galgenberg, Halle |  | 11 58 33 | 51 30 09 | Germany | 1 |
| Gleina |  | 11 59 06 | 50 56 04 | Germany | 1 |
| Ahlendorf |  | 11 59 10 | 50 59 07 | Germany | 1 |
| Gera-Liebschwitz, Binsenacker |  | 12 05 19 | 50 49 35 | Germany | 1 |
| Groitzsch A | A1 | 12 17 17 | 51 09 40 | Germany | 1 |
| Groitzsch A | A2 | 12 17 17 | 51 09 40 | Germany | 1 |
| Groitzsch B | B | 12 17 20 | 51 09 41 | Germany | 1 |
| Groitzsch B | B-Nord | 12 17 20 | 51 09 41 | Germany | 1 |
| Groitzsch C | C1 | 12 17 24 | 51 09 43 | Germany | 1 |
| Groitzsch C | C2 | 12 17 24 | 51 09 43 | Germany | 1 |
| Groitzsch C | C3+D-Nord | 12 17 24 | 51 09 43 | Germany | 1 |
| Groitzsch D | D2 | 12 17 24 | 51 09 52 | Germany | 1 |
| Groitzsch D | D1 | 12 17 24 | 51 09 52 | Germany | 1 |
| Steinberg |  | 7 46 28 | 47 55 38 | Germany | 2 |
| Neuchâtel-Monruz | 2M/A | 6 57 32 | 47 00 13 | Switzerland | 1 |
| Neuchâtel-Monruz | 1M | 6 57 32 | 47 00 13 | Switzerland | 1 |
| Hauterive-Champréveyres | 1 inf. | 6 58 16 | 47 00 24 | Switzerland | 1 |
| Hauterive-Champréveyres | 2 | 6 58 16 | 47 00 24 | Switzerland | 1 |
| Hauterive-Champréveyres | 1princ. | 6 58 16 | 47 00 24 | Switzerland | 1 |
| Hauterive-Champréveyres | 3 | 6 58 16 | 47 00 24 | Switzerland | 1 |
| Alle, Noir Bois |  | 7 07 09 | 47 25 04 | Switzerland | 1 |
| Liesberg |  | 7 26 11 | 47 23 37 | Switzerland | 1 |
| Moosseedorf-Moosbühl |  | 7 29 25 | 47 00 49 | Switzerland | 1 |
| Brügglihöhle |  | 7 33 42 | 47 26 50 | Switzerland | 1 |
| Kastelhöhle-Nord | middle | 7 34 09 | 47 25 46 | Switzerland | 1 |
| Kastelhöhle-Nord | upper | 7 34 09 | 47 25 46 | Switzerland | 1 |
| Heidenküche |  | 7 34 10 | 47 26 05 | Switzerland | 1 |
| Bruderholz |  | 7 36 20 | 47 31 49 | Switzerland | 1 |
| Birseck-Ermitage |  | 7 37 45 | 47 29 27 | Switzerland | 1 |
| Hollenberg-Höhle 3 | D | 7 37 49 | 47 29 21 | Switzerland | 1 |
| Bolken, Inkwilersee |  | 7 39 47 | 47 11 28 | Switzerland | 1 |
| Rheinfelden-Eremitage | 1 | 7 48 01 | 47 32 40 | Switzerland | 1 |
| Sälihöhle-Oben |  | 7 54 48 | 47 20 21 | Switzerland | 1 |
| Hard I |  | 7 54 51 | 47 21 16 | Switzerland | 1 |
| Hard II |  | 7 54 56 | 47 21 21 | Switzerland | 1 |
| Wilmatt |  | 7 55 02 | 47 22 26 | Switzerland | 1 |
| Käsloch |  | 7 55 25 | 47 22 01 | Switzerland | 1 |
| Mühleloch, A. |  | 7 55 30 | 47 20 26 | Switzerland | 1 |
| Reiden-Stumpen |  | 7 58 19 | 47 15 27 | Switzerland | 1 |
| Einsiedeln-Langrüti | total | 8 47 21 | 47 09 07 | Switzerland | 1 |
| Schweizersbild | total | 8 38 15 | 47 43 29 | Switzerland | 1 |
| Freudenthal-Höhle, Rosenhalde |  | 8 38 19 | 47 40 56 | Switzerland | 1 |
| Kesslerloch-Thayngen |  | 8 41 44 | 47 44 46 | Switzerland | 1 |
| Scé du Châterlard, G. |  | 6 56 04 | 46 23 50 | Switzerland | 2 |
| Neuchâtel-Monruz | 1A | 6 57 32 | 47 00 13 | Switzerland | 2 |
| Hauterive-Champréveyres | 1A | 6 58 16 | 47 00 24 | Switzerland | 2 |
| Büttenloch | A | 7 32 20 | 47 28 36 | Switzerland | 2 |
| Büttenloch | B | 7 32 20 | 47 28 36 | Switzerland | 2 |
| Chesselgraben |  | 7 32 30 | 47 22 22 | Switzerland | 2 |
| Kohlerhöhle |  | 7 34 04 | 47 25 58 | Switzerland | 2 |
| Fürsteiner, Grabung 43/44 |  | 7 39 53 | 47 09 17 | Switzerland | 2 |
| Burgäschi Hintere Burg |  | 7 40 17 | 47 10 23 | Switzerland | 2 |
| Rislisberghöhle, Oensingen |  | 7 41 53 | 47 17 45 | Switzerland | 2 |
| Trimbach |  | 7 53 34 | 47 21 37 | Switzerland | 2 |
| Winznau-Köpfli (Altgr. U. Lesefunde) |  | 7 55 16 | 47 22 10 | Switzerland | 2 |
| Sandmatt, 25 |  | 8 01 57 | 47 10 49 |  | 2 |
| Moos, 14 |  | 8 02 37 | 47 10 01 | Switzerland | 2 |
| Seewagermoos, 17 |  | 8 02 48 | 47 09 56 |  | 2 |
| Seewagen, 24 (Bolt VIa) |  | 8 03 26 | 47 10 14 | Switzerland | 2 |
| Solgen Im Grauen |  | 8 33 51 | 47 36 43 | Switzerland | 2 |
| Gudenushöhle |  | 15 35 09 | 48 25 00 | Austria | 1 |
| Kamegg |  | 15 39 27 | 48 36 41 | Austria | 1 |
| Pekárna | i | 16 44 33 | 49 14 35 | Czech Republic | 1 |
| Dobříčany |  | 13 36 01 | 50 18 40 | Czech Republic | 1 |
| Tmaň, Děravá cave | lower l. | 14 02 09 | 49 54 00 | Czech Republic | 1 |
| Tmaň, Děravá cave | upper l. | 14 02 09 | 49 54 00 | Czech Republic | 1 |
| Kvíc |  | 14 03 50 | 50 12 42 | Czech Republic | 1 |
| Putim | 773 | 14 05 55 | 49 15 40 | Czech Republic | 1 |
| Putim | 162, 767 | 14 05 55 | 49 15 40 | Czech Republic | 1 |
| Keblice |  | 14 06 08 | 50 28 49 | Czech Republic | 1 |
| Hostim | total | 14 07 06 | 49 47 16 | Czech Republic | 1 |
| Malomĕřice-BorkyI |  | 16 38 33 | 49 12 54 | Czech Republic | 1 |
| Býči skála |  | 16 41 27 | 49 18 32 | Czech Republic | 1 |
| Žitného |  | 16 44 03 | 49 17 28 | Czech Republic | 1 |
| Kolíbky | 1 | 16 44 12 | 49 19 59 | Czech Republic | 1 |
| Kolíbky | 5 | 16 44 12 | 49 19 59 | Czech Republic | 1 |
| Kolíbky | 6 | 16 44 12 | 49 19 59 | Czech Republic | 1 |
| Kůlna-Höhle | 5 | 16 44 16 | 49 24 33 | Czech Republic | 1 |
| Kůlna-Höhle | 6 | 16 44 16 | 49 24 33 | Czech Republic | 1 |
| Hadí |  | 16 44 31 | 49 14 24 | Czech Republic | 1 |
| Pekárna | g/h | 16 44 33 | 49 14 35 | Czech Republic | 1 |
| Adlerova |  | 16 44 54 | 49 14 26 | Czech Republic | 1 |
| Křižova |  | 16 44 55 | 49 14 35 | Czech Republic | 1 |
| Ochozska-Höhle |  | 16 45 03 | 49 14 38 | Czech Republic | 1 |
| Mokrá, Lom V |  | 16 45 16 | 49 14 05 | Czech Republic | 1 |
| Balcarova |  | 16 45 30 | 49 22 35 | Czech Republic | 1 |
| Loštice I |  | 16 55 15 | 49 43 41 | Czech Republic | 1 |
| Hranice | III | 17 45 30 | 49 32 36 | Czech Republic | 1 |
| Barrandová cave |  | 14 08 18 | 49 56 35 | Czech Republic | 2 |
| Želeč |  | 13 33 27 | 50 14 17 | Czech Republic | 2 |
| Bečov |  | 13 43 03 | 50 26 54 | Czech Republic | 2 |
| Dolni Poříčí 8 |  | 13 47 32 | 49 17 09 | Czech Republic | 2 |
| Slaník I |  | 13 57 08 | 49 16 05 | Czech Republic | 2 |
| Koněprusy |  | 14 02 50 | 49 54 59 | Czech Republic | 2 |
| Lhota |  | 14 04 56 | 49 15 38 | Czech Republic | 2 |
| Krápníková |  | 14 07 04 | 49 57 15 | Czech Republic | 2 |
| Na Průchodě |  | 14 07 41 | 49 58 16 | Czech Republic | 2 |
| Borečnice 2 |  | 14 08 21 | 49 21 40 | Czech Republic | 2 |
| Rytiřská |  | 14 12 46 | 50 46 21 | Czech Republic | 2 |
| Žd'ár 1 |  | 14 13 39 | 49 13 58 | Czech Republic | 2 |
| Březnice |  | 14 30 53 | 49 15 05 | Czech Republic | 2 |
| Náchod |  | 16 09 57 | 50 25 17 | Czech Republic | 2 |
| Barová-Höhle | 11-12 | 16 41 37 | 49 18 41 | Czech Republic | 2 |
| Líšň-Čtvrtĕ |  | 16 41 41 | 49 12 25 | Czech Republic | 2 |
| Nová Drátenická Cave | 15 | 16 43 55 | 49 17 29 | Czech Republic | 2 |
| Verunčina |  | 16 44 08 | 49 22 11 | Czech Republic | 2 |
| Kulnička |  | 16 44 26 | 49 14 35 | Czech Republic | 2 |
| Švédův stůl | 50 | 16 44 55 | 49 14 41 | Czech Republic | 2 |
| Mokrá, Lom I |  | 16 45 03 | 49 14 03 | Czech Republic | 2 |
| Šipka dira |  | 18 06 59 | 49 35 14 | Czech Republic | 2 |
| Węgliny |  | 14 43 08 | 51 49 24 | Poland | 1 |
| Broniszowice 2 |  | 17 10 39 | 50 25 37 | Poland | 1 |
| Dzierzyslaw 35 | excavation 1999-2003 | 17 59 19 | 50 03 35 | Poland | 1 |
| Dra Majera |  | 19 01 38 | 50 09 09 | Poland | 1 |
| Brzoskwinia-Krzemionki7 | 1 | 19 43 00 | 50 04 00 | Poland | 1 |
| Brzoskwinia-Krzemionki7 | 2 | 19 43 00 | 50 04 00 | Poland | 1 |
| Maszycka Cave |  | 19 49 60 | 50 13 00 | Poland | 1 |
| Katy 1 |  | 20 21 01 | 49 25 00 | Poland | 1 |
| Maly Antoniów |  | 21 31 49 | 51 03 06 | Poland | 1 |
| Wilczyce | 10 | 21 39 24 | 50 44 34 | Poland | 1 |
| Klementowice-Kolonia 20 |  | 22 08 23 | 51 20 56 | Poland | 1 |
| Hłomcza |  | 22 16 50 | 49 38 01 | Poland | 1 |
| Grodzisko Dolne, Leżajsk |  | 22 27 45 | 50 09 45 | Poland | 1 |
| Przemysl 3 |  | 22 46 04 | 49 47 01 | Poland | 1 |
| Deszcrowa | 3 | 19 31 48 | 50 34 38 | Poland | 2 |
| Cyprzanów 3 |  | 18 06 55 | 50 04 19 | Poland | 2 |
| Bierawa |  | 18 14 29 | 50 16 51 | Poland | 2 |
| Krusza Skala |  | 19 34 14 | 50 33 49 | Poland | 2 |
| Zalas | 7 to 9 | 19 37 41 | 50 04 58 | Poland | 2 |
| Zalas | 10 to 11 | 19 37 41 | 50 04 58 | Poland | 2 |
| Zawalona | E | 19 43 36 | 50 03 36 | Poland | 2 |
| Puchaczowa Skala |  | 19 50 00 | 50 11 01 | Poland | 2 |
| Rydno, II/59 |  | 20 57 42 | 51 07 58 | Poland | 2 |
| Seredzice |  | 21 10 25 | 51 09 18 | Poland | 2 |

**References**

Altuna, J. (2003). Cueva de Aitzbitarte III (Errenteria), Arkeiokuska. *Investigación Arqueológica,* *2002*, 116-118.

Altuna, J., Mariezkurrena, K., De la Peña, P., & Ríos-Garaizar, J. (2013). Los niveles gravetienses de la cueva de Aitzbitarte III (Gipuzkoa). Industrias y faunas asociadas. In : C. De las Heras, J.A. Lasheras, Á. Arrizabalaga, & M. De la Rasilla (Eds.), *Pensando el Gravetiense : nuevos datos para la región cantábria en su contexto peninsular y pirenaico* (pp. 184-204). Madrid : Monografías del Museo Nacional y Centro de Investigación de Altamira, n.° 23.

Arrizabalaga, Á. (2000). El yacimiento arqueológicos de Labeko Koba (Arrasate, País Vasco). Entorno. Crónica de las investigaciones. Estratigrafía y estructuras. Cronología absoluta. In: Á. Arrizabalaga, & J. Altuna (Eds.), *Labeko Koba (País Vasco). Hienas y humanos en los albores del Paleolítico superior* (pp. 15-72). San Sebastián: Munibe, vol. 52. Sociedad de Ciencias Naturals Aranzadi.

Arrizabalaga, À. (2009). The Middle to Upper Paleolithic transition on the Basque Crossroads: main sites, key issues. *Mitteilungen der Gesellschaft für Urgeschichte,* *18*, 39-70.

Arsuaga, J.L., Villaverde Bonilla, V., Quam, R., Gracia, A., Lorenzo, C., Martínez, I., & Carretero, J.M. (2002). The Gravettian occipital bone from the site of Malladetes (Barx, Valencia, Spain). *Journal of Human Evolution,* *43*(3), 381-393.

Aubry, T., Fontugne, M., &. Moura, M.-H. (1997). Les occupations de la grotte de Buraca Grande depuis le Paléolithique supérieur et les apports de la séquence holocène à l’étude de la transition mésolithique/néolithique au Portugal. *Bulletin de la Société Préhistorique Française,* *94*(2), 182-190.

Aubry, T., Brugal, J.-P., Chavière, F.-X., Figueral, I., Moura, M.-H., & Plisson, H. (2001). Modalités d’occupations au Paléolithique supérieur dans la grotte de Buraca Escura (Redinha, Pombal, Portugal). *Revista Portugesa de Arqueologia,* *4*, 19-46.

Aura Tortosa, J.E., Jorda, F., González-Tablas, J., & Sanchidrián, J.L. (1998). Secuencia arqueológica de la Cueva de Nerja: La sala del Vestíbulo. In: J.L. Sanchidrián, & M.D. Simón Vallejo (Eds.), *Las culturas del Pleistoceno superior en Andalucía. Patronato de la Cueva de Nerja* (pp. 217-236). Málaga.

Aura Tortosa, J.E., Jordá Pardo, J.F., & Fortea Pérez, J., (2006). La Cueva de Nerja (Málaga, España) y los inicios del Solutrense en Andalucía. *Zephyrus,* *59*, 69-88.

Baldeón, A., & Berganza, E. (1997). *El yacimiento epipaleolítico de Kukuma. Un asentamiento de cazadores-recolectores en la Llanada alavesa (Araia, Álava)*. Memorias de yacimientos alaveses. Vitoria: Diputación Foral de Álava.

Barandiarán, I., Cava, A., & Alday, A. (2006). Ocupaciones de altura e interior durante el Tardiglaciar: la Llanada alavesa y sus estribaciones montañosas. Miscelánea en homenaje a Victoria Cabrera. *Zona Arqueológica,* *7*, 534-551.

Benazzi, S., Douka, K., Fornai, C., Bauer, C.C., Kullmer, O., Svoboda, J., Pap, I., Mallegni, F., Bayle, P., Coquerelle, M., Condemi, S., Ronchitelli, A., Harvati, K., & Weber, G.W. (2011). Early dispersal of modern humans in Europe and implications for Neanderthal behaviour. *Nature,* *479*, 525-529.

Bergadá, M.M. (1998). *Estudio geoarqueológico de los asentamientos prehistóricos del Pleistoceno Superior y el Holoceno inicial en Catalunya*. BAR International Series 742. Oxford: Oxford University Press.

Bernaldo de Quirós, F., Castaños, P., Maíllo-Fernández, J.M., & Neira, A. (2013). El Gravetiense de la cueva de El Castillo. Nuevos datos. In: C. De las Heras, J.A. Lasheras, Á. Arrizabalaga, & M. De la Rasilla (Eds.), *Pensando el Gravetiense : nuevos datos para la región cantábria en su contexto peninsular y pirenaico* (pp. 264-275). Madrid: Monografías del Museo Nacional y Centro de Investigación de Altamira, n.° 23.

Blasco, M.F., & Rodanés, J.M. (2004). La Cueva del Gato-2 (Épila, Zaragoza): la dinámica de ocupación desde el 18000 a.C. hasta nuestros días. *Naturaleza Aragonesa,* *12*, 66-75.

Cabrera Valdés, V., Maíllo-Fernández, J.M., Lloret, M., & Bernaldo de Quirós, F. (2001). La transition vers le Paléolithique supérieure dans la grotte du Castillo (Cantabrie, Espagne): la couche 18. *L’Anthropologie,* *105*(4), 505-532.

Charles, R., Hedges, R., & Jadin, I. (2003). Aurignacian point, butchery remains and Radiocarbon Accelerator Dates from the Trou Magrite at Pont-à-Lesse (Commune of Dinat, Province of Namur, Belgium). *Anthropologica et Praehistorica,* *114*, 81-84.

Combier, J., & Jouve, G. (2012). Chauvet cave’s art is not Aurignacian: a new examination of the archaeological evidence and dating procedures. *Quartär*, *59*, 131-152.

Conard, N.J. (2009). A female figurine from the basal Aurignacian of Hohle Fels Cave in southwestern Germany. *Nature,* *459*, 248-252.

Conard, N.J., & Bolus, M. (2008). Radiocarbon dating the late Middle Paleolithic and Aurignacian of the Swabian Jura. *Journal of Human Evolution,* *55*(5), 886-897.

Cortés Sánchez, M. (2007). *Cueva Bajondillo (Torremolinos). Secuencia cronocultural y paleoambiental del Cuaternario reciente en la Bahía de Málaga*. Málaga: Centro de ediciones de la diputación de Málaga.

Dinnis, R. (2012). The timing of Aurignacian occupation of the British Peninsula. *Quartär,* *59*, 67-83.

Douka, K., Perlès, C., Valladas, H., Vanhaeren, M., & Hedges, R.E.M. (2011). Franchthi cave revisited: the age of the Aurignacian in south-eastern Europe. *Antiquity, 85*(330), 1131-1150.

Douka, K., Grimaldi, S., Boschian, G., del Lucchese, A., & Higham, T.F.C. (2012). A new chronostratigraphic framework for the Upper Paleolithic of Riparo Mochi (Italy). *Journal of Human Evolution,* *62*(2), 286-299.

Duarte, C., Maurício, J., Pettitt, P., Souto, P., Trinkaus, E., Van der Pflicht, H., & Zilhão, J. (1999). The early Upper Paleolithic human skeleton from the Abrigo do Lagar Velho (Portugal) and modern human emergence in Iberia. *Proceedings of the National Academy of Science,* *96*(13), 7604-7609.

Finlayson, C., Pacheco, F.G., Rodríguez-Vidal, J., Fa, D.A., López, J.M.G., Pérez, A.S., Finlayson, G., Allue, E., Preysler, J.B., Cáceres, I., Carríon, J.S., Jalvo, Y.F., Gleed-Owen, C.P., Espejo, F.J.J., López, P., Sáez, J.A.L., Cantal, J.A.R, Marco, A.S., Guzman, F.G., Brown, K., Fuentes, N., Valarino, C. A., Villapando, A., Stringer, C.B., Ruiz, F.M., & Sakamoto, T. (2006). Late survival of Neanderthals at the southernmost extreme of Europe. *Nature, 443*, 850-853.

Hedges, R.E.M., Pettitt, P., Bronk Ramsey, C., & Van Klinken, G.J. (1997). Radiocarbon dates from the Oxford AMS system: Archaeometry datelist 24. *Archaeometry,* *39*(2), 445-471.

Higham, T., Brock, F., Peresani, M., Broglio, A., Wood, R., & Douka, K. (2009). Problems with radiocarbon dating the Middle to Upper Palaeolithic transition in Italy. *Quaternary Science Reviews,* *28*(13-14), 1257-1267.

Higham, T., Compton, T., Stringer, C., Jacobi, R., Shapiro, B., Trinkaus, E., Chandler, B., Gröning, F., Collins, C., Hillson, S., O’Higgins, P., FitzGerald, C., & Fagan, M. (2011). The earliest evidence for anatomically modern humans in northwestern Europe. *Nature, 479*, 521-524.

Higham, T., Basell, L., Jacobi, R., Wood, R., Bronk Ramsey, C., & Conard, N.J. (2012). Testing models for the beginning of the Aurignacian and the advent of figurative art and music: The radiocarbon chronology of Geißenklösterle. *Journal of Human Evolution,* *62*(6), 664-676.

Iturbe, G., & Cortell, E. (1987). Las dataciones de Cova Beneito y su interés para el Paleolítico mediterráneo. *Trabajos de Prehistoria,* *44*, 267-270.

Iturbe, G., & Cortell, E. (1992). El Musteriense Final Mediterráneo: nuevas aportaciones. In: P. Utrilla (Ed.), *Aragón/Litoral Mediterráneo. Intercambios culturales durante la Prehistoria* (pp. 117-127). Zaragoza: Institución Fernando el Católico.

Jacobi, R., & Higham, T. (2008). The “Red Lady” ages gracefully: new ultrafiltration AMS determinations from Paviland. *Journal of Human Evolution,* *55*(5), 898-907.

Jöris, O., Street, M., Terberger, T., & Weninger, B. (2011). Radiocarbon Dating the Middle to Upper Paleolithic Transition: the Demise of the Last Neanderthals and the First Appearance of Anatomically Modern Humans in Europe. In: S. Condemi, & G.-C. Weniger (Eds.), *Continuity and Discontinuity in the Peopling of Europe. One Hundred and Fifty Years of Neanderthal Study* (pp. 239-298). Vertebrate Paleobiology and Paleoanthropology. Dordrecht: Springer.

Mangado, X., Petit, M.A., Fullola, J.M., & Bartrolí, R. (2006). El Paleolític superior de la Cova del Parco (Alós de Balaguer, La Noguera). *Revista d’Arqueologia de Ponent,* *16-17*, 45-62.

Mangado, X., Mercadal, O., Fullola, J.M., & Grimao, J. (2009). *Montlleó: un punt clau en la travessa del Pirineu. Els Pirineus i les àrees circumdants durant el Tardiglacial* (pp. 549-564). Puigcerdá: XIV Col.loqui Internacional d’Arqueologia de Puigcerdà (Novembre 2006).

Martínez-Moreno, J., Mora Torcal, R., & De la Torre, I. (2010). The Middle to Upper Palaeolithic transition in Cova Gran (Catalunya, Spain) and the extinction of Neanderthals in the Iberian Peninsula. *Journal of Human Evolution,* *58*, 211-226.

Maroto, J., Soler, N., & Fullola, J.M. (1996). Cultural change between Middle and Upper Paleolithic in Catalonia. In: E. Carbonell, & M. Vaquero (Eds.), *The Last Neandertals, the First Anatomically Modern Humans: A Tale about the Human Diversity. Cultural Change and Human Revolution at 40 Ka BP* (pp. 210-250). Universitat Rovira i Virgili, Capellades.

Maroto, J., Vqauero, M., Arriazabalaga, A., Baena, J., Baquedano, E., Jordá, J.F., Juliá, R., Montes, R., Van Der Pflicht, J., Rasines, P., & Wood, R. (2012). Current issues in late Middle Palaeolithic chronology: new assessments from Northern Iberia. *Quaternary International,* *247*, 15-25.

Montes, L. (2005). *El magdaleniense en el Prepirineo aragonés: últimos hallazgos* (pp. 183-192). Actas do IV Congresso de Arqueologia Peninsular. O Paleolítico. Faro: Promontoria Monograﬁca 2.

Mora, R., Benito-Calvo, A., Martínez-Moreno, J., González Marcén, P., & De la Torre, I. (2011). Chrono-stratigraphy of the Upper Pleistocene and Holocene sequence in Cova Gran (South-eastern Pre-Pyrenees, Iberian Peninsula). *Journal of Quaternary Science,* *26*(6), 635-644.

Nigst, P. R., & Haesaerts, P. (2012). L’Aurignacien en Basse Autriche : résultats préliminaires de l’analyse technologique de la couche culturelle 3 de Willendorf II et ses implications pour la chronologie du Paléolithique supérieur ancien en Europe centrale. *L’Anthropologie,* *116*(4), 575-608.

Nuin, J. (1996). Investigaciones en el yacimiento paleolítico superior de Legintxiki (Etxauri, Navarra). *Trabajos de Arqueología Navarra,* *12*, 280-282.

Peña Alonso, P. (2009). Revisión crítica de los conjunctos líticos gravetienses y su context arqueológico en la Península Ibérica. *Complutum,* *20*, 29-53.

Pettitt, P., Bronk Ramsey, C., Hedges, R.E.M., & Hodgins, G.W.L. (2000). AMS radiocarbon dating at Oxford and its contribution to issues of the extinction of Neanderthals and the spread of Homo sapiens sapiens across Eurasia. *Nuclear Instruments and Methods in Physics Research B, 172*(1-4). 751-755.

Pinto Llona, A.C., Clark, G.A., & Miller, A. (2006). Resultados preliminaries de los trabajos en curso en el abrigo de Sopeña (Onís, Asturias). In : V. Cabrera Valdés, F. Bernaldo de Quirós, & J.M. Maíllo-Fernández (Eds.), *En el centenario de la cueva de El Castillo : el ocaso de los neandertales* (pp. 193-208). Madrid: UNED (Universidad Nacional de Ecudación a Distancia).

Rasines del Río, P. (2005). El final de la transición. Dataciones de las primeras ocupaciones del Paleolítico superior en el centro de la Región Cantábrica. In: J.A. Lasheras Corruchaga, & R. Montes Barquín (Eds.), *Neandertales cantábricos, estado de la cuestión* (pp. 577-587). Santander: Monografías 20 (Museo Nacional y Centro de Investigacíon de Altamira).

Rasines del Río, P. (2009). Geografia, estratigrafia y cronología de la cueva de Cobrante. In: P. Rasines del Río (Ed.), *Arqueología en la cueva de Cobrante (Cantabria, España)*(pp. 35-243). Sautuola: Revista del Instituto de Prehistoria y Arqueologia Sautuola, vol. 15.

Sinitsyn, A. (2003). The most ancient sites of Kostenki in the context of the Initial Upper Palaeolithic from northern Eurasia. In: J. Zilhão, & F. D’Errico (Eds.), *The chronology of the Aurignacian and of the transitional technocomplexes: dating, stratigraphies, cultural implications* (pp. 89-107). Lisboa: Trabalhos de Arqueologia vol. 33.

Soler Subils, J., Soler Masferrer, N., & Maroto, J. (2008). L’Arbreda’s archaic Aurignacian dates clarified. *Eurasian Prehistory, 5*(2), 45-55.

Soto-Barreiro, M.J. (2003). *Cronología radiométrica, ecología y clima del Paleolitico cantábrico*. Madrid: Monografias 19 (Museo Nacional y Centro de Investigacíon de Altamira).

Straus, L. G. (1995). Archaeological description of the sequence. In: M. Otte, & L.G. Straus (Eds.), *Le Trou Magrite fouilles 1991-1992: Résurrection d’un site classique en Wallonie* (pp. 55-86). ERAUL 69. Liège: Liège University Press.

Straus, L.G., Altuna, J., Carvalho, E., Jackes, M., & Kunst, M. (1988). New excavations in Casa da Moura (Serra d’el Rei, Peniche) and at the Abrigos da Bocas (Rio Maior). *Arqueologia,* *18*, 65-95.

# Tsanova, T., Zwyns, N., Eizenberg, L., Teyssandier, N., Le Brun-Ricalens, & Otte, M. (2012). Le plus petit dénominateur commun : réflexion sur la variabilité des ensembles lamellaires du Paléolithique supérieur ancien d’Eurasie. Un bilan autour des exemples de Kozarnika (Est des Balkans) et Yafteh (Zagros central). *L’Anthropologie,* *116*(4), 469-509.

Uthmeier, Th. (2004). *Micoquien, Aurignacien und Gravettien in Bayern. Eine regionale Studie zum Übergang vom Mittel- zum Jungpaläolithikum*. Archäologische Berichte 18. Bonn: Dr. Rudolf Habelt.

Utrilla, P., & Mazo, C. (2007). La Peña de Las Forcas de Graus (Huesca). Un asentamiento reiterado desde el Magdaleniense Inferior al Neolítico Antiguo. *Saldvie,* *7*, 9-37.

Utrilla, P., González-Sampériz, P., Ferrer, C., & Blasco, M.F. (1999). La ocupación magdaleniense del río Henar: los asentamientos de Cetina (Zaragoza) y Deza (Soria). In: *Geología i Quaternari Litoral* (pp. 283-296). Memorial M.P. Fumanal. Valencia: Universidad de Valencia.

Utrilla, P., Domingo, R., Montes, L., Mazo, C., Rodanés, J.M., Blasco, F., & Alday, A. (2012). The Ebro Basin in NE Spain: A crossroads during the Magdalenian. *Quaternary International, 272-273*, 88-104.

Uzquiano Ollero, P., Arbizu Senosiain, M. Arsuaga Ferreras, J.L., Adán Álvarez, G.E., Aranburu, A., & Iriarte, E. (2008). Datos paleoflorísticos en la cuenca media del Nalón entre 40-32 ka BP: Antracoanálisis de la Cueva del Conde (Santo Adriano, Asturias). *Cuaternario y* *Geomorfología,* *22*, 121-133.

Vaquero, M. (1997). *Tecnologica lítiqua y comportamiento humano: organización de las actividades y cambio diacronico en el paleolitico medio del Abric Romaní*. PhD Dissertation, Universitat Rovira i Virgili Tarragona.

Vega, L.G., & Martin, P. (2006). Análisis preliminary de las cadenzas operativas en le material lítico procedente del nivel IV del Abrigo del Palomar (Yeste, Albacete). In: J.M. Maíllo-Fernández, & E. Baquedano (Eds.), *Miscelánea en Homenaje a Victoria Cabrera* (pp. 396-405). Madrid: Zona Arqueológica, vol. 7.

Villaverde Bonilla, V., & Roman, D. (2004). Avance al estudio de los niveles gravetienses de la Cova de les Cendres. *Archivo de Prehistoria Levantina, 25*, 19-59.

Wood, R.E., Arrizabalaga, A., Camps, M., Fallon, S., Iriarte-Chiapusso, M.-J., Jones, R., Maroto, J., De la Rasilla, M., Santamaría, D., Soler, J., Soler, N., Villaluenga, A., & Higham, T.F.G. (2014). The chronology of the earliest Upper Palaeolithic in northern Iberia: New insights from L’Arbreda, Labeko Koba and La Viña. *Journal of Human Evolution,* *69*, 91-109.

Zilhão, J. (2006). Chronostratigraphy of the Middle-to-Upper Paleolithic transition in the Iberian Peninsula. *Pyrenae,* *37*(1), 7-84.

Zilhão, J., & Trinkaus, E. (2000). *Portrait of the Artist as a Child. The Gravettian Human Skeleton from the Abrigo do Lagar Velho and its Archeological Context*. Trabalhos de Arqueologia 22. Lisbon: Instituto Português de Arqueologia.

Zilhão, J., Davies, S.J.M., Duarte, C., Soares, A.M.M., Steier, P., & Wild, E. (2010). Pego do Diablo (Loures, Portugal): Dating the Emergence of Anatomical Modernity in Westernmost Eurasia. *PLoS ONE,* *5*(1), 1-22.
